# Supplementary material for: Comprehensive Identification of Glycosphingolipids in Human Plasma Using Hydrophilic Interaction Liquid Chromatography—Electrospray Ionization Mass Spectrometry
Source: Metabolites. 2021 Feb 26;11(3):140. doi: 10.3390/metabo11030140 (PMC7996953; doi:10.3390/metabo11030140)
Supplement: Supplementary file 1 [file metabolites-11-00140-s001.pdf]

SUPPLEMENTARY MATERIALS

Comprehensive Identification of Glycosphingolipids in Human Plasma using Hydrophilic Interaction Liquid Chromatography – Electrospray Ionization Mass Spectrometry

Karel Hořejší[a,b], Robert Jirásko[a], Michaela Chocholoušková[a], Denise Wolrab[a], David Kahoun[b], and Michal Holčapek[a, *]

[a] *Department of Analytical Chemistry, Faculty of Chemical Technology, University of Pardubice, Studentská 573, 532 10, Pardubice, Czech Republic*

[b] *Institute of Chemistry, Faculty of Science, University of South Bohemia in České Budějovice, Branišovská 1760, 370 05, České Budějovice, Czech Republic*

*Corresponding author: Michal Holčapek, Department of Analytical Chemistry, Faculty of Chemical Technology, University of Pardubice, Studentská 573, 532 10, Pardubice, Czech Republic, Tel.: +420-466037087; Fax: +420-466037068; E-mail: Michal.Holcapek@upce.cz

Tables: 3

Figures: 131

Table S1. List of used SPE cartridges with more detailed specification.

SPE cartridge	Sorbent Type	Volume [mL]	Sorbent mass [mg]	Carbon load [%]	Vendors
Discovery DSC-8	Silica-based	3	500	9	Supelco
Discovery DSC-18 (A)		3	500	18	Supelco
Discovery DSC-18 (B)		6	500	18	Supelco
Discovery DSC-C18Lt		3	500	11	Supelco
Supelclean ENVI-C18		3	500	17	Supelco
Strata C18-E		3	500	18	Phenomenex
Sep-Pak tC18		3	200	17	Waters
Spe-ed C18/18		3	500	18	Applied Separations
Strata SDB-L	Polymeric-based	3	500	-----	Phenomenex
Strata X		3	500	-----	Phenomenex
Oasis HLB		6	1000	-----	Waters
Diol	Normal phase	3	200	-----	GracePure
Phree	ZrO_2-based	1	30	-----	Phenomenex
HybridSPE		1	30	-----	Merck

Table S2. Characteristic fragment ions (theoretical *m/z*) of selected neutral GSL species with the most common sphingosine base 18:1 that may be observed in positive ion ESI-MS/MS spectra.

Sphingosine base 18:1	SACCHARIDE PART										CERAMIDE PART			Gal-NAc-Gal (Gb₄ fragments)	
	HexCer														
	Z_0	Y_0	$[M+H-H_2O]^+$	$[M+H]^+$											
	Hex_2Cer														
	Z_0	Y_0	Z_1	Y_1	$[M+H-H_2O]^+$	$[M+H]^+$									
	Gb_3														
	Z_0	Y_0	Z_1	Y_1	Z_2	Y_2	$[M+H-H_2O]^+$	$[M+H]^+$							
	Gb_4														
**N-acyl**	Z_0	Y_0	Z_1	Y_1	Z_2	Y_2	Z_3	Y_3	$[M+H-H_2O]^+$	$[M+H]^+$	N^{II}	S	T	B_1	C_1
**12:0**	464.4462	482.4568	626.4990	644.5096	788.5518	806.5624	950.6047	968.6152	1153.6840	1171.6946	264.2656	242.2114	226.2165	366.1395	384.1500
**13:0**	478.4618	496.4724	640.5147	658.5252	802.5675	820.5781	964.6203	982.6309	1167.6997	1185.7103	264.2656	256.2271	240.2322	366.1395	384.1500
**14:0**	492.4775	510.4881	654.5303	672.5409	816.5831	834.5937	978.6360	996.6465	1181.7153	1199.7259	264.2656	270.2427	254.2478	366.1395	384.1500
**15:0**	506.4931	524.5037	668.5460	686.5565	830.5988	848.6094	992.6516	1010.6622	1195.7310	1213.7416	264.2656	284.2584	268.2635	366.1395	384.1500
**15:1**	504.4775	522.4881	666.5303	684.5409	828.5831	846.5937	990.6359	1008.6465	1193.7153	1211.7259	264.2656	282.2427	266.2478	366.1395	384.1500
**15:0 OH**	522.4881	540.4986	684.5409	702.5515	846.5937	864.6043	1008.6465	1026.6571	1211.7259	1229.7365	264.2656	300.2533	284.2584	366.1395	384.1500
**16:0**	520.5088	538.5194	682.5616	700.5722	844.6144	862.6250	1006.6673	1024.6778	1209.7466	1227.7572	264.2656	298.2740	282.2791	366.1395	384.1500
**16:1**	518.4931	536.5037	680.5460	698.5565	842.5988	860.6094	1004.6516	1022.6622	1207.7310	1225.7416	264.2656	296.2584	280.2635	366.1395	384.1500
**16:0 OH**	536.5037	554.5143	698.5565	716.5671	860.6094	878.6199	1022.6622	1040.6728	1225.7416	1243.7521	264.2656	314.2690	298.2740	366.1395	384.1500
**16:1 OH**	534.4881	552.4986	696.5409	714.5515	858.5937	876.6043	1020.6465	1038.6571	1223.7259	1241.7365	264.2656	312.2533	296.2584	366.1395	384.1500
**17:0**	534.5244	552.5350	696.5773	714.5878	858.6301	876.6407	1020.6829	1038.6935	1223.7623	1241.7729	264.2656	312.2897	296.2948	366.1395	384.1500
**17:1**	532.5088	550.5194	694.5616	712.5722	856.6144	874.6250	1018.6672	1036.6778	1221.7466	1239.7572	264.2656	310.2740	294.2791	366.1395	384.1500
**17:0 OH**	550.5194	568.5299	712.5722	730.5828	874.6250	892.6356	1036.6778	1054.6884	1239.7572	1257.7678	264.2656	328.2846	312.2897	366.1395	384.1500
**18:0**	548.5401	566.5507	710.5929	728.6035	872.6457	890.6563	1034.6986	1052.7091	1237.7779	1255.7885	264.2656	326.3053	310.3104	366.1395	384.1500
**18:1**	546.5244	564.5350	708.5773	726.5878	870.6301	888.6407	1032.6829	1050.6935	1235.7623	1253.7729	264.2656	324.2897	308.2948	366.1395	384.1500
**18:2**	544.5088	562.5194	706.5616	724.5722	868.6144	886.6250	1030.6672	1048.6778	1233.7466	1251.7572	264.2656	322.2740	306.2791	366.1395	384.1500
**18:0 OH**	564.5350	582.5456	726.5878	744.5984	888.6407	906.6512	1050.6935	1068.7041	1253.7729	1271.7834	264.2656	342.3003	326.3053	366.1395	384.1500
**18:1 OH**	562.5194	580.5299	724.5722	742.5828	886.6250	904.6356	1048.6778	1066.6884	1251.7572	1269.7678	264.2656	340.2846	324.2897	366.1395	384.1500
**19:0**	562.5557	580.5663	724.6086	742.6191	886.6614	904.6720	1048.7142	1066.7248	1251.7936	1269.8042	264.2656	340.3210	324.3261	366.1395	384.1500
**19:1**	560.5401	578.5507	722.5929	740.6035	884.6457	902.6563	1046.6985	1064.7091	1249.7779	1267.7885	264.2656	338.3053	322.3104	366.1395	384.1500
**19:0 OH**	578.5507	596.5612	740.6035	758.6141	902.6563	920.6669	1064.7091	1082.7197	1267.7885	1285.7991	264.2656	356.3159	340.3210	366.1395	384.1500
**20:0**	576.5714	594.5820	738.6242	756.6348	900.6770	918.6876	1062.7299	1080.7404	1265.8092	1283.8198	264.2656	354.3366	338.3417	366.1395	384.1500
**20:1**	574.5557	592.5663	736.6086	754.6191	898.6614	916.6720	1060.7142	1078.7248	1263.7936	1281.8042	264.2656	352.3210	336.3261	366.1395	384.1500
**20:2**	572.5401	590.5507	734.5929	752.6035	896.6457	914.6563	1058.6985	1076.7091	1261.7779	1279.7885	264.2656	350.3053	334.3104	366.1395	384.1500
**20:0 OH**	592.5663	610.5769	754.6191	772.6297	916.6720	934.6825	1078.7248	1096.7354	1281.8042	1299.8147	264.2656	370.3316	354.3366	366.1395	384.1500
**20:1 OH**	590.5507	608.5612	752.6035	770.6141	914.6563	932.6669	1076.7091	1094.7197	1279.7885	1297.7991	264.2656	368.3159	352.3210	366.1395	384.1500
**21:0**	590.5870	608.5976	752.6399	770.6504	914.6927	932.7033	1076.7455	1094.7561	1279.8249	1297.8355	264.2656	368.3523	352.3574	366.1395	384.1500
**21:1**	588.5714	606.5820	750.6242	768.6348	912.6770	930.6876	1074.7298	1092.7404	1277.8092	1295.8198	264.2656	366.3366	350.3417	366.1395	384.1500
**21:0 OH**	606.5820	624.5925	768.6348	786.6454	930.6876	948.6982	1092.7404	1110.7510	1295.8198	1313.8304	264.2656	384.3472	368.3523	366.1395	384.1500
**22:0**	604.6027	622.6133	766.6555	784.6661	928.7083	946.7189	1090.7612	1108.7717	1293.8405	1311.8511	264.2656	382.3679	366.3730	366.1395	384.1500
**22:1**	602.5870	620.5976	764.6399	782.6504	926.6927	944.7033	1088.7455	1106.7561	1291.8249	1309.8355	264.2656	380.3523	364.3574	366.1395	384.1500
**22:2**	600.5714	618.5820	762.6242	780.6348	924.6770	942.6876	1086.7298	1104.7404	1289.8091	1307.8197	264.2656	378.3365	362.3416	366.1395	384.1500
**22:0 OH**	620.5976	638.6082	782.6504	800.6610	944.7033	962.7138	1106.7561	1124.7667	1309.8355	1327.8460	264.2656	398.3629	382.3679	366.1395	384.1500

**22:1 OH**	618.5820	636.5925	780.6348	798.6454	942.6876	960.6982	1104.7404	1122.7510	1307.8198	1325.8304	264.2656	396.3472	380.3523	366.1395	384.1500
**23:0**	618.6183	636.6289	780.6712	798.6817	942.7240	960.7346	1104.7768	1122.7874	1307.8562	1325.8668	264.2656	396.3836	380.3887	366.1395	384.1500
**23:1**	616.6027	634.6133	778.6555	796.6661	940.7083	958.7189	1102.7611	1120.7717	1305.8405	1323.8511	264.2656	394.3679	378.3730	366.1395	384.1500
**23:0 OH**	634.6133	652.6238	796.6661	814.6767	958.7189	976.7295	1120.7717	1138.7823	1323.8511	1341.8617	264.2656	412.3785	396.3836	366.1395	384.1500
**24:0**	632.6340	650.6446	794.6868	812.6974	956.7396	974.7502	1118.7925	1136.8030	1321.8718	1339.8824	264.2656	410.3992	394.4043	366.1395	384.1500
**24:1**	630.6183	648.6289	792.6712	810.6817	954.7240	972.7346	1116.7768	1134.7874	1319.8562	1337.8668	264.2656	408.3836	392.3887	366.1395	384.1500
**24:2**	628.6027	646.6133	790.6555	808.6661	952.7083	970.7189	1114.7611	1132.7717	1317.8405	1335.8511	264.2656	406.3679	390.3730	366.1395	384.1500
**24:0 OH**	648.6289	666.6395	810.6817	828.6923	972.7346	990.7451	1134.7874	1152.7980	1337.8668	1355.8773	264.2656	426.3942	410.3992	366.1395	384.1500
**24:1 OH**	646.6133	664.6238	808.6661	826.6767	970.7189	988.7295	1132.7717	1150.7823	1335.8511	1353.8617	264.2656	424.3785	408.3836	366.1395	384.1500
**25:0**	646.6496	664.6602	808.7025	826.7130	970.7553	988.7659	1132.8081	1150.8187	1335.8875	1353.8981	264.2656	424.4149	408.4200	366.1395	384.1500
**25:1**	644.6340	662.6446	806.6868	824.6974	968.7396	986.7502	1130.7924	1148.8030	1333.8718	1351.8824	264.2656	422.3992	406.4043	366.1395	384.1500
**25:0 OH**	662.6446	680.6551	824.6974	842.7080	986.7502	1004.7608	1148.8030	1166.8136	1351.8824	1369.8930	264.2656	440.4098	424.4149	366.1395	384.1500
**26:0**	660.6653	678.6759	822.7181	840.7287	984.7709	1002.7815	1146.8238	1164.8343	1349.9031	1367.9137	264.2656	438.4305	422.4356	366.1395	384.1500
**26:1**	658.6496	676.6602	820.7025	838.7130	982.7553	1000.7659	1144.8081	1162.8187	1347.8875	1365.8981	264.2656	436.4149	420.4200	366.1395	384.1500
**26:2**	656.6340	674.6446	818.6868	836.6974	980.7396	998.7502	1142.7924	1160.8030	1345.8718	1363.8824	264.2656	434.3992	418.4043	366.1395	384.1500
**26:0 OH**	676.6602	694.6708	838.7130	856.7236	1000.7659	1018.7764	1162.8187	1180.8293	1365.8981	1383.9086	264.2656	454.4255	438.4305	366.1395	384.1500
**26:1 OH**	674.6446	692.6551	836.6974	854.7080	998.7502	1016.7608	1160.8030	1178.8136	1363.8824	1381.8930	264.2656	452.4098	436.4149	366.1395	384.1500
**27:0**	674.6809	692.6915	836.7338	854.7443	998.7866	1016.7972	1160.8394	1178.8500	1363.9188	1381.9294	264.2656	452.4462	436.4513	366.1395	384.1500
**27:0 OH**	690.6759	708.6864	852.7287	870.7393	1014.7815	1032.7921	1176.8343	1194.8449	1379.9137	1397.9243	264.2656	468.4411	452.4462	366.1395	384.1500
**28:0**	688.6966	706.7072	850.7494	868.7600	1012.8022	1030.8128	1174.8551	1192.8656	1377.9344	1395.9450	264.2656	466.4618	450.4669	366.1395	384.1500
**28:0 OH**	704.6915	722.7021	866.7443	884.7549	1028.7972	1046.8077	1190.8500	1208.8606	1393.9294	1411.9399	264.2656	482.4568	466.4618	366.1395	384.1500

Table S3. List of lipid species identified in human plasma using HILIC-ESI-MS/MS method.

Lipid species	Fatty acyl level	Elemental composition (neutral form)	Theoretical *m/z* of ion	Observed *m/z* of ion	Type of detected ion	Mean absolute intensity	RSD [%]	RT [min]
**Cer[a]**								
32:1		$C_{32}H_{63}NO_3$	510.4881	510.5	$[M+H]^+$	ND	ND	0.80
32:2		$C_{32}H_{61}NO_3$	508.4724	508.5	$[M+H]^+$	ND	ND	0.80
34:1		$C_{34}H_{67}NO_3$	538.5194	538.5	$[M+H]^+$	ND	ND	0.85
34:2		$C_{34}H_{65}NO_3$	536.5037	536.5	$[M+H]^+$	ND	ND	0.80
36:0		$C_{36}H_{73}NO_3$	568.5663	568.5	$[M+H]^+$	ND	ND	0.83
36:1		$C_{36}H_{71}NO_3$	566.5507	566.5	$[M+H]^+$	ND	ND	0.83
38:2		$C_{38}H_{73}NO_3$	592.5663	592.6	$[M+H]^+$	ND	ND	0.81
40:1		$C_{40}H_{79}NO_3$	622.6133	622.6	$[M+H]^+$	ND	ND	0.83
40:2		$C_{40}H_{77}NO_3$	620.5976	620.6	$[M+H]^+$	ND	ND	0.83
41:1		$C_{41}H_{81}NO_3$	636.6289	636.6	$[M+H]^+$	ND	ND	0.83
41:3		$C_{41}H_{77}NO_3$	632.5976	632.6	$[M+H]^+$	ND	ND	0.83
42:1		$C_{42}H_{83}NO_3$	650.6446	650.6	$[M+H]^+$	ND	ND	0.83
42:2		$C_{42}H_{81}NO_3$	648.6289	648.6	$[M+H]^+$	ND	ND	0.83
42:3		$C_{42}H_{79}NO_3$	646.6133	646.6	$[M+H]^+$	ND	ND	0.83
**HexCer**								
34:0[a]		$C_{40}H_{79}NO_8$	702.5878	702.5	$[M+H]^+$	1.54×10^4	20.6	1.12 and 0.97[b]
34:1	18:1/16:0	$C_{40}H_{77}NO_8$	700.5722	700.5	$[M+H]^+$	1.73×10^4	20.1	1.11 and 0.98[b]
34:2	18:1/16:1	$C_{40}H_{75}NO_8$	698.5565	698.5	$[M+H]^+$	2.07×10^4	9.2	1.11 and 0.98[b]
36:0[a]		$C_{42}H_{83}NO_8$	730.6191	730.6	$[M+H]^+$	0.91×10^4	3.0	1.11 and 0.97[b]
36:1	18:1/18:0	$C_{42}H_{81}NO_8$	728.6035	728.5	$[M+H]^+$	2.43×10^4	12.7	1.11 and 0.97[b]
36:2	18:1/18:1	$C_{42}H_{79}NO_8$	726.5878	726.5	$[M+H]^+$	1.00×10^4	4.9	1.11 and 0.97[b]
38:0[a]		$C_{44}H_{89}NO_8$	758.6504	758.6	$[M+H]^+$	0.89×10^4	10.2	1.11 and 0.96[b]
38:1	18:1/20:0<br>16:1/22:0	$C_{44}H_{85}NO_8$	756.6348	756.6	$[M+H]^+$	2.69×10^4	24.3	1.11 and 0.97[b]

38:2	18:1/20:1<br>16:1/22:1	$C_{44}H_{83}NO_8$	754.6191	754.6	$[M+H]^+$	0.90×10^4	10.9	1.12 and 0.97[b]
40:0[a]		$C_{46}H_{91}NO_8$	786.6817	786.6	$[M+H]^+$	1.39×10^4	5.6	1.11 and 0.96[b]
40:1	18:1/22:0	$C_{46}H_{89}NO_8$	784.6661	784.6	$[M+H]^+$	5.37×10^4	18.9	1.11 and 0.96[b]
40:2	18:1/22:1<br>18:2/22:0	$C_{46}H_{87}NO_8$	782.6504	782.6	$[M+H]^+$	2.60×10^4	11.2	1.11 and 0.96[b]
41:1	18:1/23:0	$C_{47}H_{91}NO_8$	798.6817	798.6	$[M+H]^+$	4.47×10^4	17.3	1.11 and 0.96[b]
42:0[a]		$C_{48}H_{95}NO_8$	814.7130	814.6	$[M+H]^+$	1.66×10^4	7.5	1.11 and 0.96[b]
42:1	18:1/24:0	$C_{48}H_{93}NO_8$	812.6974	812.6	$[M+H]^+$	4.97×10^4	22.2	1.11 and 0.96[b]
42:2	18:1/24:1<br>18:2/24:0	$C_{48}H_{91}NO_8$	810.6817	810.6	$[M+H]^+$	5.75×10^4	15.9	1.11 and 0.96[b]
42:3	18:1/24:2<br>18:2/24:1	$C_{48}H_{89}NO_8$	808.6661	808.6	$[M+H]^+$	2.03×10^4	2.7	1.11 and 0.96[b]
**Hex_2Cer**								
32:1	18:1/14:0<br>16:1/16:0	$C_{44}H_{83}NO_{13}$	834.5937	834.5	$[M+H]^+$	3.60×10^4	14.2	1.50
34:1	18:1/16:0	$C_{46}H_{87}NO_{13}$	862.6250	862.6	$[M+H]^+$	37.10×10^4	33.9	1.47
34:2	18:1/16:1<br>18:2/16:0	$C_{46}H_{85}NO_{13}$	860.6094	860.6	$[M+H]^+$	3.71×10^4	8.4	1.47
34:0 OH[a]		$C_{46}H_{89}NO_{14}$	880.6356	880.5	$[M+H]^+$	0.64×10^4	22.1	1.54
34:1 OH	18:1/16:0 OH<br>18:0 OH/16:1	$C_{46}H_{87}NO_{14}$	878.6199	878.5	$[M+H]^+$	0.57×10^4	14.2	1.57
36:1	18:1/18:0	$C_{48}H_{91}NO_{13}$	890.6563	890.6	$[M+H]^+$	0.63×10^4	30.9	1.45
36:2[a]		$C_{48}H_{89}NO_{13}$	888.6407	888.6	$[M+H]^+$	0.50×10^4	25.7	1.44
38:1	18:1/20:0	$C_{50}H_{95}NO_{13}$	918.6876	918.6	$[M+H]^+$	0.44×10^4	32.4	1.42
38:2	18:1/20:1	$C_{50}H_{93}NO_{13}$	916.6720	916.6	$[M+H]^+$	0.21×10^4	28.2	1.43
40:1	18:1/22:0	$C_{52}H_{99}NO_{13}$	946.7189	946.7	$[M+H]^+$	1.00×10^4	36.8	1.40
40:2	18:1/22:1<br>18:2/22:0	$C_{52}H_{97}NO_{13}$	944.7033	944.7	$[M+H]^+$	0.72×10^4	24.2	1.40
40:3	18:1/22:2	$C_{52}H_{95}NO_{13}$	942.6876	942.7	$[M+H]^+$	0.15×10^4	8.4	1.41
42:1		$C_{54}H_{103}NO_{13}$	974.7502	974.7	$[M+H]^+$	1.58×10^4	34.5	1.38
42:2		$C_{54}H_{101}NO_{13}$	972.7346	972.7	$[M+H]^+$	4.55×10^4	28.6	1.38
42:3		$C_{54}H_{99}NO_{13}$	970.7189	970.6	$[M+H]^+$	1.65×10^4	7.0	1.39
**Gb_3**								

32:1		$C_{50}H_{93}NO_{18}$	996.6465	996.6	$[M+H]^+$	0.45×10^4	21.9	2.62
34:1	18:1/16:0	$C_{52}H_{97}NO_{18}$	1024.6778	1024.6	$[M+H]^+$	8.42×10^4	29.7	2.56
34:2		$C_{52}H_{95}NO_{18}$	1022.6622	1022.6	$[M+H]^+$	0.71×10^4	1.4	2.57
34:0 OH		$C_{52}H_{99}NO_{19}$	1042.6884	1042.6	$[M+H]^+$	0.20×10^4	39.5	2.62
34:1 OH[a]<br>35:0[a]		$C_{52}H_{97}NO_{19}$<br>$C_{53}H_{101}NO_{18}$	1040.6728<br>1040.7091	1040.6	$[M+H]^+$	0.13×10^4	24.5	2.72
34:2 OH<br>35:1		$C_{52}H_{95}NO_{19}$<br>$C_{53}H_{99}NO_{18}$	1038.6571<br>1038.6935	1038.6	$[M+H]^+$	1.30×10^4	21.8	2.51
36:1		$C_{54}H_{101}NO_{18}$	1052.7091	1052.7	$[M+H]^+$	0.76×10^4	21.8	2.48
36:2		$C_{54}H_{99}NO_{18}$	1050.6935	1050.7	$[M+H]^+$	0.27×10^4	22.4	2.49
38:1		$C_{56}H_{105}NO_{18}$	1080.7404	1080.7	$[M+H]^+$	0.39×10^4	28.8	2.43
38:2		$C_{56}H_{103}NO_{18}$	1078.7248	1078.7	$[M+H]^+$	0.13×10^4	27.9	2.44
40:1		$C_{58}H_{109}NO_{18}$	1108.7717	1108.7	$[M+H]^+$	0.92×10^4	20.3	2.37
40:2		$C_{58}H_{107}NO_{18}$	1106.7561	1106.7	$[M+H]^+$	0.61×10^4	27.1	2.37
40:3		$C_{58}H_{105}NO_{18}$	1104.7404	1104.7	$[M+H]^+$	0.11×10^4	16.1	2.42
42:1		$C_{60}H_{113}NO_{18}$	1136.8030	1136.7	$[M+H]^+$	0.88×10^4	19.3	2.33
42:2		$C_{60}H_{111}NO_{18}$	1134.7874	1134.7	$[M+H]^+$	1.65×10^4	24.0	2.33
42:3		$C_{60}H_{109}NO_{18}$	1132.7717	1132.7	$[M+H]^+$	0.66×10^4	9.8	2.36
**Gb_4**								
32:1		$C_{58}H_{106}N_2O_{23}$	1199.7259	1199.6	$[M+H]^+$	0.22×10^4	24.5	7.06 and 7.64[c]
33:1[a]		$C_{59}H_{108}N_2O_{23}$	1213.7416	1213.7	$[M+H]^+$	0.08×10^4	34.3	6.95 and 7.58[c]
34:1	18:1/16:0	$C_{60}H_{110}N_2O_{23}$	1227.7572	1227.7	$[M+H]^+$	3.26×10^4	37.8	6.85 and 7.47[c]
34:2		$C_{60}H_{108}N_2O_{23}$	1225.7416	1225.7	$[M+H]^+$	0.34×10^4	9.6	6.91 and 7.48[c]
34:1 OH[a]<br>35:0[a]		$C_{60}H_{110}N_2O_{24}$<br>$C_{61}H_{114}N_2O_{23}$	1243.7521<br>1243.7885	1243.7	$[M+H]^+$	0.08×10^4	22.7	6.87 and 7.39[c]
34:2 OH[a]<br>35:1[a]		$C_{60}H_{108}N_2O_{24}$<br>$C_{61}H_{112}N_2O_{23}$	1241.7365<br>1241.7729	1241.7	$[M+H]^+$	0.05×10^4	34.3	6.75 and 7.48[c]
36:1		$C_{62}H_{114}N_2O_{23}$	1255.7885	1255.8	$[M+H]^+$	0.21×10^4	32.0	6.65
36:2[a]		$C_{62}H_{112}N_2O_{23}$	1253.7729	1253.8	$[M+H]^+$	0.10×10^4	21.9	6.69
38:1		$C_{64}H_{118}N_2O_{23}$	1283.8198	1283.8	$[M+H]^+$	0.10×10^4	25.7	6.43
38:2[a]		$C_{64}H_{116}N_2O_{23}$	1281.8042	1281.8	$[M+H]^+$	0.06×10^4	21.3	6.52
40:1		$C_{66}H_{122}N_2O_{23}$	1311.8511	1311.8	$[M+H]^+$	0.14×10^4	20.0	6.30
40:2		$C_{66}H_{120}N_2O_{23}$	1309.8355	1309.8	$[M+H]^+$	0.11×10^4	13.6	6.31
40:3[a]		$C_{66}H_{118}N_2O_{23}$	1307.8198	1307.8	$[M+H]^+$	0.07×10^4	18.4	6.32
42:1		$C_{68}H_{126}N_2O_{23}$	1339.8824	1339.8	$[M+H]^+$	0.13×10^4	16.9	6.20

42:2		$C_{68}H_{124}N_2O_{23}$	1337.8668	1337.8	$[M+H]^+$	0.27×10^4	17.1	6.23
42:3		$C_{68}H_{122}N_2O_{23}$	1335.8511	1338.5	$[M+H]^+$	0.12×10^4	3.9	6.25
**SHexCer**								
32:1 OH	16:1/16:0 OH<br>18:1/14:0 OH	$C_{38}H_{73}NO_{12}S$	766.4781	766.4	$[M-H]^-$	0.50×10^5	9.9	0.55
34:1	18:1/16:0	$C_{40}H_{77}NO_{11}S$	778.5145	778.5	$[M-H]^-$	3.77×10^5	26.1	0.54
34:2	18:2/16:0	$C_{40}H_{75}NO_{11}S$	776.4988	776.5	$[M-H]^-$	0.49×10^5	21.1	0.54
34:0 OH	18:0/16:0 OH	$C_{40}H_{79}NO_{12}S$	796.5250	796.5	$[M-H]^-$	2.07×10^5	20.5	0.55
34:0 2OH	18:0 OH/16:0 OH	$C_{40}H_{79}NO_{13}S$	812.5199	812.4	$[M-H]^-$	0.98×10^5	14.3	0.55
34:1 OH	18:1/16:0 OH	$C_{40}H_{77}NO_{12}S$	794.5094	794.4	$[M-H]^-$	8.09×10^5	21.0	0.55
34:2 OH	18:2/16:0 OH	$C_{40}H_{75}NO_{12}S$	792.4937	792.5	$[M-H]^-$	0.98×10^5	10.9	0.55
36:1	18:1/18:0	$C_{42}H_{81}NO_{11}S$	806.5458	806.5	$[M-H]^-$	0.17×10^5	30.7	0.54
36:2	18:2/18:0<br>18:1/18:1	$C_{42}H_{79}NO_{11}S$	804.5301	804.5	$[M-H]^-$	0.13×10^5	21.7	0.55
36:1 OH	18:1/18:0 OH	$C_{42}H_{81}NO_{12}S$	822.5407	822.5	$[M-H]^-$	0.31×10^5	27.6	0.55
36:2 OH	18:1/18:1 OH<br>18:2/18:0 OH	$C_{42}H_{79}NO_{12}S$	820.5250	820.5	$[M-H]^-$	0.16×10^5	2.2	0.54
38:1	18:1/20:0	$C_{44}H_{85}NO_{11}S$	834.5771	834.5	$[M-H]^-$	0.31×10^5	19.7	0.54
38:2	18:2/20:0	$C_{44}H_{83}NO_{11}S$	832.5614	832.5	$[M-H]^-$	0.14×10^5	18.7	0.54
38:1 OH	18:1/20:0 OH<br>16:1/22:0 OH	$C_{44}H_{85}NO_{12}S$	850.5720	850.5	$[M-H]^-$	0.32×10^5	14.6	0.54
38:2 OH	18:1/20:1 OH<br>18:2/20:0 OH	$C_{44}H_{83}NO_{12}S$	848.5563	848.5	$[M-H]^-$	0.16×10^5	17.8	0.54
39:1 OH	16:1/23:0 OH<br>18:1/21:0 OH	$C_{45}H_{87}NO_{12}S$	864.5876	864.5	$[M-H]^-$	0.44×10^5	12.3	0.54
40:1	18:1/22:0	$C_{46}H_{89}NO_{11}S$	862.6084	862.5	$[M-H]^-$	0.79×10^5	11.7	0.54
40:2	18:2/22:0	$C_{46}H_{87}NO_{11}S$	860.5927	860.5	$[M-H]^-$	0.47×10^5	21.8	0.54
40:0 OH[a]		$C_{46}H_{91}NO_{12}S$	880.6189	880.6	$[M-H]^-$	0.61×10^5	5.8	0.54
40:0 2OH	18:0 OH/22:0 OH	$C_{46}H_{91}NO_{13}S$	896.6138	896.6	$[M-H]^-$	0.40×10^5	13.2	0.54
40:1 OH	18:1/22:0 OH	$C_{46}H_{89}NO_{12}S$	878.6033	878.5	$[M-H]^-$	1.90×10^5	7.5	0.54
40:2 OH	18:2/22:0 OH<br>18:1/22:1 OH	$C_{46}H_{87}NO_{12}S$	876.5876	876.5	$[M-H]^-$	1.09×10^5	20.1	0.54
41:2[a]		$C_{47}H_{89}NO_{11}S$	874.6084	874.5	$[M-H]^-$	0.31×10^5	33.0	0.54
41:0 OH[a]		$C_{47}H_{93}NO_{12}S$	894.6346	894.6	$[M-H]^-$	0.66×10^5	6.1	0.54
41:1 OH	18:1/23:0 OH	$C_{47}H_{91}NO_{12}S$	892.6189	892.6	$[M-H]^-$	1.72×10^5	8.8	0.54
41:2 OH	18:2/23:0 OH<br>18:1/23:1 OH	$C_{47}H_{89}NO_{12}S$	890.6033	890.5	$[M-H]^-$	1.35×10^5	19.7	0.54
42:2	18:1/24:1<br>18:2/24:0	$C_{48}H_{91}NO_{11}S$	888.6240	888.6	$[M-H]^-$	1.27×10^5	27.1	0.54

42:0 OH[a]		$C_{48}H_{95}NO_{12}S$	908.6502	908.6	[M-H]⁻	0.79 × 10^5	12.7	0.54
42:1 OH	18:1/24:0 OH	$C_{48}H_{93}NO_{12}S$	906.6346	906.6	[M-H]⁻	2.72 × 10^5	12.3	0.54
42:1 2OH	18:0 OH/24:1 OH	$C_{48}H_{93}NO_{13}S$	922.6295	922.6	[M-H]⁻	1.02 × 10^5	14.8	0.55
42:2 OH	18:1/24:1 OH<br>18:2/24:0 OH	$C_{48}H_{91}NO_{12}S$	904.6189	904.6	[M-H]⁻	2.90 × 10^5	19.6	0.54
42:3 OH	18:1/24:2 OH<br>18:2/24:1 OH	$C_{48}H_{89}NO_{12}S$	902.6033	902.6	[M-H]⁻	0.63 × 10^5	5.1	0.54
**SHex₂Cer[a]**								
34:1		$C_{46}H_{87}NO_{16}S$	940.5673	940.5	[M-H]⁻	0.21 × 10^5	32.3	0.57
34:1 OH<br>35:0		$C_{46}H_{87}NO_{17}S$<br>$C_{47}H_{91}NO_{16}S$	956.5622<br>956.5986	956.6	[M-H]⁻	0.10 × 10^5	25.5	0.58
36:1		$C_{48}H_{91}NO_{16}S$	968.5986	968.5	[M-H]⁻	0.08 × 10^5	5.7	0.57
36:1 OH<br>37:0		$C_{48}H_{91}NO_{17}S$<br>$C_{49}H_{95}NO_{16}S$	984.5935<br>984.6299	984.5	[M-H]⁻	0.07 × 10^5	26.6	0.55
38:1		$C_{50}H_{95}NO_{16}S$	996.6299	996.6	[M-H]⁻	0.08 × 10^5	47.5	0.57
38:1 OH<br>39:0		$C_{50}H_{95}NO_{17}S$<br>$C_{51}H_{99}NO_{16}S$	1012.6248<br>1012.6612	1012.6	[M-H]⁻	0.07 × 10^5	31.4	0.57
40:1		$C_{52}H_{99}NO_{16}S$	1024.6612	1024.6	[M-H]⁻	0.16 × 10^5	24.3	0.57
40:1 OH<br>41:0		$C_{52}H_{99}NO_{17}S$<br>$C_{53}H_{103}NO_{16}S$	1040.6561<br>1040.6925	1040.6	[M-H]⁻	0.11 × 10^5	39.6	0.57
40:2 OH<br>41:1		$C_{52}H_{97}NO_{17}S$<br>$C_{53}H_{101}NO_{16}S$	1038.6404<br>1038.6768	1038.6	[M-H]⁻	0.12 × 10^5	20.7	0.57
42:1		$C_{54}H_{103}NO_{16}S$	1052.6925	1052.7	[M-H]⁻	0.19 × 10^5	33.9	0.56
42:2		$C_{54}H_{101}NO_{16}S$	1050.6768	1050.6	[M-H]⁻	0.20 × 10^5	15.6	0.57
42:1 OH<br>43:0		$C_{54}H_{103}NO_{17}S$<br>$C_{55}H_{107}NO_{16}S$	1068.6874<br>1068.7238	1068.7	[M-H]⁻	0.11 × 10^5	18.9	0.57
42:2 OH<br>43:1		$C_{54}H_{101}NO_{17}S$<br>$C_{55}H_{105}NO_{16}S$	1066.6717<br>1066.7081	1066.6	[M-H]⁻	0.11 × 10^5	18.5	0.57
**GM₃**								
30:1		$C_{53}H_{96}N_2O_{21}$	1095.6433	1095.6	[M-H]⁻	0.11 × 10^5	12.7	2.55
32:1		$C_{55}H_{100}N_2O_{21}$	1123.6746	1123.7	[M-H]⁻	0.48 × 10^5	8.19	2.46
34:1		$C_{57}H_{104}N_2O_{21}$	1151.7059	1151.7	[M-H]⁻	4.72 × 10^5	24.3	2.40
34:2		$C_{57}H_{102}N_2O_{21}$	1149.6902	1149.7	[M-H]⁻	0.49 × 10^5	16.2	2.40
34:1 OH		$C_{57}H_{104}N_2O_{22}$	1167.7008	1167.7	[M-H]⁻	0.68 × 10^5	30.2	2.37
34:2 OH		$C_{57}H_{102}N_2O_{22}$	1165.6852	1165.7	[M-H]⁻	0.52 × 10^5	26.9	2.36
36:1		$C_{59}H_{108}N_2O_{21}$	1179.7372	1179.8	[M-H]⁻	3.04 × 10^5	28.3	2.33
36:2		$C_{59}H_{106}N_2O_{21}$	1177.7215	1177.8	[M-H]⁻	0.51 × 10^5	3.3	2.35
36:1 OH		$C_{59}H_{108}N_2O_{22}$	1195.7321	1195.8	[M-H]⁻	0.35 × 10^5	24.3	2.32

36:2 OH		$C_{59}H_{106}N_2O_{22}$	1193.7165	1193.8	$[M-H]^-$	0.49×10^5	28.5	2.32
38:1		$C_{61}H_{112}N_2O_{21}$	1207.7685	1207.8	$[M-H]^-$	1.53×10^5	29.3	2.26
38:2		$C_{61}H_{110}N_2O_{21}$	1205.7528	1205.8	$[M-H]^-$	0.33×10^5	3.5	2.28
38:1 OH		$C_{61}H_{112}N_2O_{22}$	1223.7634	1223.8	$[M-H]^-$	0.42×10^5	36.6	2.26
38:2 OH		$C_{61}H_{110}N_2O_{22}$	1221.7478	1221.8	$[M-H]^-$	0.62×10^5	30.1	2.26
40:1		$C_{63}H_{116}N_2O_{21}$	1235.7998	1235.8	$[M-H]^-$	2.65×10^5	21.3	2.21
40:2		$C_{63}H_{114}N_2O_{21}$	1233.7841	1233.8	$[M-H]^-$	1.07×10^5	9.0	2.22
40:1 OH		$C_{63}H_{116}N_2O_{22}$	1251.7947	1251.8	$[M-H]^-$	0.93×10^5	21.5	2.19
40:2 OH		$C_{63}H_{114}N_2O_{22}$	1249.7791	1249.8	$[M-H]^-$	1.40×10^5	20.3	2.19
40:3 OH		$C_{63}H_{112}N_2O_{22}$	1247.7634	1247.8	$[M-H]^-$	0.52×10^5	13.3	2.20
42:1		$C_{65}H_{120}N_2O_{21}$	1263.8311	1263.8	$[M-H]^-$	2.16×10^5	19.2	2.17
42:2		$C_{65}H_{118}N_2O_{21}$	1261.8154	1261.8	$[M-H]^-$	2.59×10^5	15.2	2.17
42:3		$C_{65}H_{116}N_2O_{21}$	1259.7998	1259.8	$[M-H]^-$	0.73×10^5	11.0	2.20
42:1 OH		$C_{65}H_{120}N_2O_{22}$	1279.8260	1279.8	$[M-H]^-$	1.07×10^5	20.7	2.34
42:2 OH		$C_{65}H_{118}N_2O_{22}$	1277.8103	1277.9	$[M-H]^-$	1.05×10^5	21.4	2.35
42:3 OH		$C_{65}H_{116}N_2O_{22}$	1275.7947	1275.8	$[M-H]^-$	0.31×10^5	0.7	2.36
**PI**								
32:0	16:0/16:0	$C_{41}H_{79}O_{13}P$	809.5186	809.5	$[M-H]^-$	0.16×10^6	22.4	1.25
32:1	16:0/16:1	$C_{41}H_{77}O_{13}P$	807.5029	807.5	$[M-H]^-$	0.23×10^6	5.4	1.24
34:1	16:0/18:1<br>18:0/16:1	$C_{43}H_{81}O_{13}P$	835.5342	835.6	$[M-H]^-$	1.86×10^6	9.2	1.22
34:2	16:0/18:2	$C_{43}H_{79}O_{13}P$	833.5186	833.6	$[M-H]^-$	1.02×10^6	5.1	1.22
35:1	17:0/18:1	$C_{44}H_{83}O_{13}P$	849.5499	849.6	$[M-H]^-$	0.09×10^6	10.9	1.21
35:2	17:0/18:2	$C_{44}H_{81}O_{13}P$	847.5342	847.6	$[M-H]^-$	0.07×10^6	4.4	1.21
36:1	18:0/18:1	$C_{44}H_{85}O_{13}P$	863.5655	863.5	$[M-H]^-$	1.77×10^6	18.1	1.21
36:2	18:0/18:2<br>18:1/18:1	$C_{45}H_{83}O_{13}P$	861.5499	861.6	$[M-H]^-$	3.01×10^6	11.1	1.21
36:3	16:0/20:3<br>18:1/18:2	$C_{45}H_{81}O_{13}P$	859.5342	859.5	$[M-H]^-$	0.66×10^6	6.6	1.20
36:4	16:0/20:4	$C_{45}H_{79}O_{13}P$	857.5186	857.6	$[M-H]^-$	1.31×10^6	7.6	1.19
38:3	18:0/20:3	$C_{47}H_{85}O_{13}P$	887.5655	887.6	$[M-H]^-$	4.19×10^6	14.6	1.18
38:4	18:0/20:4	$C_{47}H_{83}O_{13}P$	885.5499	885.6	$[M-H]^-$	14.23×10^6	11.6	1.17
38:5	18:1/20:4<br>18:0/20:5<br>16:0/22:5	$C_{47}H_{81}O_{13}P$	883.5342	883.5	$[M-H]^-$	0.51×10^6	8.5	1.18
38:6	16:0/22:6	$C_{47}H_{79}O_{13}P$	881.5186	881.5	$[M-H]^-$	0.21×10^6	15.0	1.18
40:4	18:0/22:4	$C_{49}H_{87}O_{13}P$	913.5812	913.5	$[M-H]^-$	0.30×10^6	14.3	1.17
40:5	18:0/22:5	$C_{49}H_{85}O_{13}P$	911.5655	911.5	$[M-H]^-$	0.68×10^6	14.0	1.17

40:6	18:0/22:6	$C_{49}H_{83}O_{13}P$	909.5499	909.5	$[M-H]^-$	0.66×10^6	10.6	1.16
**1-LPI**								
16:0/0:0		$C_{25}H_{49}O_{12}P$	571.2889	571.4	$[M-H]^-$	0.79×10^5	5.0	2.15
16:1/0:0		$C_{25}H_{47}O_{12}P$	569.2732	569.3	$[M-H]^-$	0.28×10^5	19.9	2.17
18:0/0:0		$C_{27}H_{53}O_{12}P$	599.3202	599.4	$[M-H]^-$	4.44×10^5	24.9	2.07
18:1/0:0		$C_{27}H_{51}O_{12}P$	597.3045	597.4	$[M-H]^-$	1.80×10^5	13.8	2.07
18:2/0:0		$C_{27}H_{49}O_{12}P$	595.2889	595.3	$[M-H]^-$	1.36×10^5	12.9	2.12
20:3/0:0		$C_{29}H_{51}O_{12}P$	621.3045	621.4	$[M-H]^-$	1.40×10^5	12.7	2.03
20:4/0:0		$C_{29}H_{49}O_{12}P$	619.2889	619.3	$[M-H]^-$	6.23×10^5	13.4	2.02
22:4/0:0[a]		$C_{31}H_{53}O_{12}P$	647.3202	647.4	$[M-H]^-$	0.09×10^5	11.1	1.98
22:5/0:0		$C_{31}H_{51}O_{12}P$	645.3045	645.3	$[M-H]^-$	0.31×10^5	18.2	2.00
22:6/0:0		$C_{31}H_{49}O_{12}P$	643.2889	643.3	$[M-H]^-$	0.36×10^5	14.9	1.98
**2-LPI**								
0:0/16:0		$C_{25}H_{49}O_{12}P$	571.2889	571.4	$[M-H]^-$	0.50×10^5	26.7	1.93
0:0/16:1		$C_{25}H_{47}O_{12}P$	569.2732	569.3	$[M-H]^-$	0.06×10^5	34.3	1.95
0:0/18:0		$C_{27}H_{53}O_{12}P$	599.3202	599.4	$[M-H]^-$	0.85×10^5	13.1	1.85
0:0/18:1		$C_{27}H_{51}O_{12}P$	597.3045	597.4	$[M-H]^-$	0.38×10^5	14.5	1.88
0:0/18:2		$C_{27}H_{49}O_{12}P$	595.2889	595.3	$[M-H]^-$	0.17×10^5	11.6	1.88
0:0/20:3		$C_{29}H_{51}O_{12}P$	621.3045	621.4	$[M-H]^-$	0.47×10^5	10.1	1.82
0:0/20:4		$C_{29}H_{49}O_{12}P$	619.2889	619.3	$[M-H]^-$	2.06×10^5	10.4	1.80
0:0/22:4[a]		$C_{31}H_{53}O_{12}P$	647.3202	647.4	$[M-H]^-$	0.10×10^5	9.6	1.82
0:0/22:5		$C_{31}H_{51}O_{12}P$	645.3045	645.3	$[M-H]^-$	0.22×10^5	25.0	1.81
0:0/22:6		$C_{31}H_{49}O_{12}P$	643.2889	643.3	$[M-H]^-$	0.28×10^5	12.7	1.81
**PE[a]**								
34:0		$C_{39}H_{78}NO_8P$	718.5392	718.5	$[M-H]^-$	0.08×10^5	3.0	5.37
34:1		$C_{39}H_{76}NO_8P$	716.5236	716.5	$[M-H]^-$	0.52×10^5	6.2	5.37
34:2		$C_{39}H_{74}NO_8P$	714.5079	714.5	$[M-H]^-$	0.84×10^5	4.8	5.38
34:3		$C_{39}H_{72}NO_8P$	712.4923	712.5	$[M-H]^-$	0.08×10^5	7.0	5.39
36:1		$C_{41}H_{80}NO_8P$	744.5549	744.5	$[M-H]^-$	0.52×10^5	14.8	5.22
36:2		$C_{41}H_{78}NO_8P$	742.5392	742.5	$[M-H]^-$	1.58×10^5	10.2	5.23
36:3		$C_{41}H_{76}NO_8P$	740.5236	740.5	$[M-H]^-$	0.82×10^5	11.4	5.21
36:4		$C_{41}H_{74}NO_8P$	738.5079	738.5	$[M-H]^-$	1.84×10^5	9.0	5.02
36:5		$C_{41}H_{72}NO_8P$	736.4923	736.5	$[M-H]^-$	0.39×10^5	10.1	5.07
38:1		$C_{43}H_{84}NO_8P$	772.5862	772.5	$[M-H]^-$	1.37×10^5	13.2	4.53
38:2		$C_{43}H_{82}NO_8P$	770.5705	770.5	$[M-H]^-$	0.14×10^5	1.9	4.56
38:3		$C_{43}H_{80}NO_8P$	768.5549	768.5	$[M-H]^-$	0.64×10^5	11.3	4.89
38:4		$C_{43}H_{78}NO_8P$	766.5392	766.5	$[M-H]^-$	3.50×10^5	10.4	4.89
38:5		$C_{43}H_{76}NO_8P$	764.5236	764.5	$[M-H]^-$	1.58×10^5	8.8	4.90

38:6		$C_{43}H_{74}NO_8P$	762.5079	762.5	$[M-H]^-$	2.23×10^5	8.2	4.94
40:4		$C_{45}H_{82}NO_8P$	794.5705	794.5	$[M-H]^-$	0.11×10^5	9.9	4.79
40:5		$C_{45}H_{80}NO_8P$	792.5549	792.5	$[M-H]^-$	0.40×10^5	11.8	4.79
40:6		$C_{45}H_{78}NO_8P$	790.5392	790.5	$[M-H]^-$	1.08×10^5	9.6	4.80
40:7		$C_{45}H_{76}NO_8P$	788.5236	788.5	$[M-H]^-$	0.30×10^5	8.8	4.79
**1-LPE[a]**								
16:0/0:0		$C_{21}H_{44}NO_7P$	452.2783	452.3	$[M-H]^-$	0.84×10^5	0.6	10.33
18:0/0:0		$C_{23}H_{48}NO_7P$	480.3096	480.4	$[M-H]^-$	0.69×10^5	16.4	10.02
18:1/0:0		$C_{23}H_{46}NO_7P$	478.2939	478.3	$[M-H]^-$	0.86×10^5	14.0	10.08
18:2/0:0		$C_{23}H_{44}NO_7P$	476.2782	476.3	$[M-H]^-$	1.65×10^5	11.6	10.23
20:3/0:0		$C_{25}H_{46}NO_7P$	502.2939	502.3	$[M-H]^-$	0.11×10^5	24.9	9.91
20:4/0:0		$C_{25}H_{44}NO_7P$	500.2783	500.3	$[M-H]^-$	0.57×10^5	31.0	9.88
22:5/0:0		$C_{27}H_{46}NO_7P$	526.2939	526.3	$[M-H]^-$	0.04×10^5	31.3	9.82
22:6/0:0		$C_{27}H_{44}NO_7P$	524.2783	524.3	$[M-H]^-$	0.11×10^5	22.7	9.76
**2-LPE[a]**								
0:0/16:0		$C_{21}H_{44}NO_7P$	452.2783	452.3	$[M-H]^-$	0.17×10^5	6.4	9.77
0:0/18:0		$C_{23}H_{48}NO_7P$	480.3096	480.4	$[M-H]^-$	0.48×10^5	33.7	9.44
0:0/18:1		$C_{23}H_{46}NO_7P$	478.2939	478.3	$[M-H]^-$	0.36×10^5	8.8.	9.49
0:0/18:2		$C_{23}H_{44}NO_7P$	476.2782	476.3	$[M-H]^-$	0.43×10^5	13.0	9.66
0:0/20:3		$C_{25}H_{46}NO_7P$	502.2939	502.3	$[M-H]^-$	0.22×10^5	7.9	9.26
0:0/20:4		$C_{25}H_{44}NO_7P$	500.2783	500.3	$[M-H]^-$	2.21×10^5	7.6	9.23
0:0/22:5		$C_{27}H_{46}NO_7P$	526.2939	526.3	$[M-H]^-$	0.39×10^5	11.2	9.20
0:0/22:6		$C_{27}H_{44}NO_7P$	524.2783	524.3	$[M-H]^-$	1.79×10^5	13.0	9.14
**FA[a]**								
	14:0	$C_{14}H_{28}O_2$	227.2017	227.3	$[M-H]^-$	ND	ND	0.99
	15:0	$C_{15}H_{30}O_2$	241.2173	241.3	$[M-H]^-$	ND	ND	0.99
	16:0	$C_{16}H_{32}O_2$	255.2329	255.3	$[M-H]^-$	ND	ND	0.99
	16:1	$C_{16}H_{30}O_2$	253.2173	253.3	$[M-H]^-$	ND	ND	0.99
	17:0	$C_{17}H_{34}O_2$	269.2486	269.3	$[M-H]^-$	ND	ND	0.98
	17:1	$C_{17}H_{32}O_2$	267.2330	267.3	$[M-H]^-$	ND	ND	0.98
	18:0	$C_{18}H_{36}O_2$	283.2643	283.3	$[M-H]^-$	ND	ND	0.98
	18:1	$C_{18}H_{34}O_2$	281.2486	281.3	$[M-H]^-$	ND	ND	0.98
	18:2	$C_{18}H_{32}O_2$	279.2330	279.3	$[M-H]^-$	ND	ND	0.98
	18:3	$C_{18}H_{30}O_2$	277.2173	277.3	$[M-H]^-$	ND	ND	0.99
	20:3	$C_{20}H_{34}O_2$	305.2486	305.3	$[M-H]^-$	ND	ND	0.98
	20:4	$C_{20}H_{32}O_2$	303.2330	303.3	$[M-H]^-$	ND	ND	0.98
	22:4	$C_{22}H_{36}O_2$	331.2643	331.3	$[M-H]^-$	ND	ND	0.97
	22:5	$C_{22}H_{34}O_2$	329.2486	329.3	$[M-H]^-$	ND	ND	0.98

	22:6	$C_{22}H_{32}O_2$	327.2330	327.3	$[M-H]^-$	ND	ND	0.98

RT = retention time, ND = not determined

[a] Lipid species that could not be confirmed by MS/MS experiments but their presence in human plasma can be predicted based on measured MS spectra and their earlier identification in previous works.

[b] Two retention times are stated due to probable presence of GalCer and GlcCer species.

[c] Two retention times are reported due to separation of Gb_4 subclass into peaks corresponding to the short and long N-acyl chains, and probably caused by isomerism, as stated in the text.

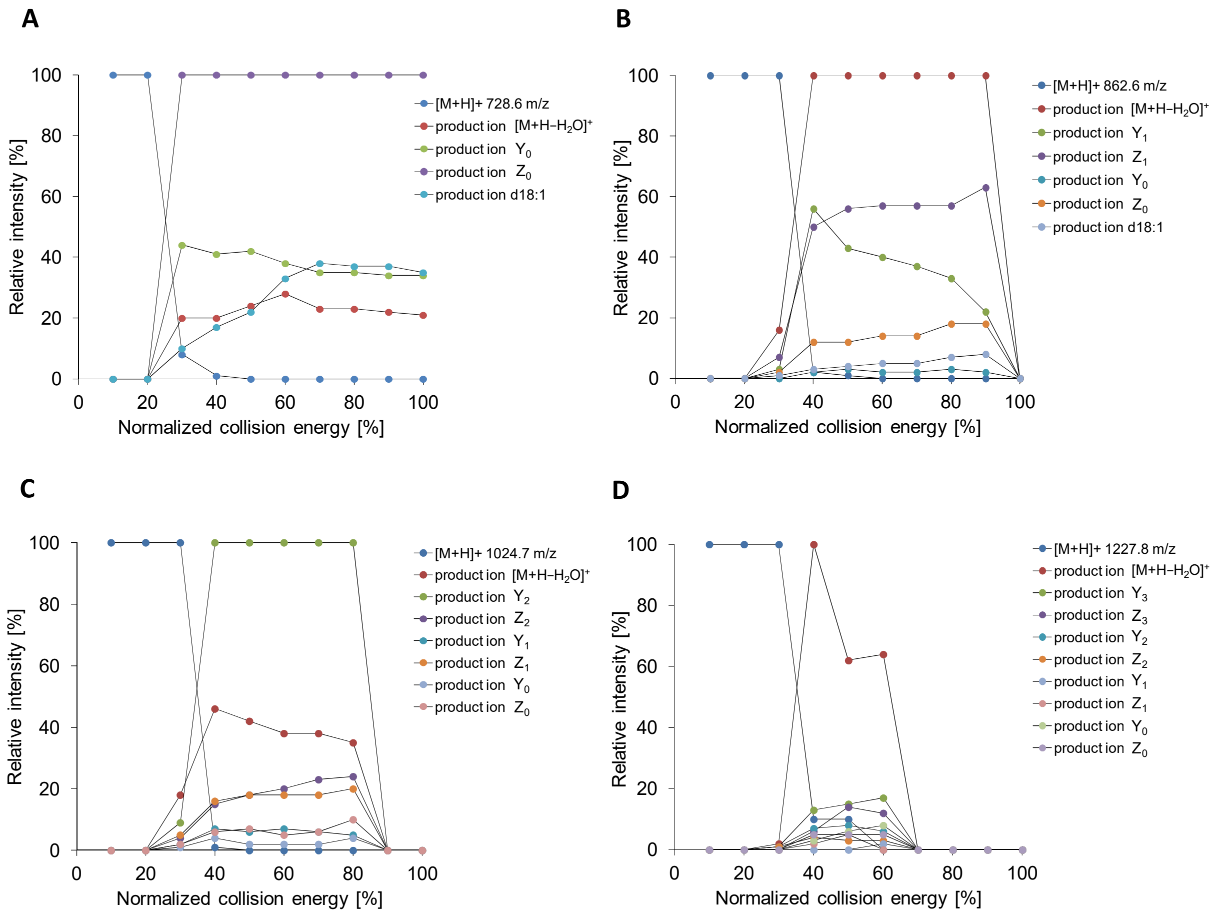

Figure S1. Optimization of normalized collision energy (NCE) for neutral GSL subclasses using the commercially available TLC neutral GSL mixture. The graphs display the relative intensity of the precursor and product ions obtained by MS/MS analysis of **(A)** HexCer 18:1/16:0 at *m/z* 700.6, **(B)** Hex_2Cer 18:1/16:0 at *m/z* 862.6, **(C)** Gb_3 18:1/16:0 at *m/z* 1024.7, and **(D)** Gb_4 18:1/16:0 at *m/z* 1227.8.

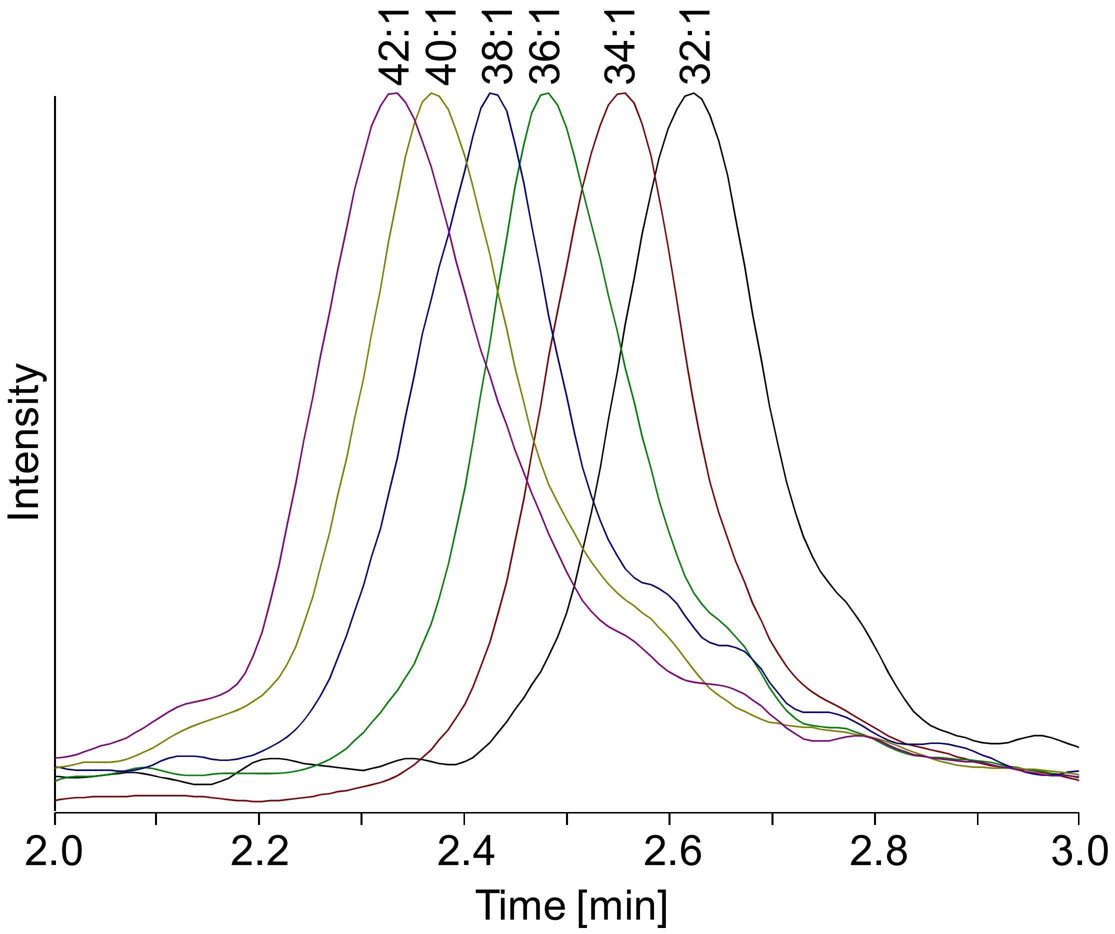

Figure S2. Overlay of reconstructed ion current (RIC) chromatograms of individual glycosphingolipid species within Gb_3 subclass with the number of carbon atoms and double bonds in the fatty acyl chain.

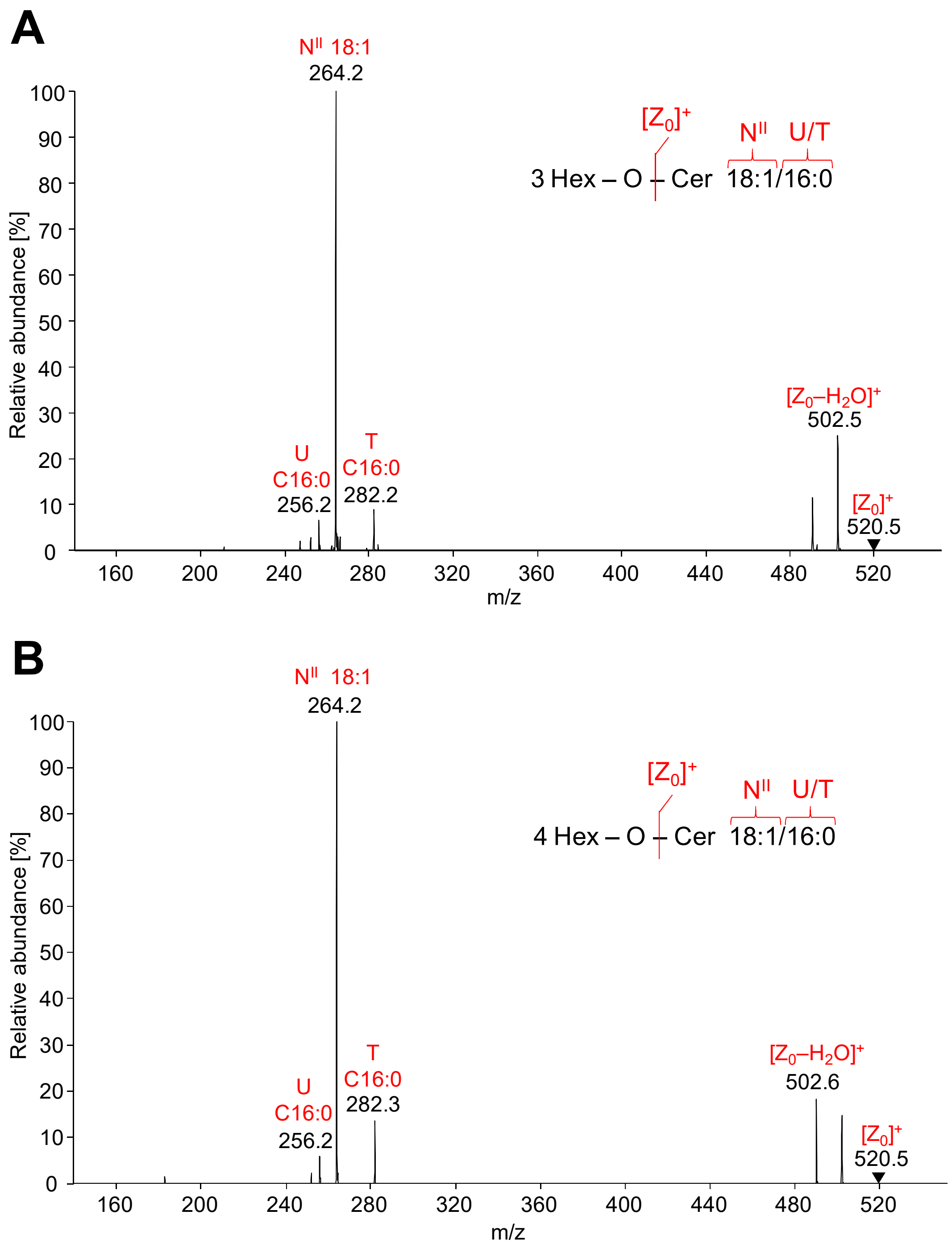

Figure S3. HILIC-ESI/MS^3 spectra of product ion $[Z_0]^+$ at *m/z* 520.5 for **(A)** Gb_3 18:1/16:0 and **(B)** Gb_4 18:1/16:0 lipid species. MS^3 spectra confirm the specific composition of ceramide part, which consists of sphingosine base 18:1 and N-linked fatty acyl 16:0.

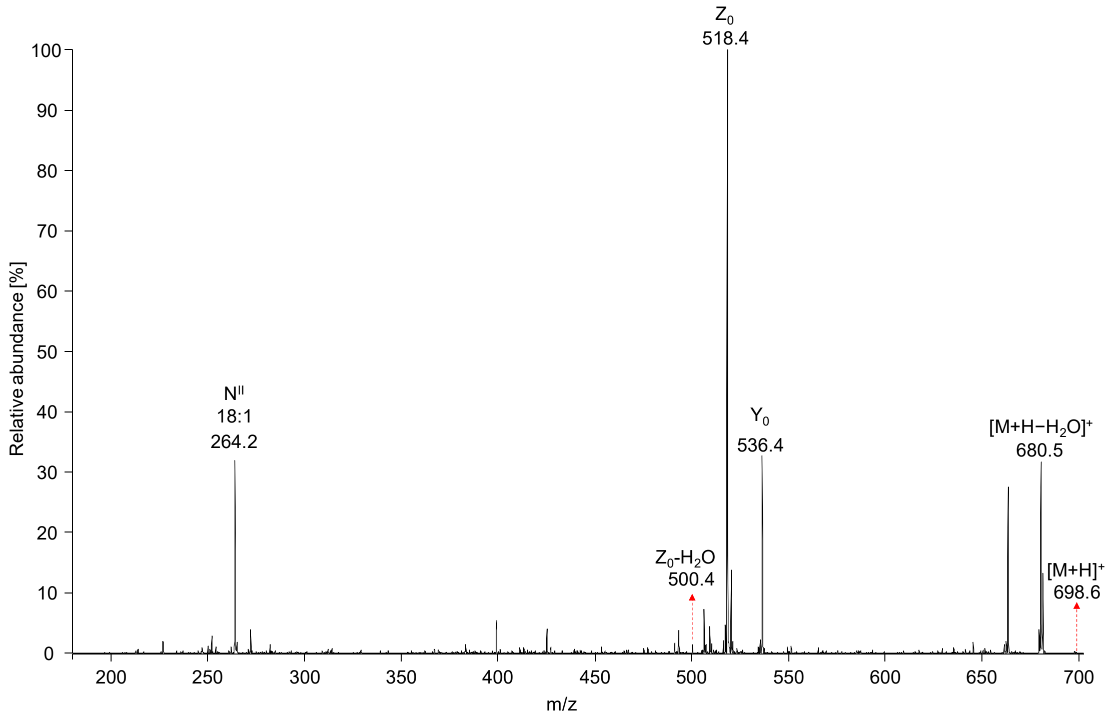

Figure S4. MS/MS spectrum of HexCer 34:2 at *m/z* 698.6 with characteristic fragments.

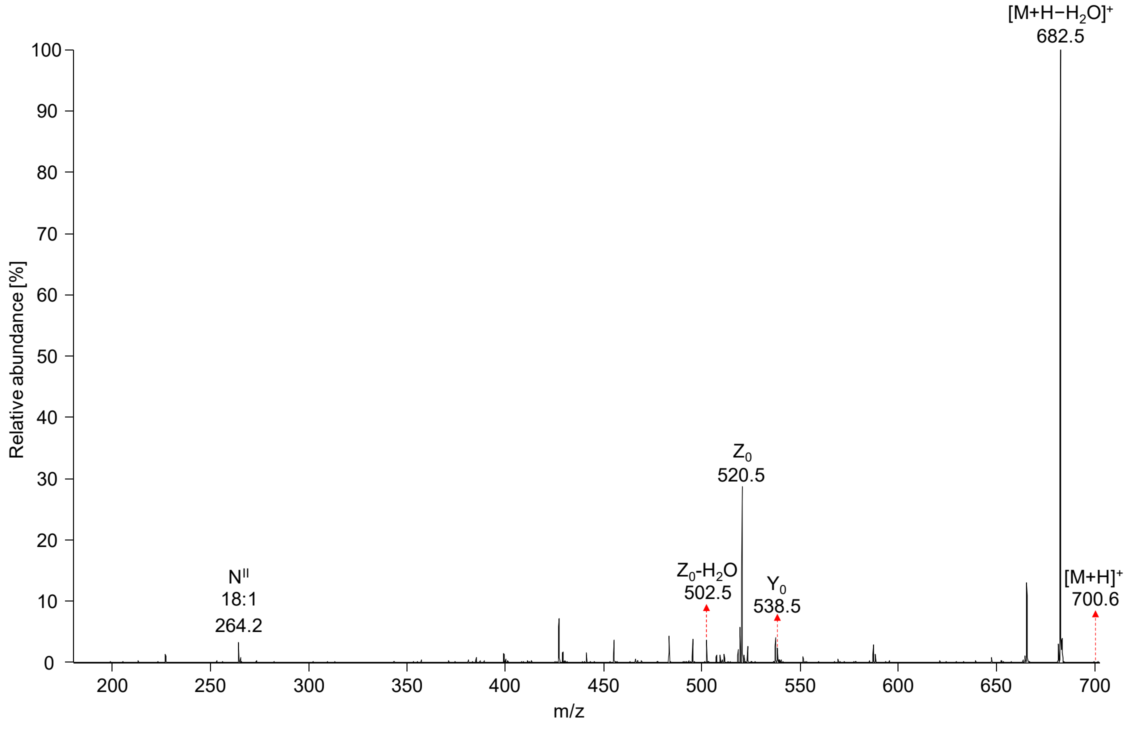

Figure S5. MS/MS spectrum of HexCer 34:1 at *m/z* 700.6 with characteristic fragments.

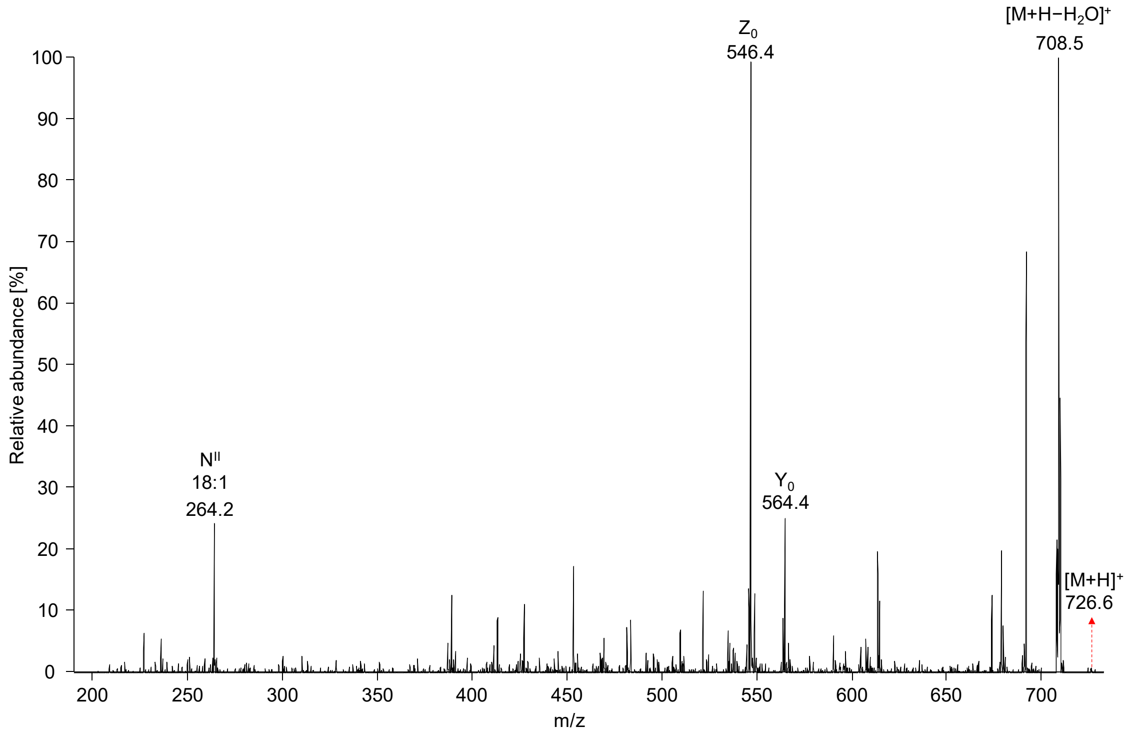

Figure S6. MS/MS spectrum of HexCer 36:2 at *m/z* 726.6 with characteristic fragments.

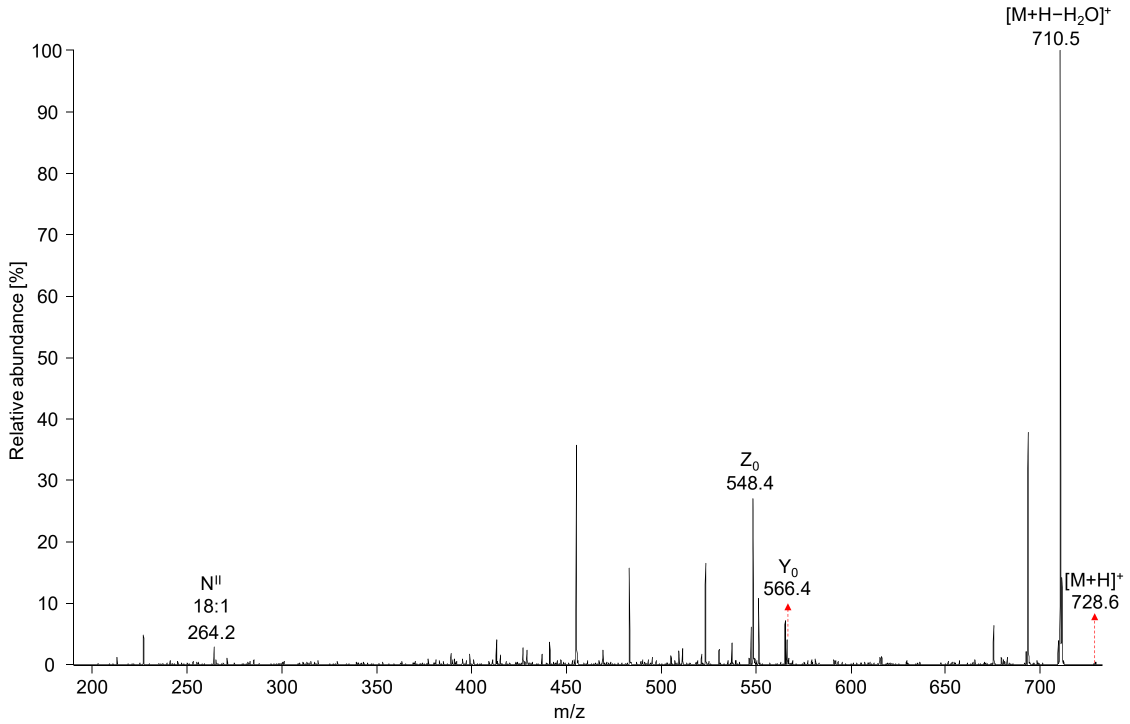

Figure S7. MS/MS spectrum of HexCer 36:1 at *m/z* 728.6 with characteristic fragments.

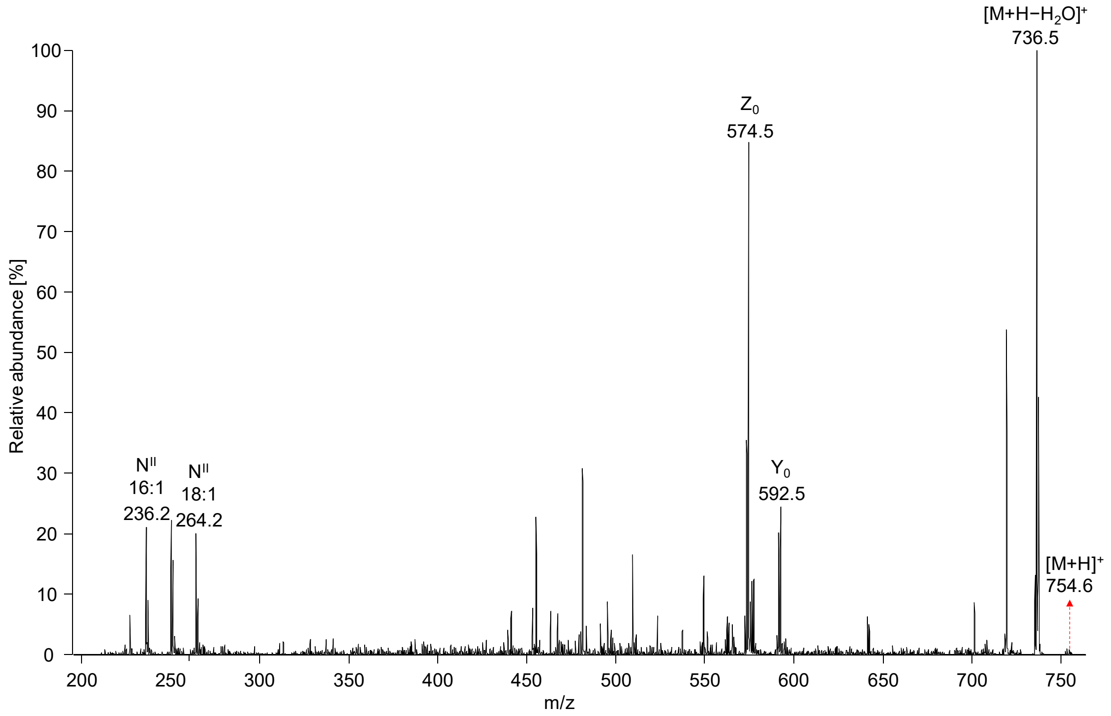

Figure S8. MS/MS spectrum of HexCer 38:2 at *m/z* 754.6 with characteristic fragments.

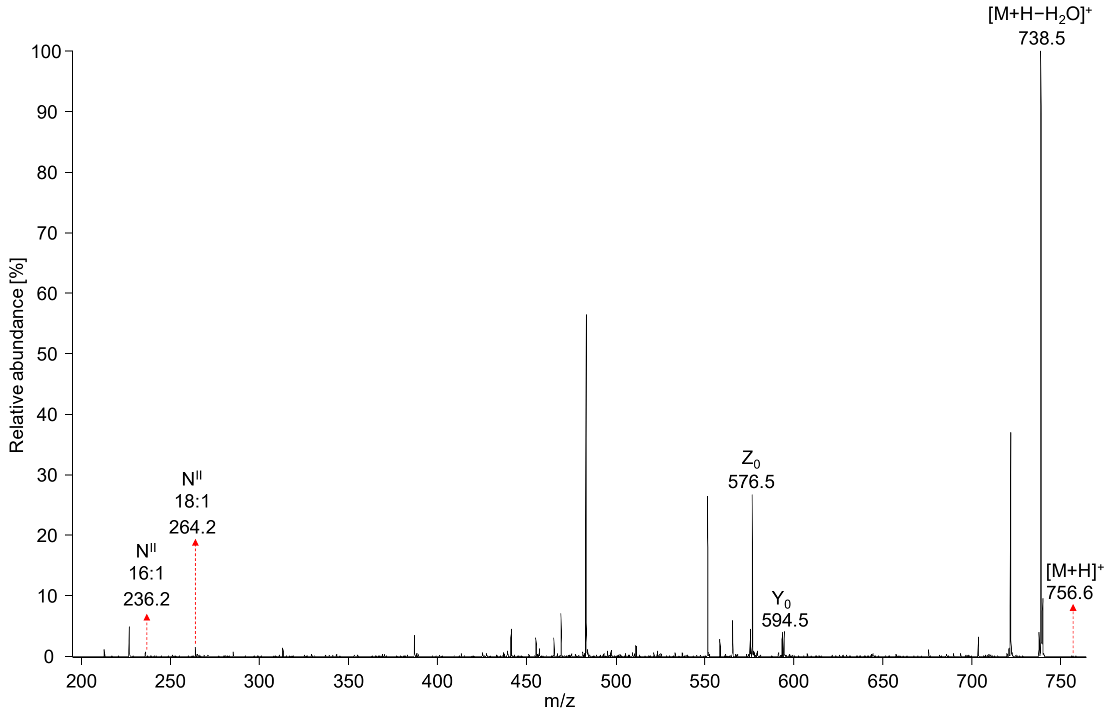

Figure S9. MS/MS spectrum of HexCer 38:1 at *m/z* 756.6 with characteristic fragments.

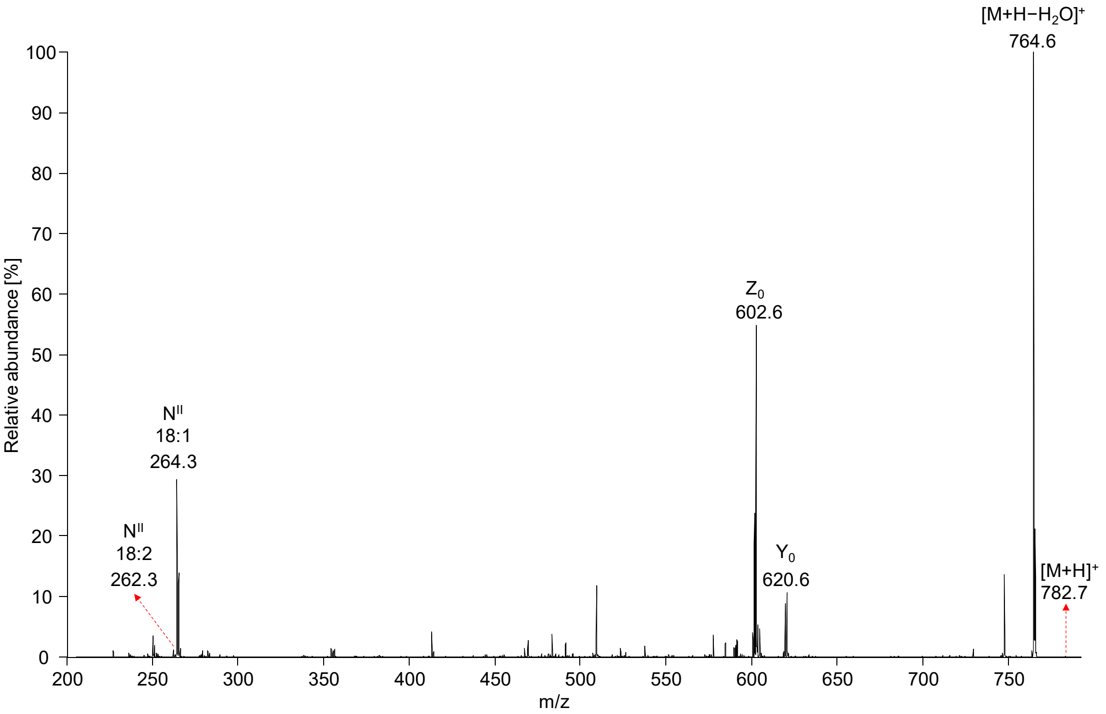

Figure S10. MS/MS spectrum of HexCer 40:2 at *m/z* 782.7 with characteristic fragments.

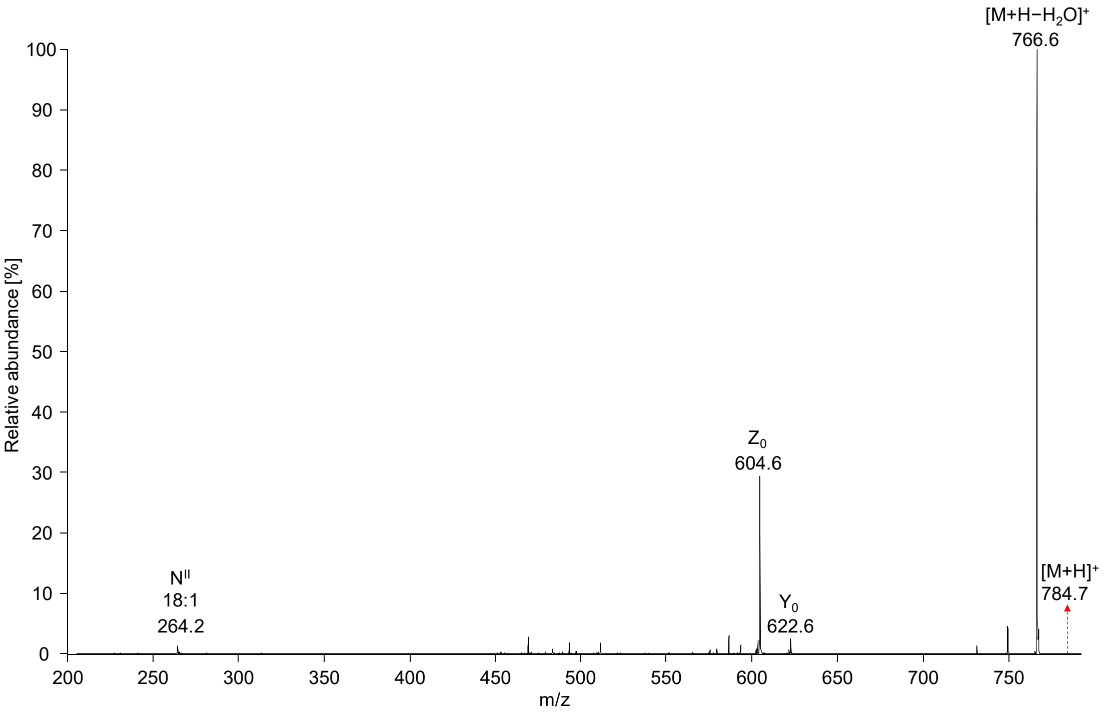

Figure S11. MS/MS spectrum of HexCer 40:1 at *m/z* 784.7 with characteristic fragments.

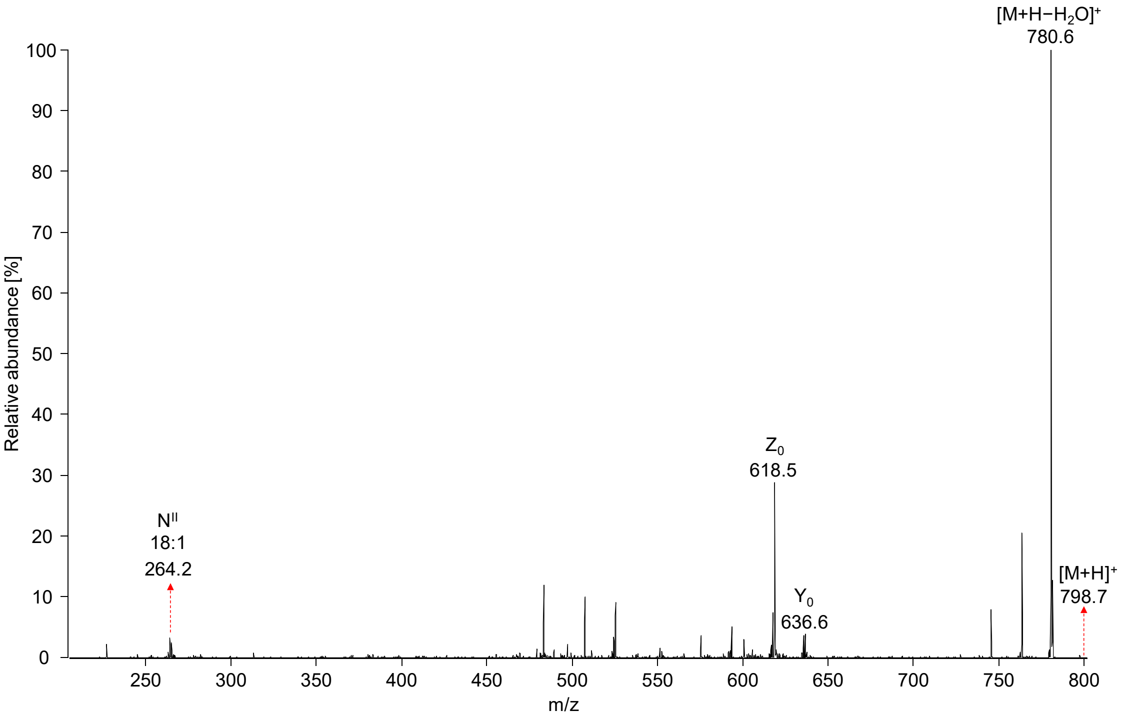

Figure S12. MS/MS spectrum of HexCer 41:1 at *m/z* 798.7 with characteristic fragments.

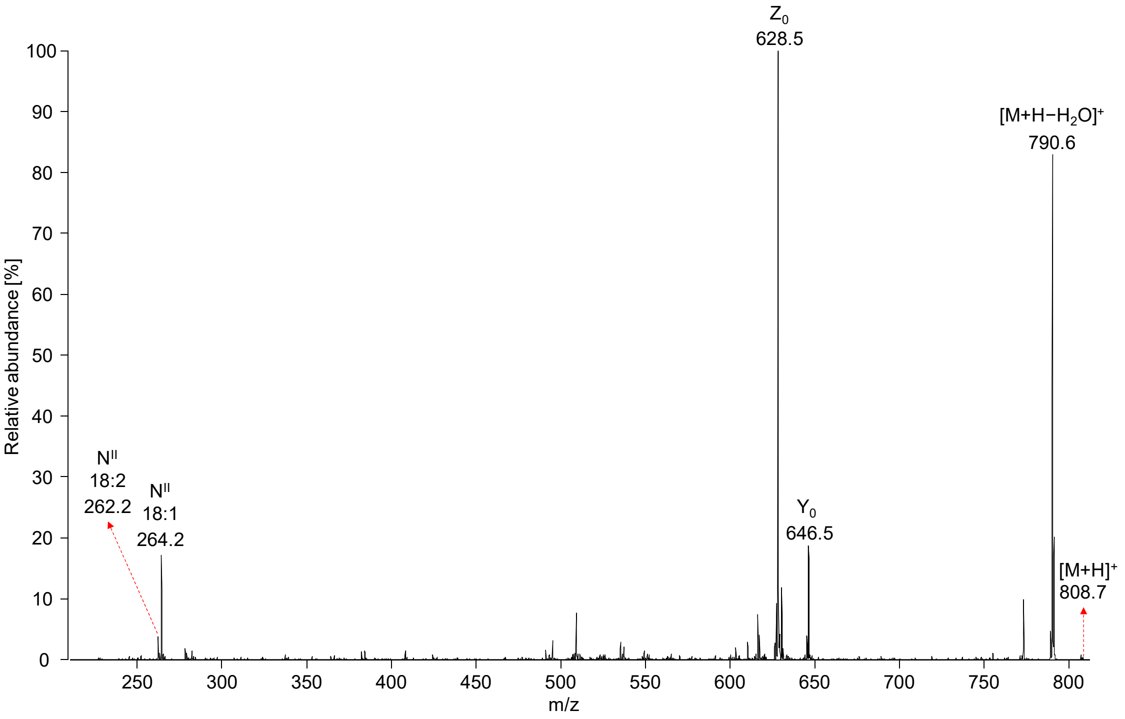

Figure S13. MS/MS spectrum of HexCer 42:3 at *m/z* 808.7 with characteristic fragments.

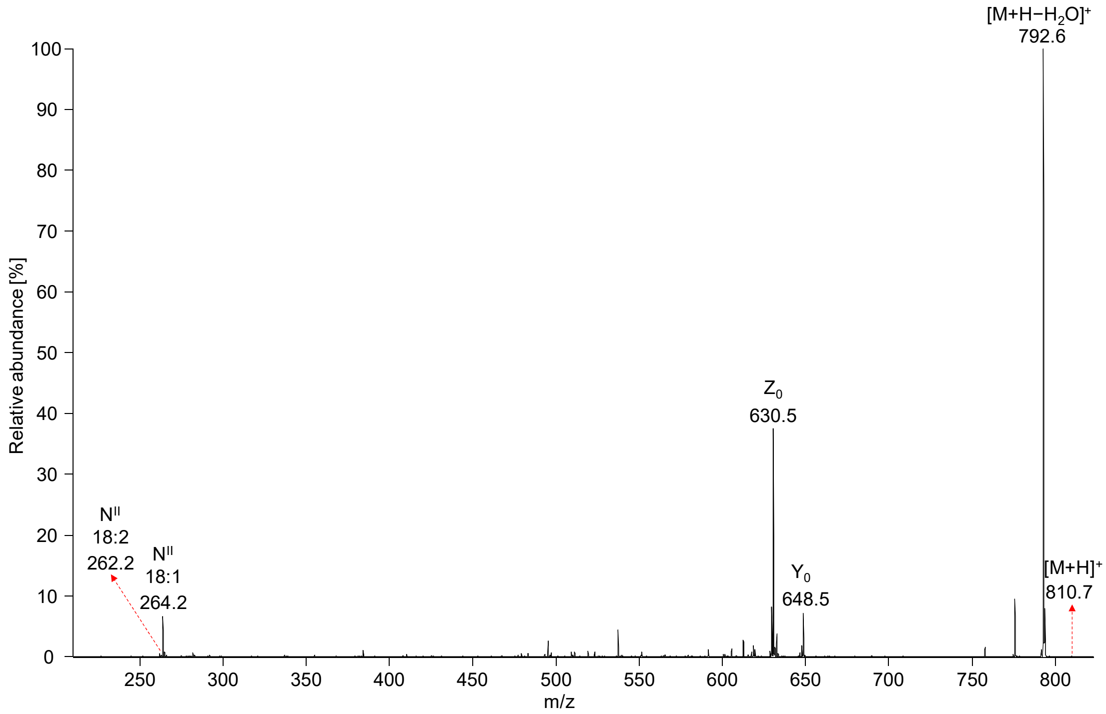

Figure S14. MS/MS spectrum of HexCer 42:2 at *m/z* 810.7 with characteristic fragments.

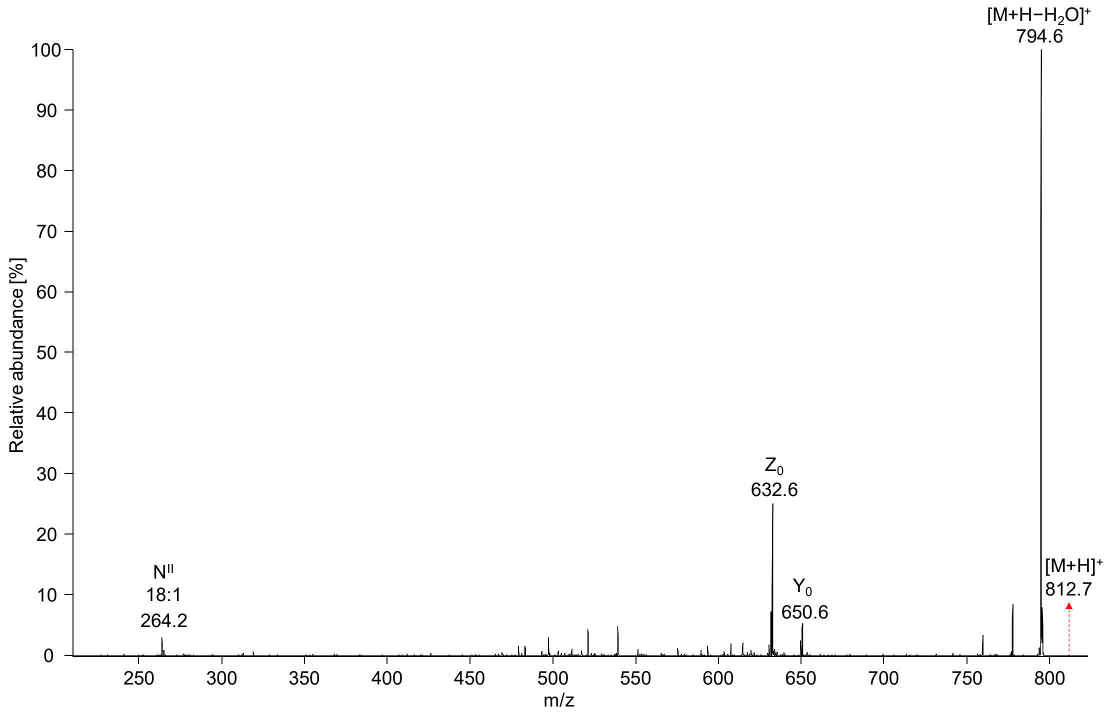

Figure S15. MS/MS spectrum of HexCer 42:1 at *m/z* 812.7 with characteristic fragments.

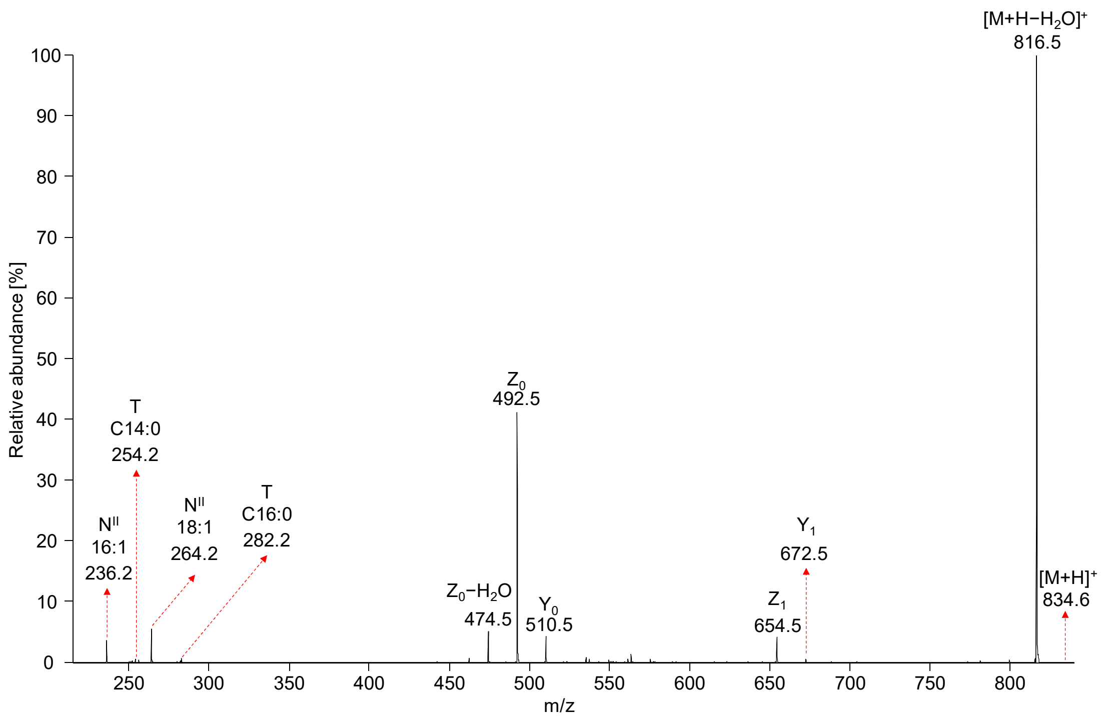

Figure S16. MS/MS spectrum of Hex2Cer 32:1 at *m/z* 834.6 with characteristic fragments.

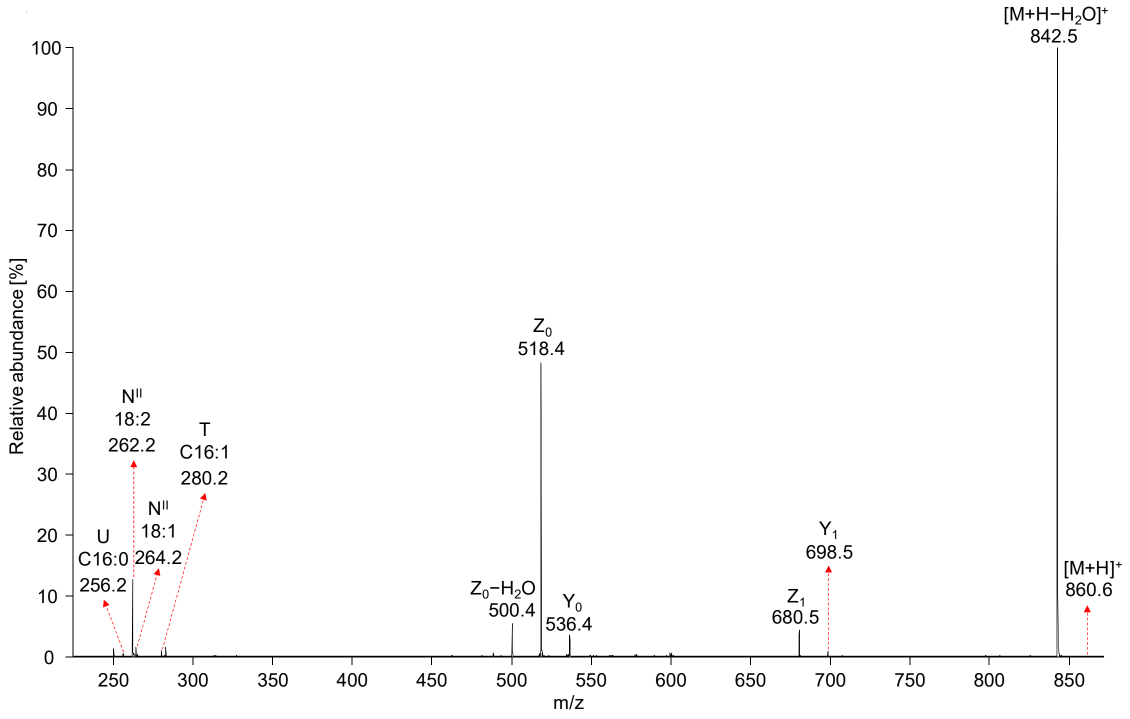

Figure S17. MS/MS spectrum of Hex2Cer 34:2 at *m/z* 860.6 with characteristic fragments.

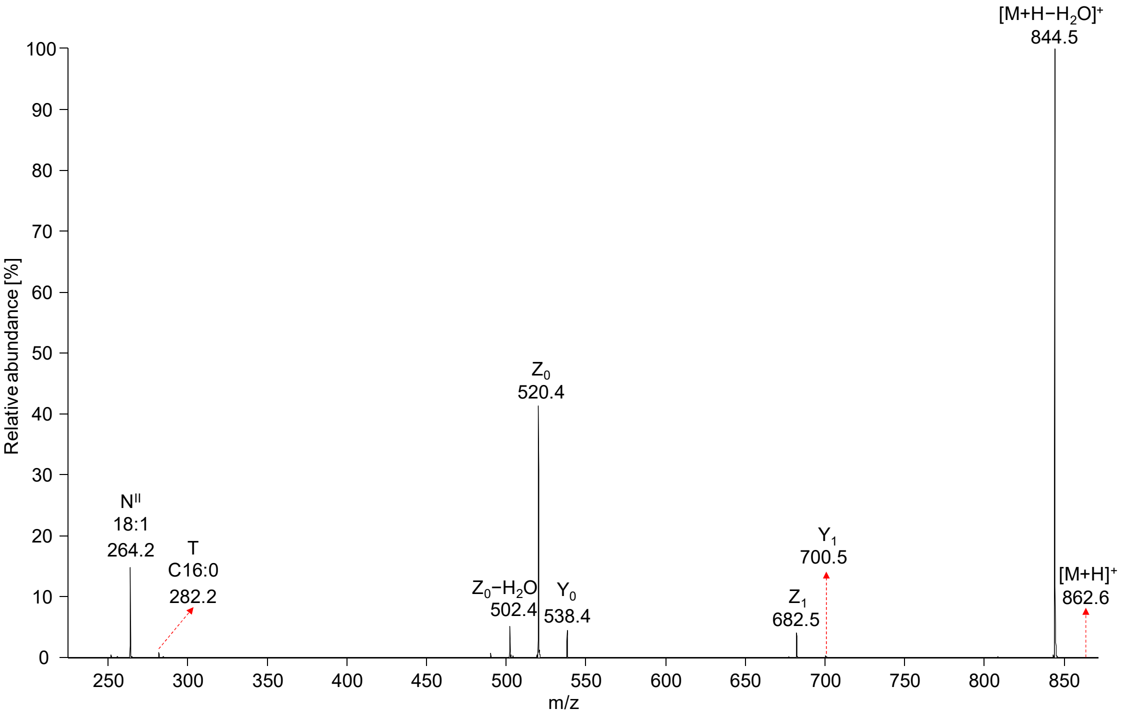

Figure S18. MS/MS spectrum of Hex2Cer 34:1 at *m/z* 862.6 with characteristic fragments.

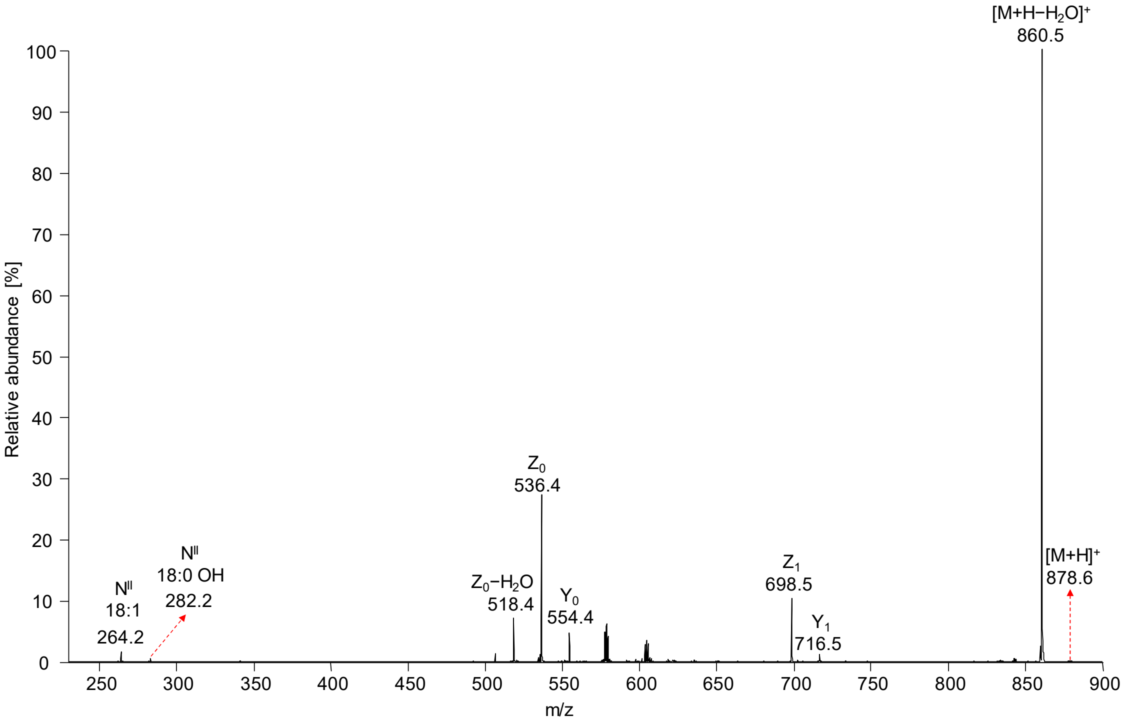

Figure S19. MS/MS spectrum of Hex2Cer 34:1 OH at *m/z* 878.6 with characteristic fragments.

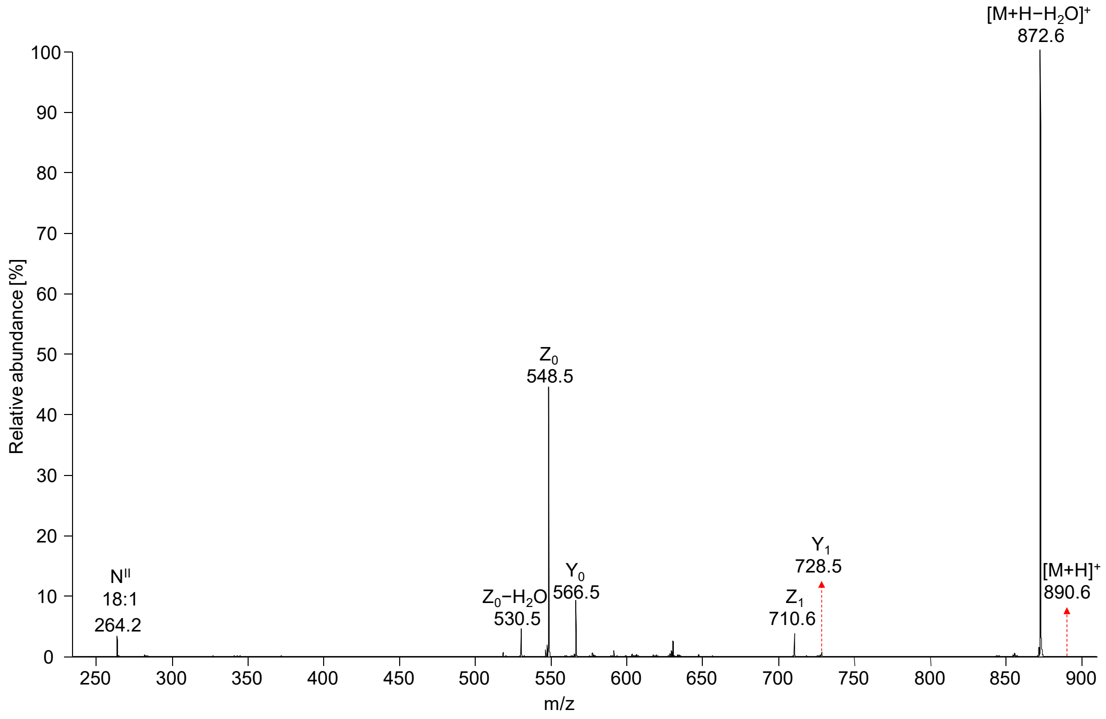

Figure S20. MS/MS spectrum of Hex2Cer 36:1 at *m/z* 890.6 with characteristic fragments.

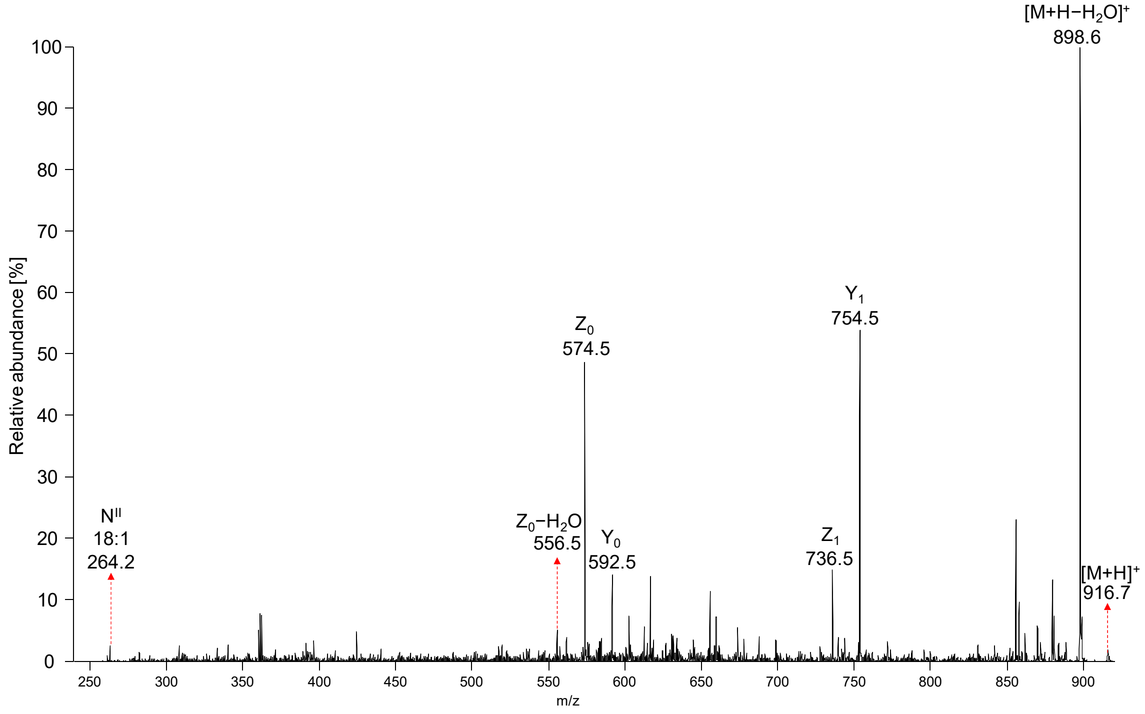

Figure S21. MS/MS spectrum of Hex2Cer 38:2 at *m/z* 916.7 with characteristic fragments.

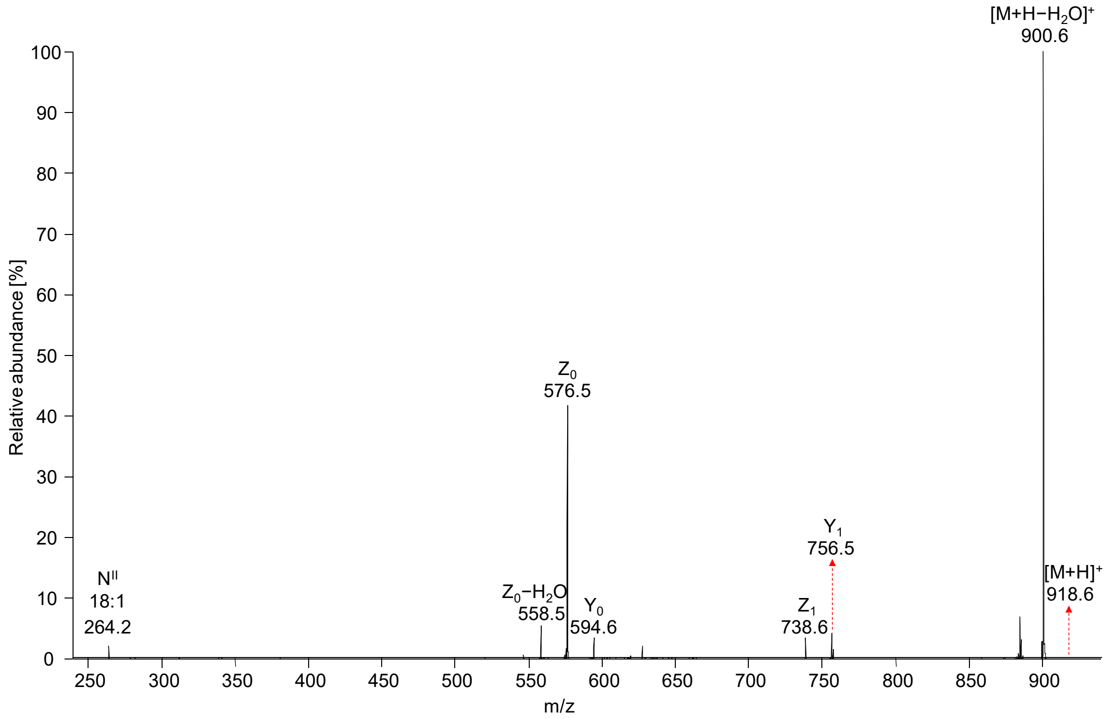

Figure S22. MS/MS spectrum of Hex2Cer 38:1 at *m/z* 918.6 with characteristic fragments.

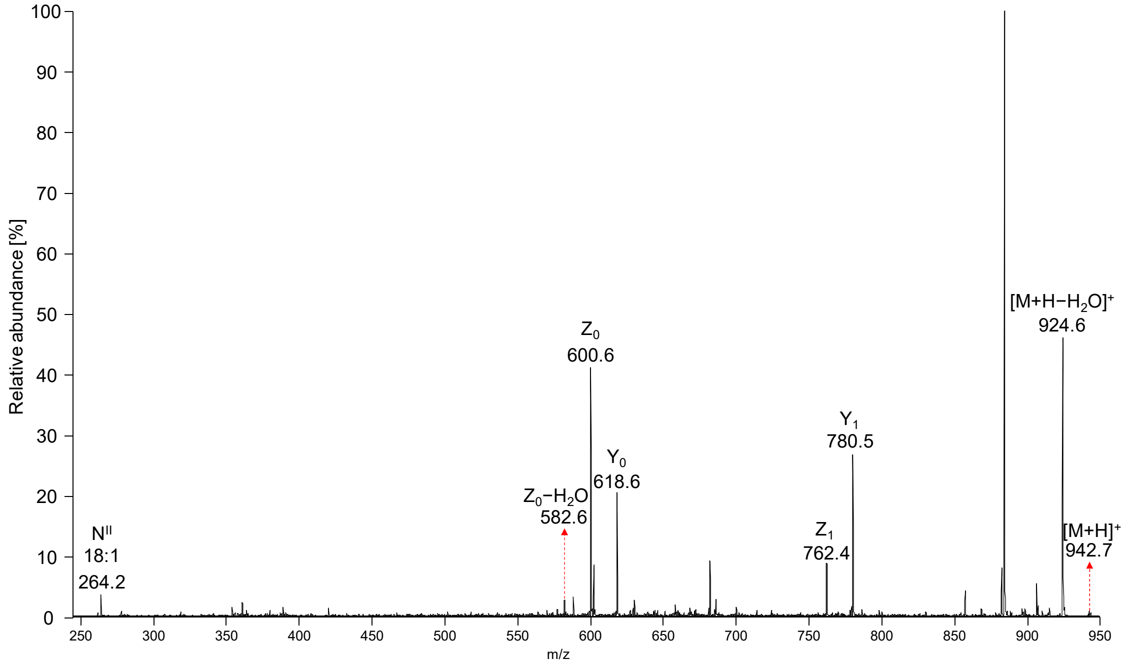

Figure S23. MS/MS spectrum of Hex2Cer 40:3 at *m/z* 942.7 with characteristic fragments.

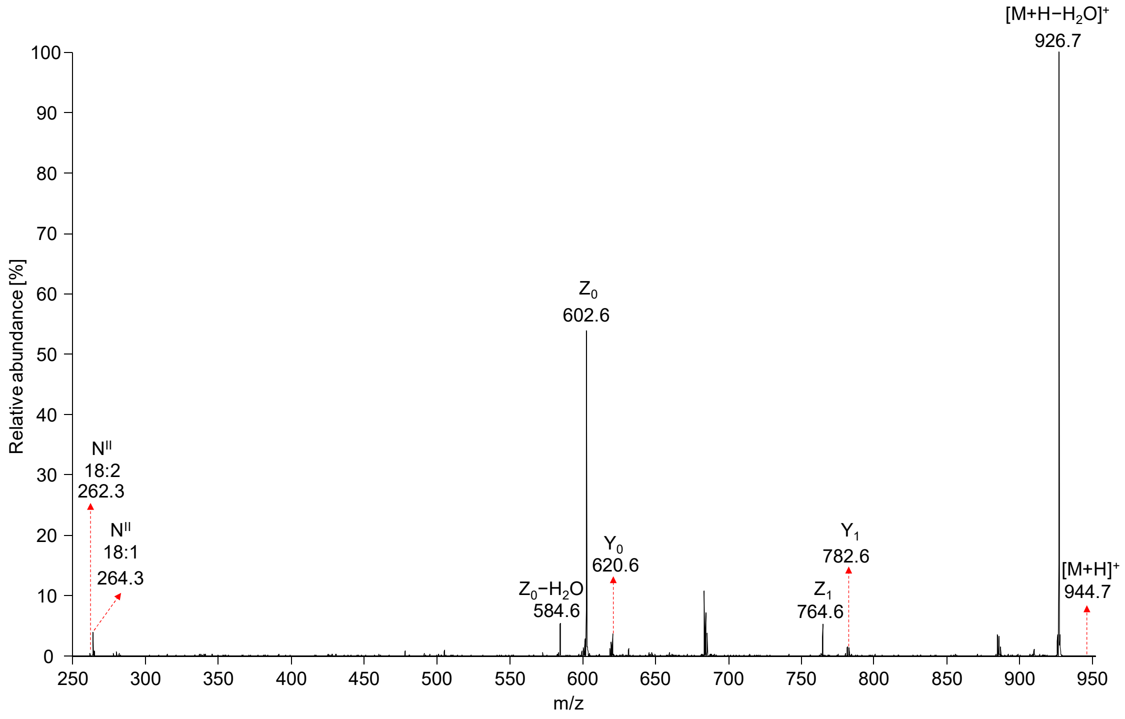

Figure S24. MS/MS spectrum of Hex2Cer 40:2 at *m/z* 944.7 with characteristic fragments.

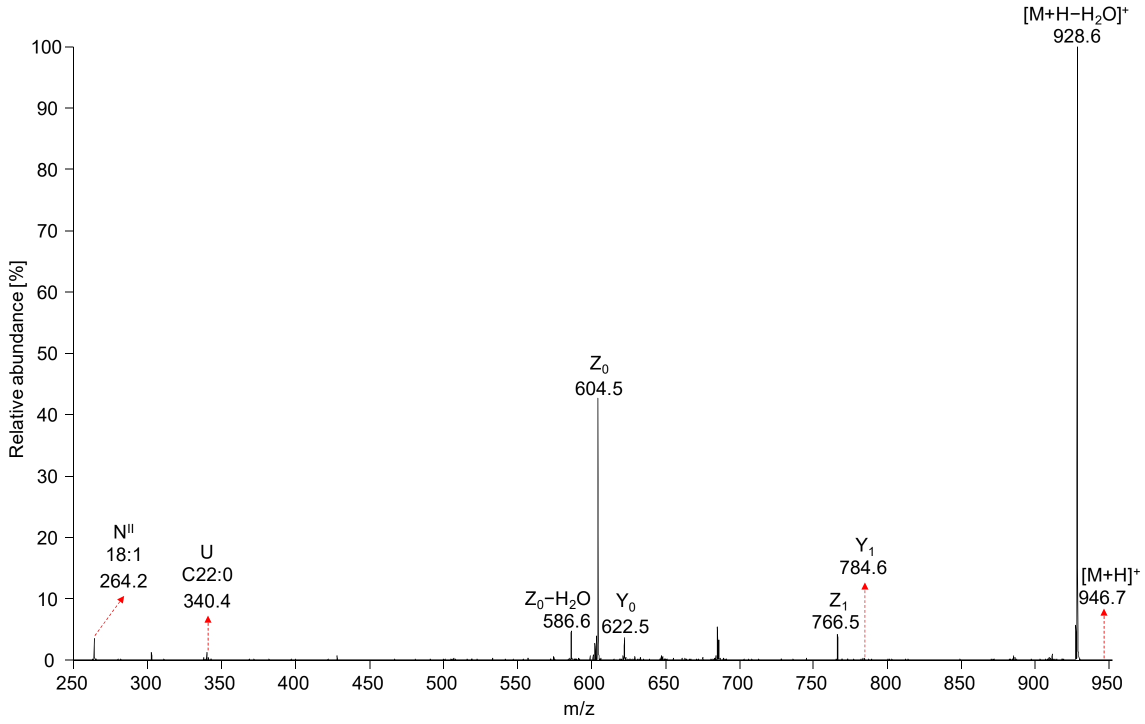

Figure S25. MS/MS spectrum of Hex2Cer 40:1 at *m/z* 946.7 with characteristic fragments.

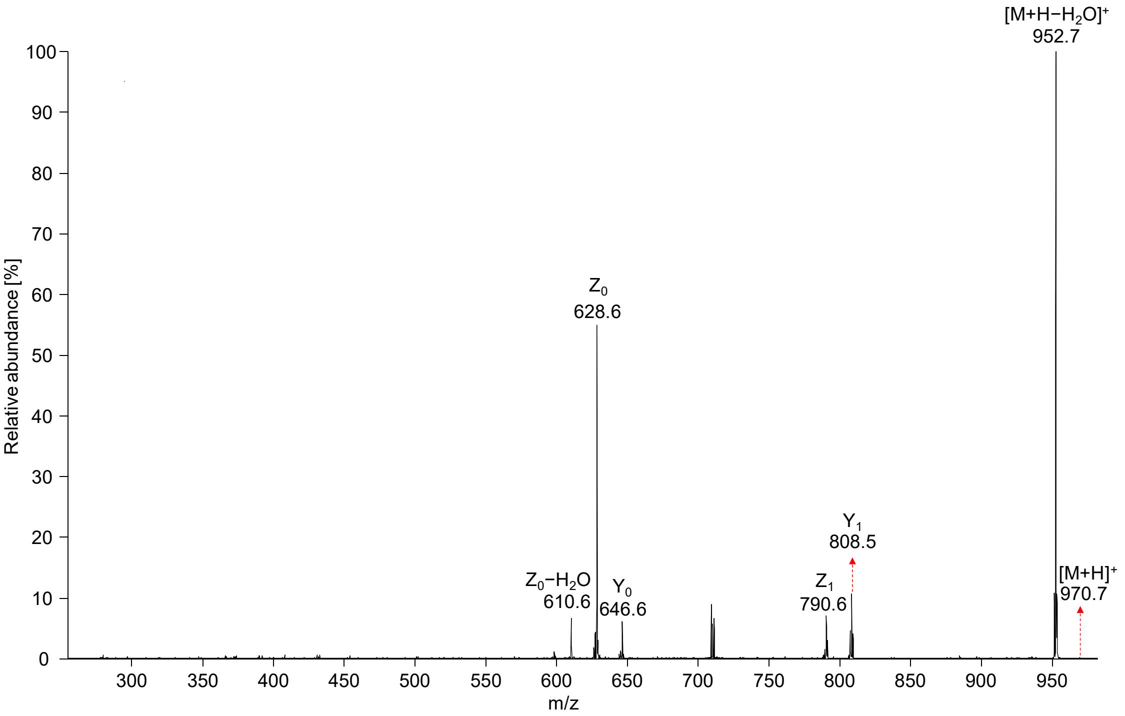

Figure S26. MS/MS spectrum of Hex2Cer 42:3 at *m/z* 970.7 with characteristic fragments.

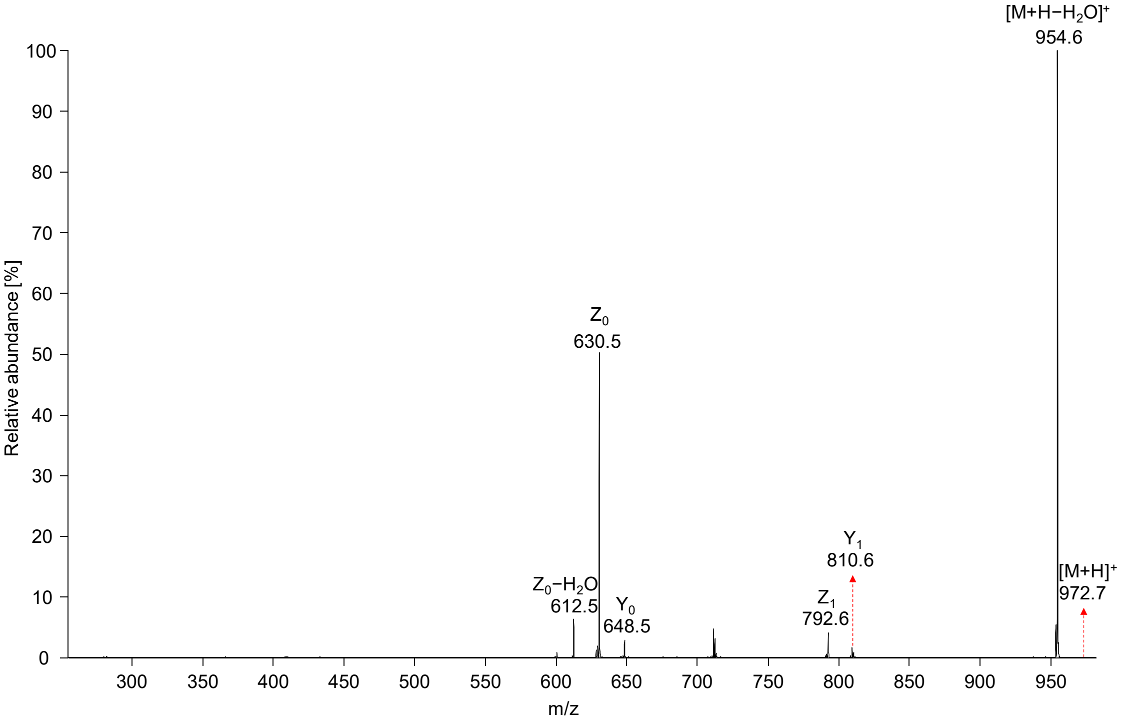

Figure S27. MS/MS spectrum of Hex2Cer 42:2 at *m/z* 972.7 with characteristic fragments.

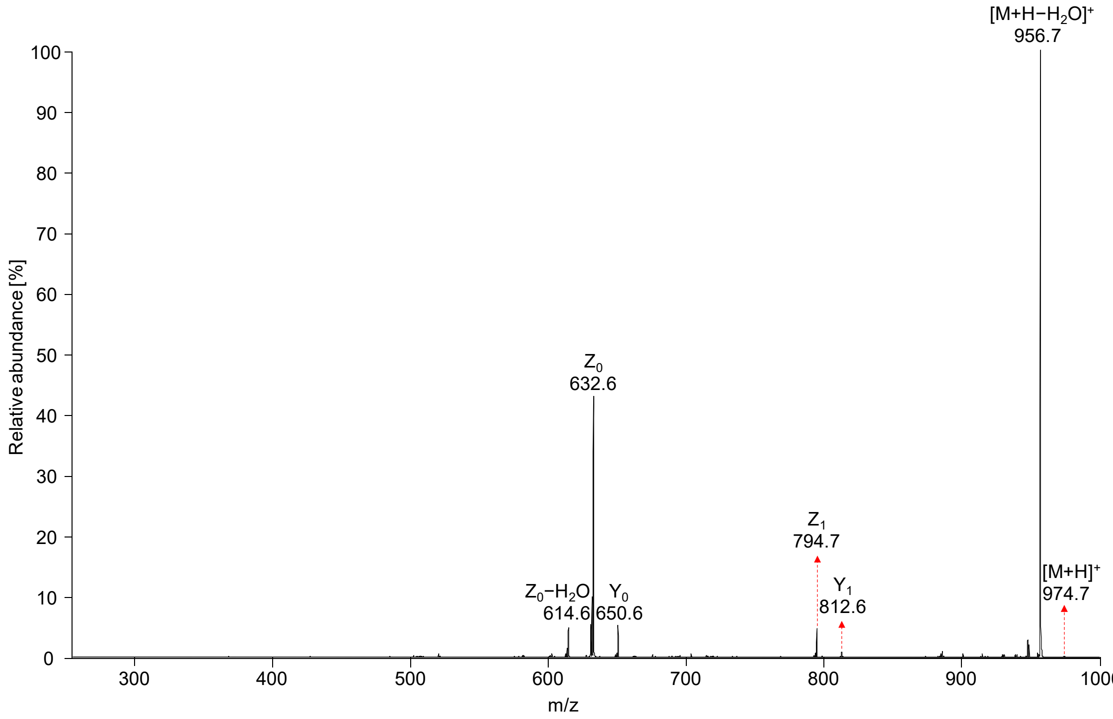

Figure S28. MS/MS spectrum of Hex2Cer 42:1 at *m/z* 974.7 with characteristic fragments.

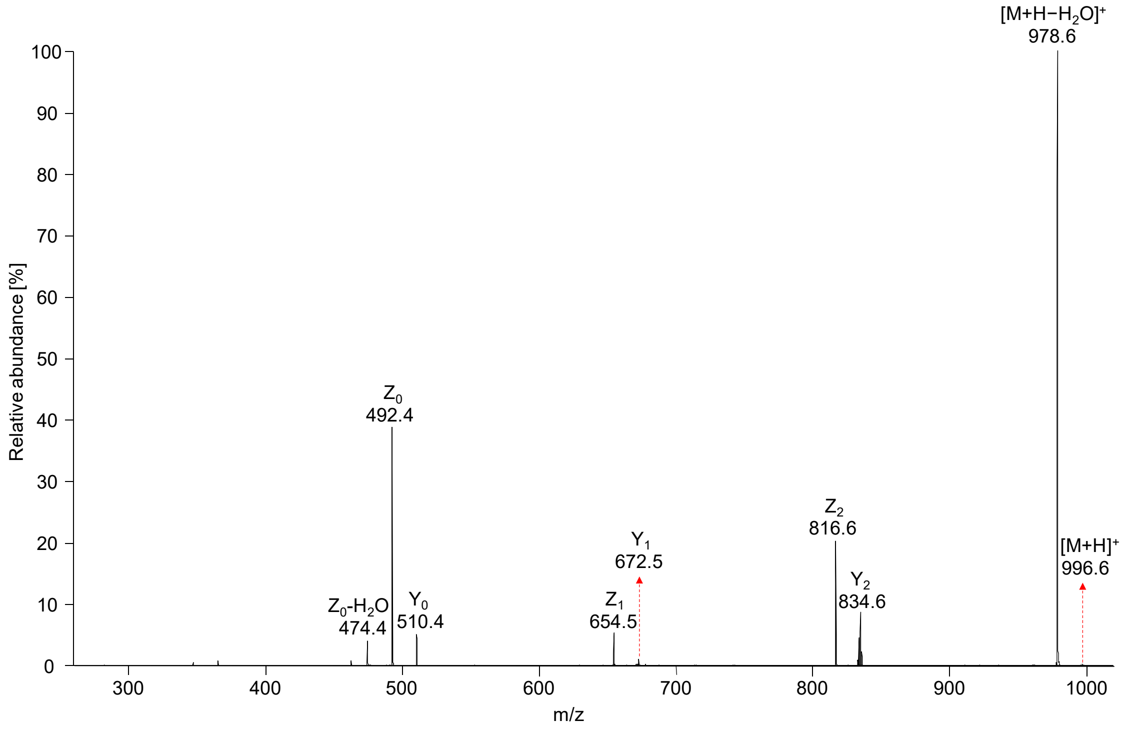

Figure S29. MS/MS spectrum of Gb3 32:1 at *m/z* 996.6 with characteristic fragments.

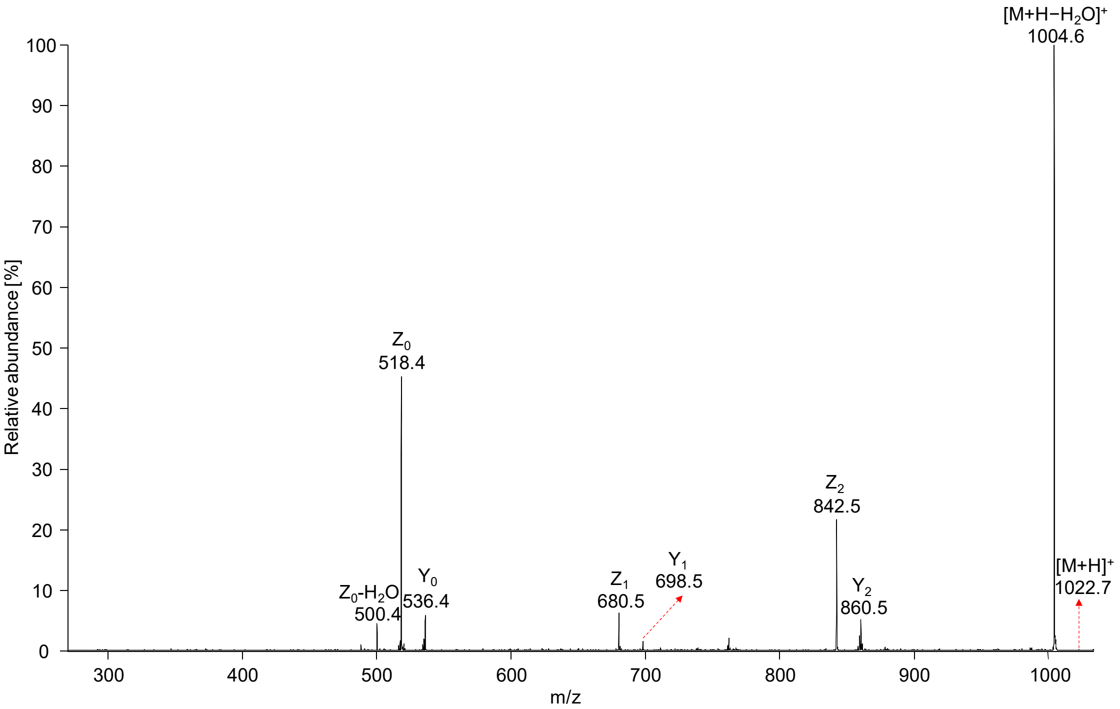

Figure S30. MS/MS spectrum of Gb3 34:2 at *m/z* 1022.7 with characteristic fragments.

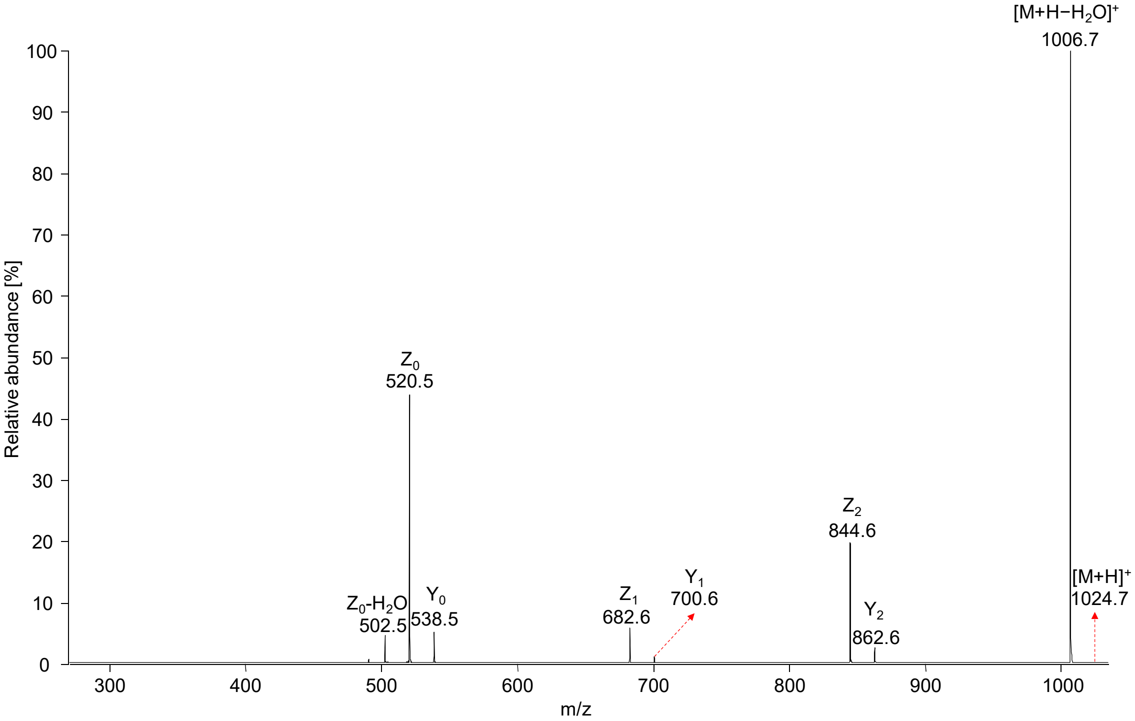

Figure S31. MS/MS spectrum of Gb3 34:1 at *m/z* 1024.7 with characteristic fragments.

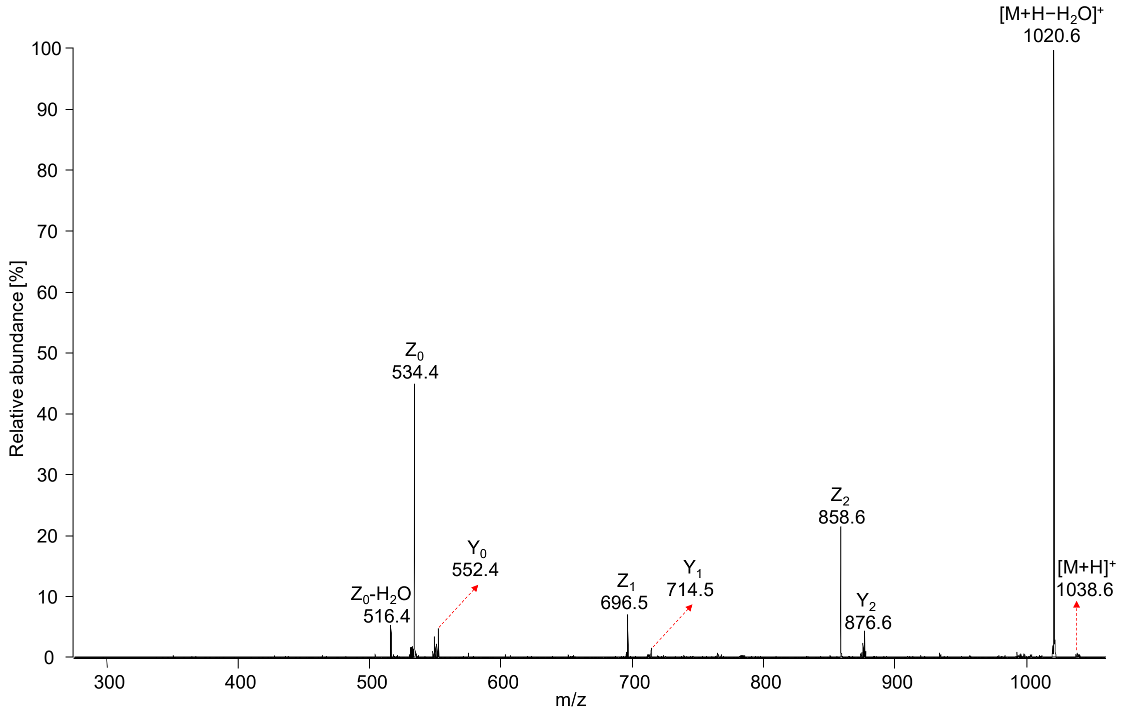

Figure S32. MS/MS spectrum of Gb3 34:2 OH and/or 35:1 at *m/z* 1038.6 with characteristic fragments.

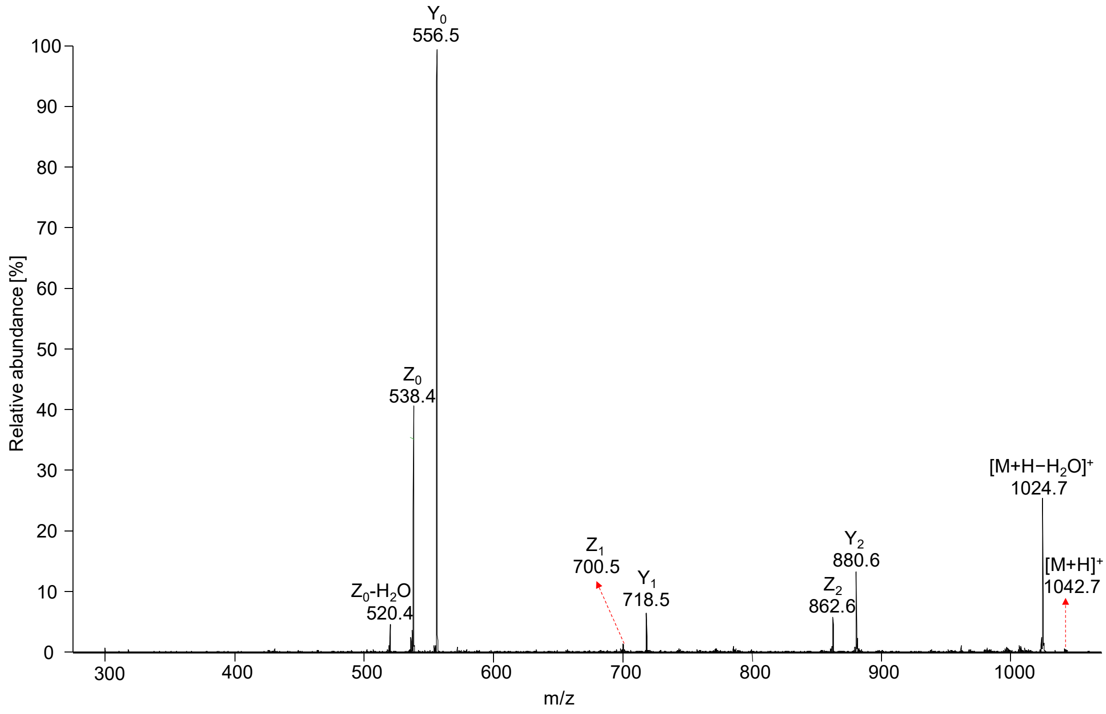

Figure S33. MS/MS spectrum of Gb3 34:0 OH at *m/z* 1042.7 with characteristic fragments.

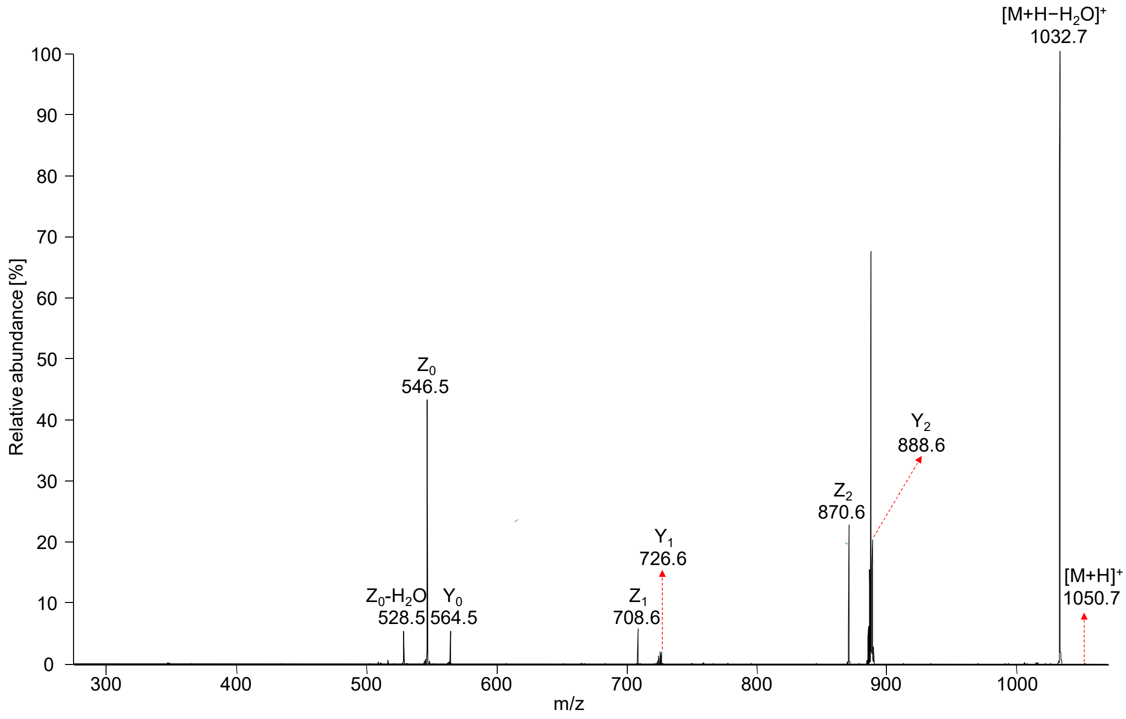

Figure S34. MS/MS spectrum of Gb3 36:2 at *m/z* 1050.7 with characteristic fragments.

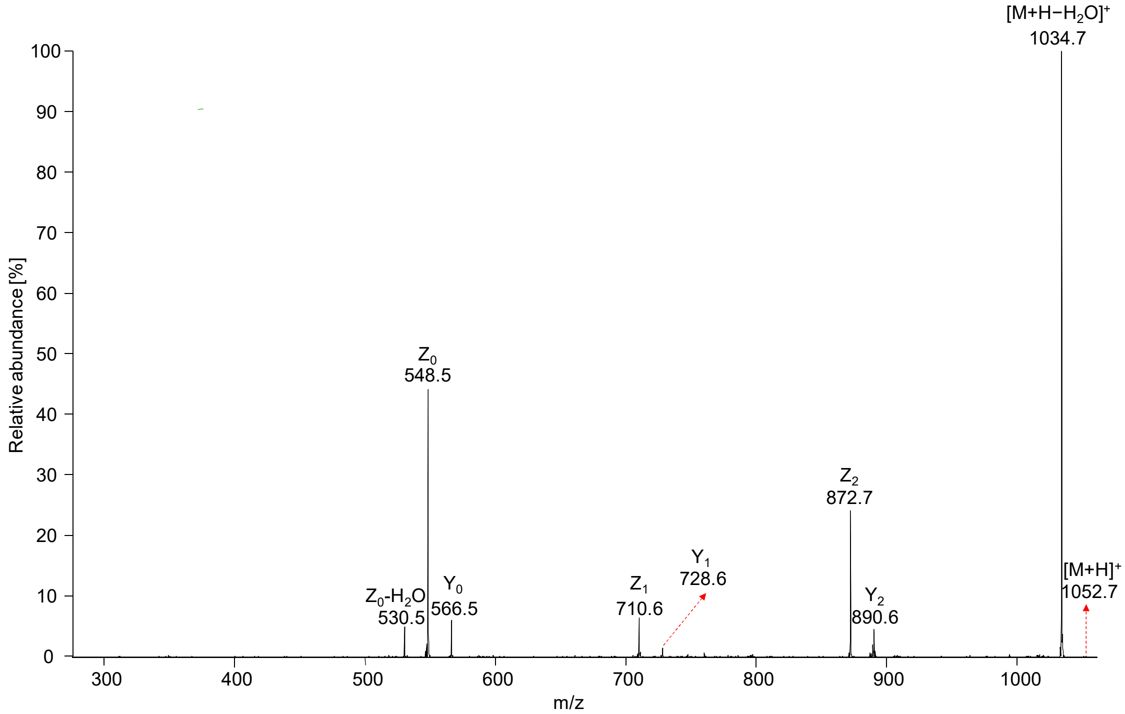

Figure S35. MS/MS spectrum of Gb3 36:1 at *m/z* 1052.7 with characteristic fragments.

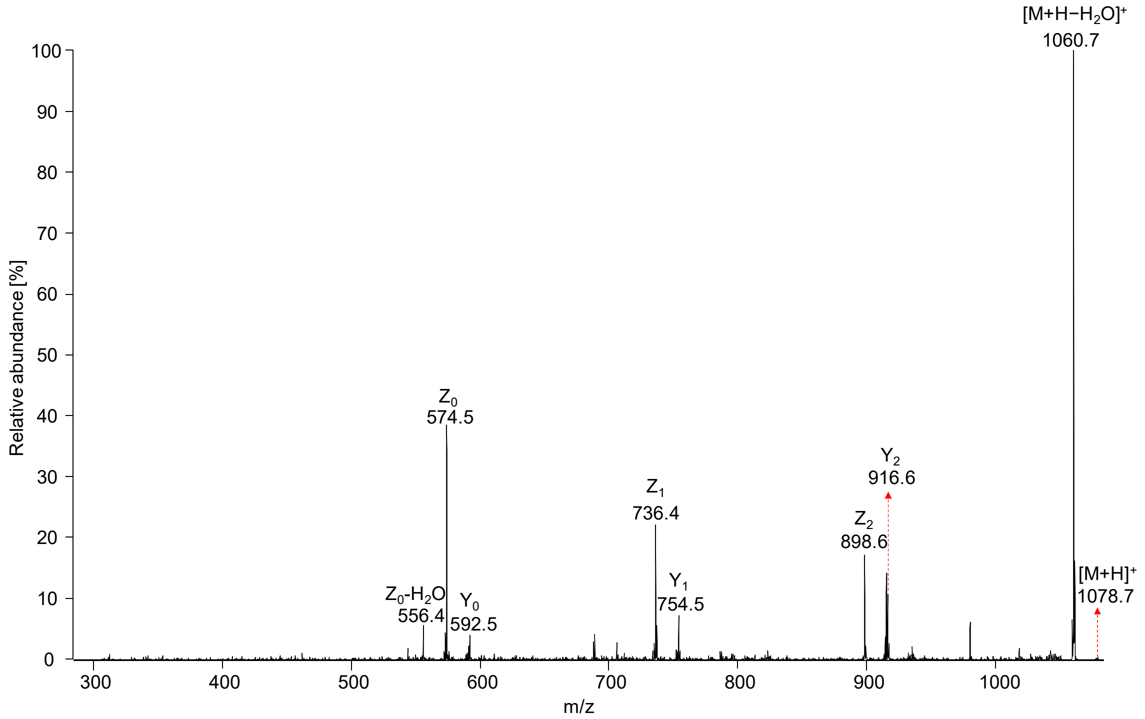

Figure S36. MS/MS spectrum of Gb3 38:2 at *m/z* 1078.7 with characteristic fragments.

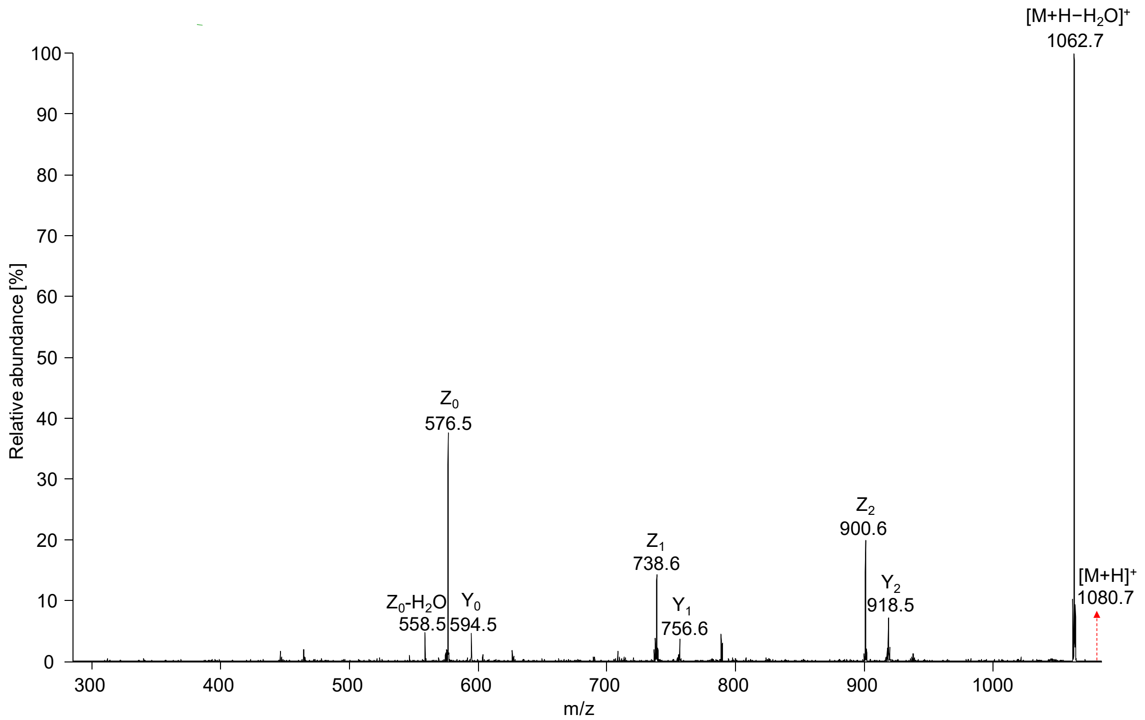

Figure S37. MS/MS spectrum of Gb3 38:1 at *m/z* 1080.7 with characteristic fragments.

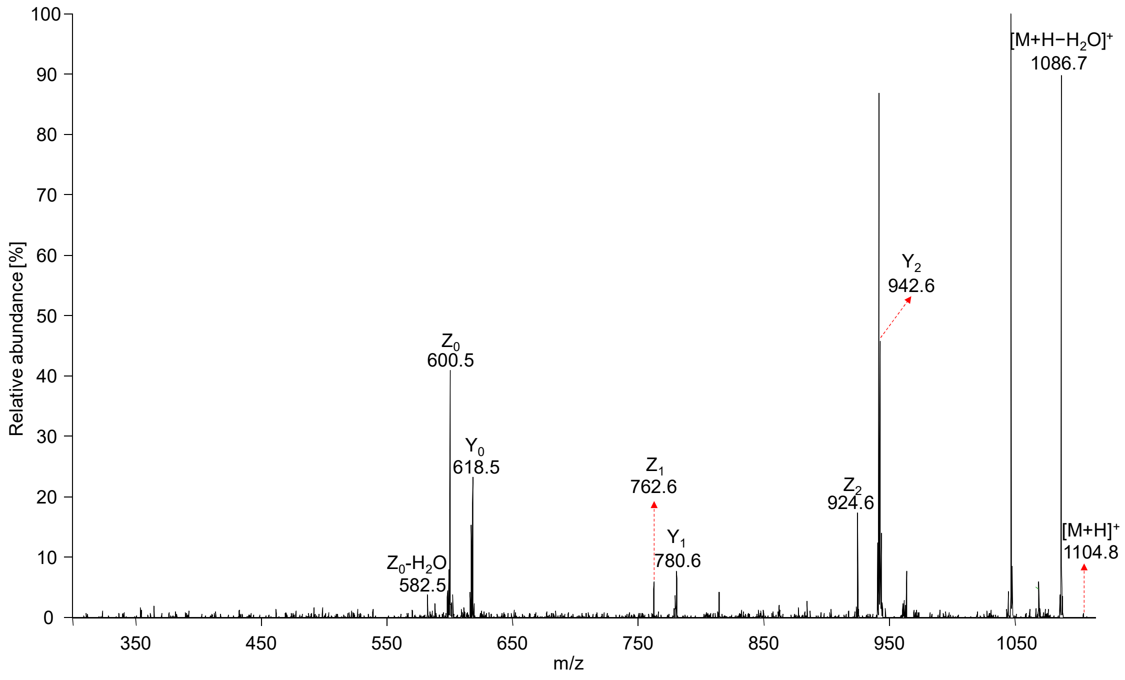

Figure S38. MS/MS spectrum of Gb3 40:3 at *m/z* 1104.8 with characteristic fragments.

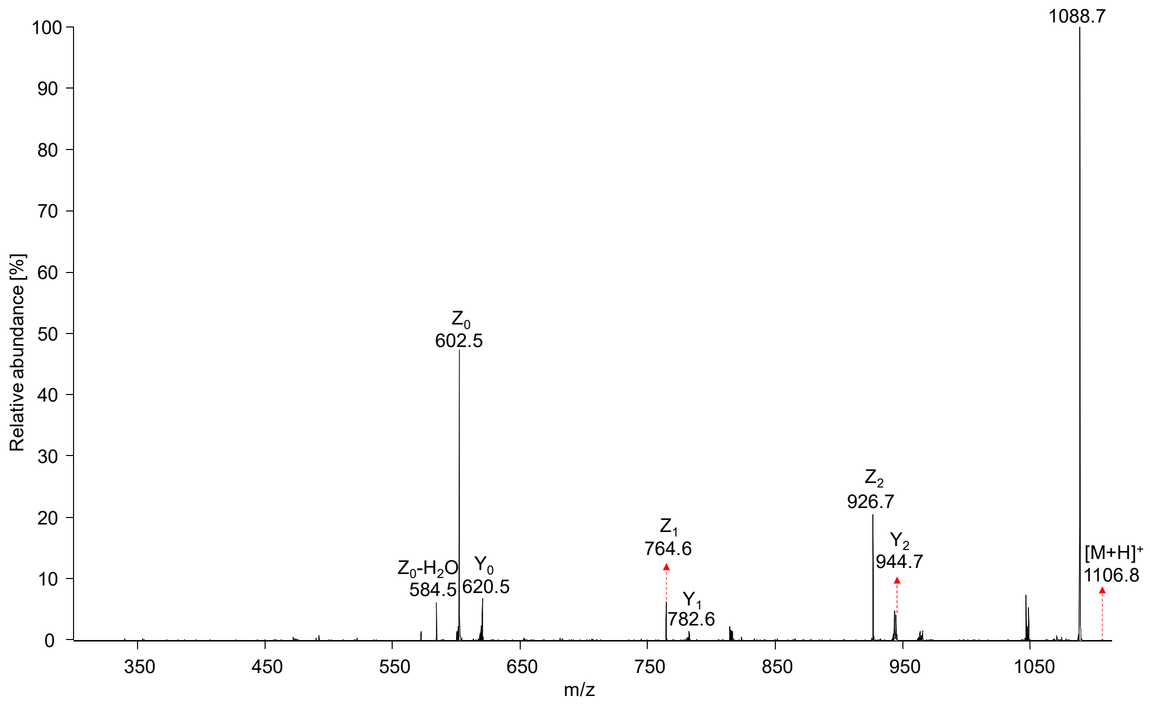

Figure S39. MS/MS spectrum of Gb3 40:2 at *m/z* 1106.8 with characteristic fragments.

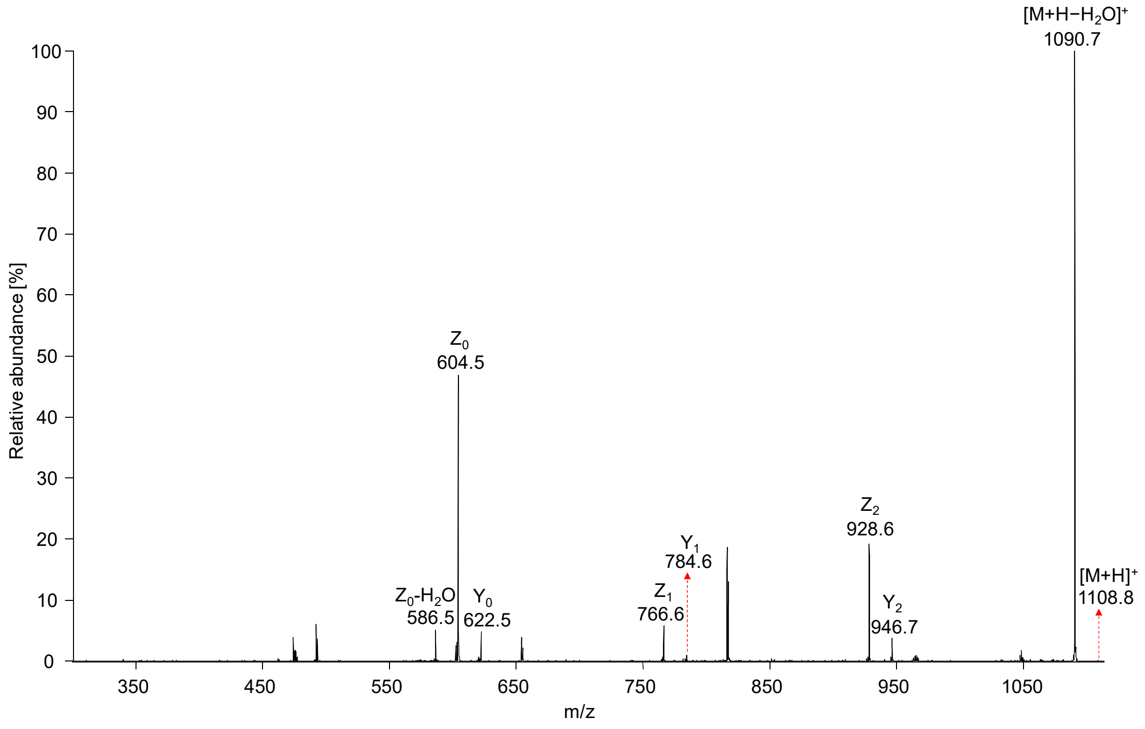

Figure S40. MS/MS spectrum of Gb3 40:1 at *m/z* 1108.8 with characteristic fragments.

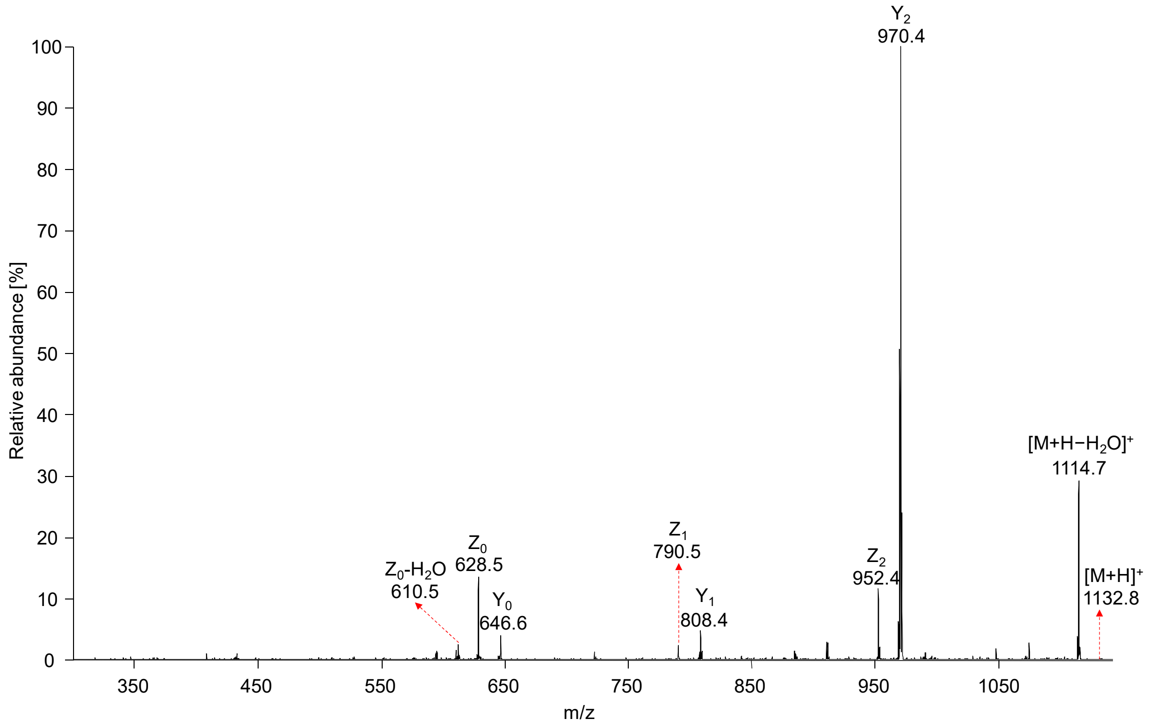

Figure S41. MS/MS spectrum of Gb3 42:3 at *m/z* 1132.8 with characteristic fragments.

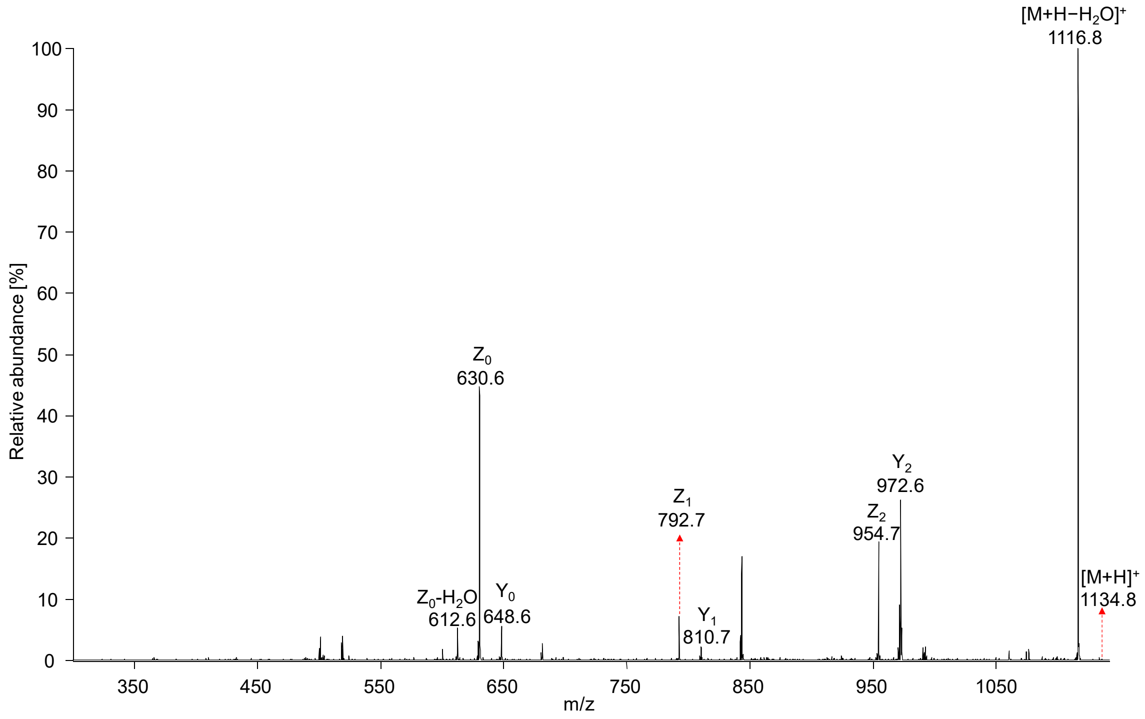

Figure S42. MS/MS spectrum of Gb3 42:2 at *m/z* 1134.8 with characteristic fragments.

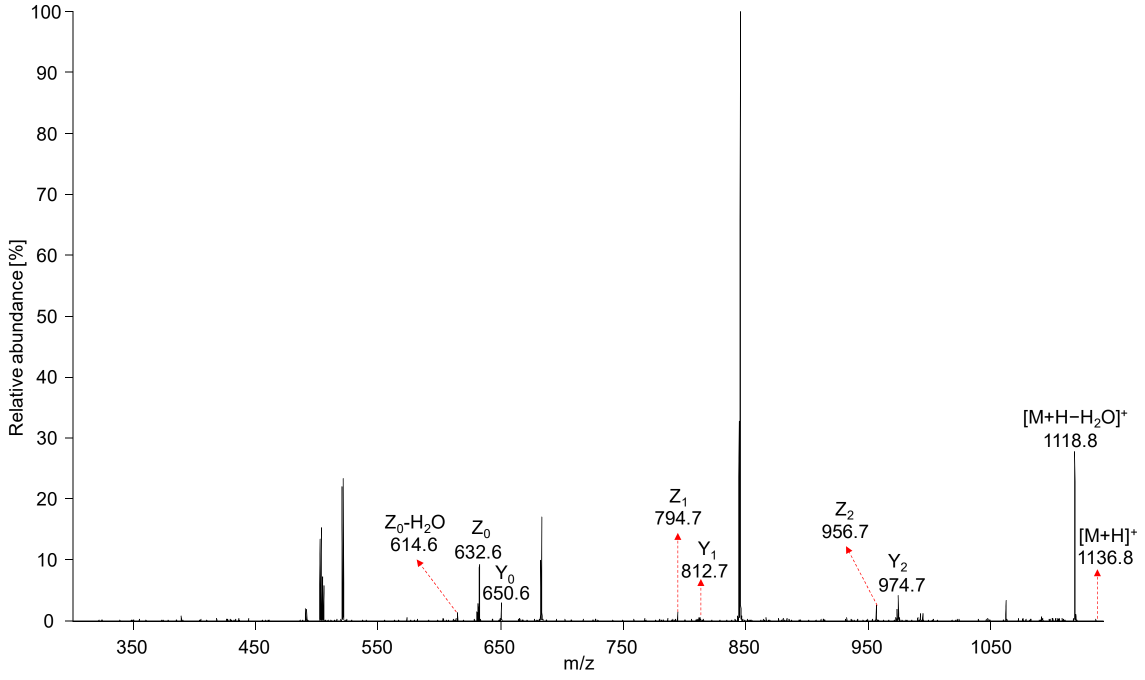

Figure S43. MS/MS spectrum of Gb3 42:1 at *m/z* 1136.8 with characteristic fragments.

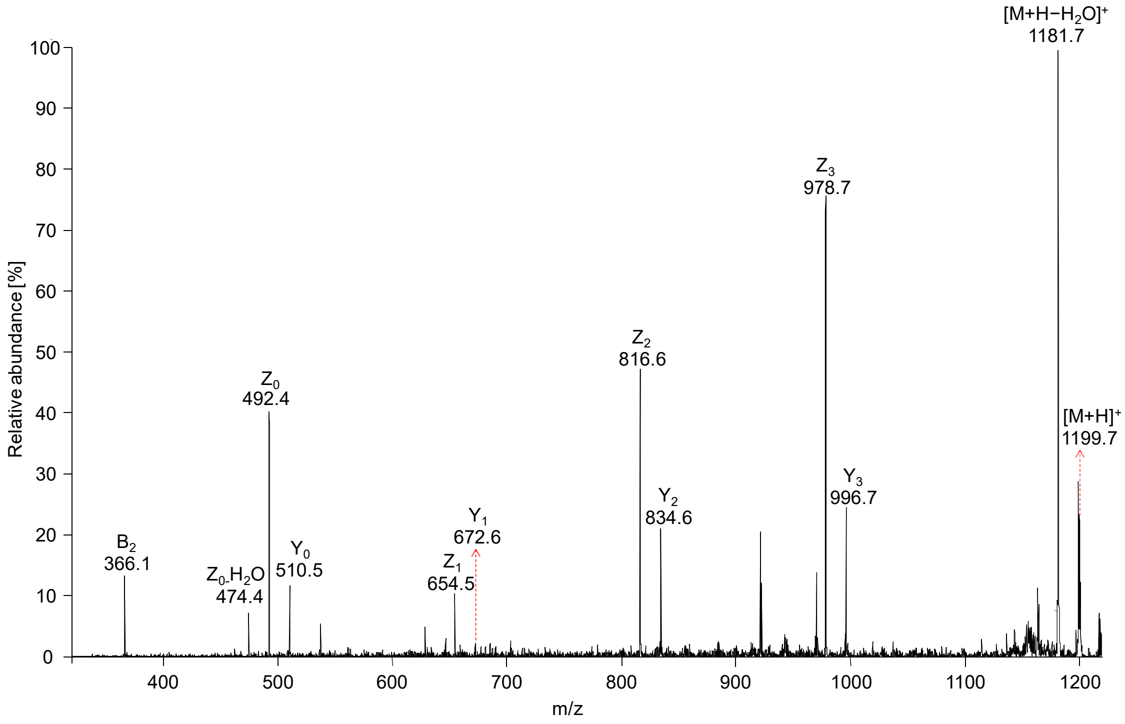

Figure S44. MS/MS spectrum of Gb4 32:1 at *m/z* 1199.7 with characteristic fragments.

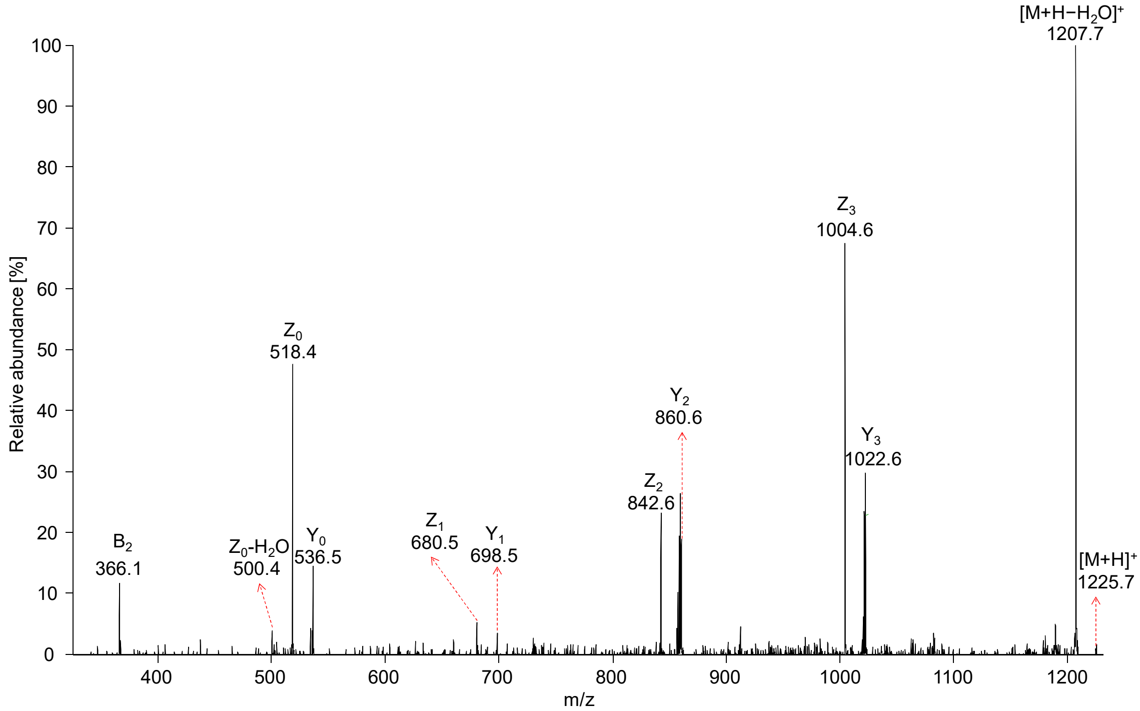

Figure S45. MS/MS spectrum of Gb4 34:2 at *m/z* 1225.7 with characteristic fragments.

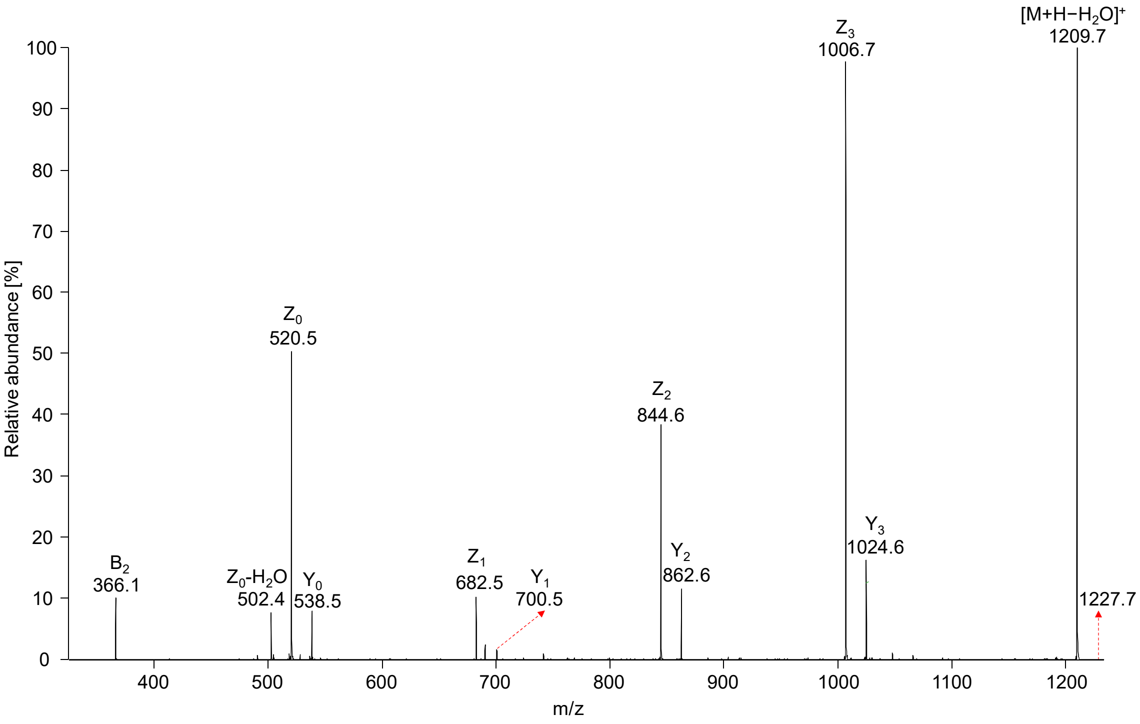

Figure S46. MS/MS spectrum of Gb4 34:1 at *m/z* 1227.7 with characteristic fragments.

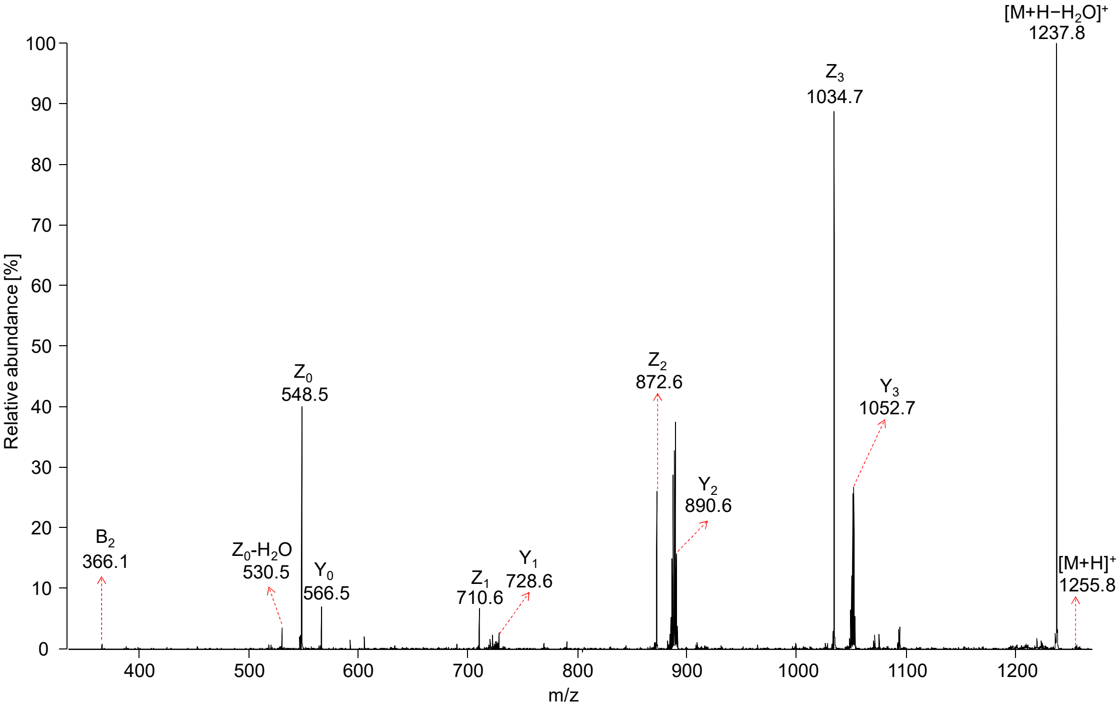

Figure S47. MS/MS spectrum of Gb4 36:1 at *m/z* 1255.8 with characteristic fragments.

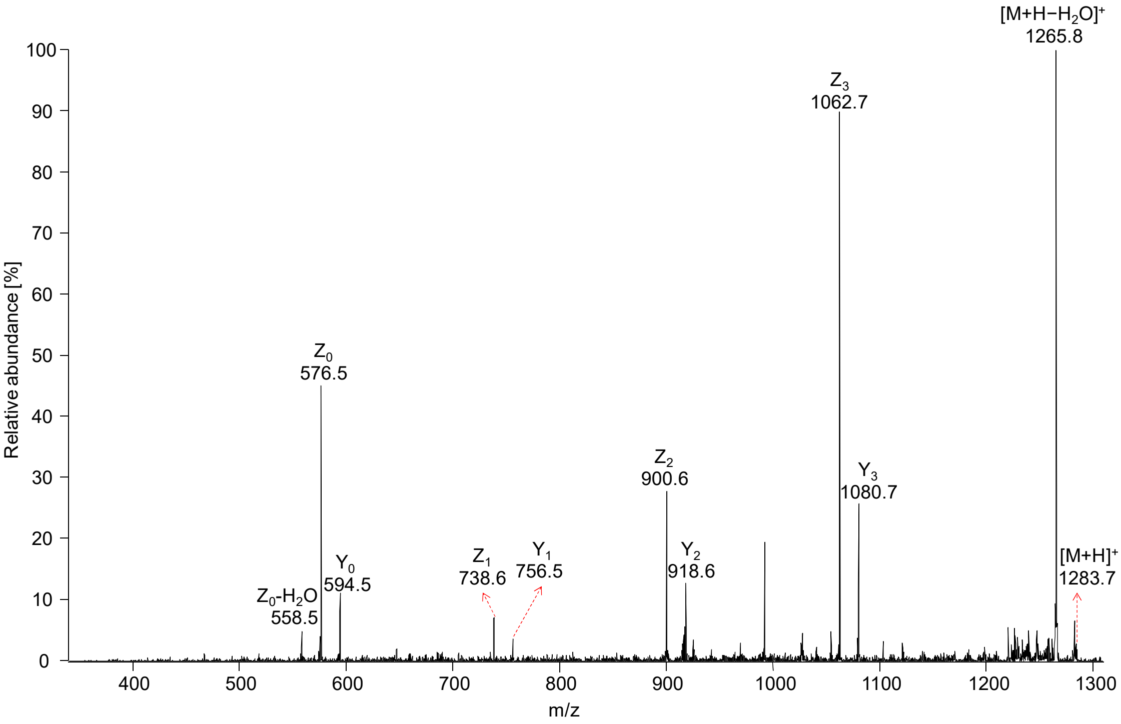

Figure S48. MS/MS spectrum of Gb4 38:1 at *m/z* 1283.7 with characteristic fragments.

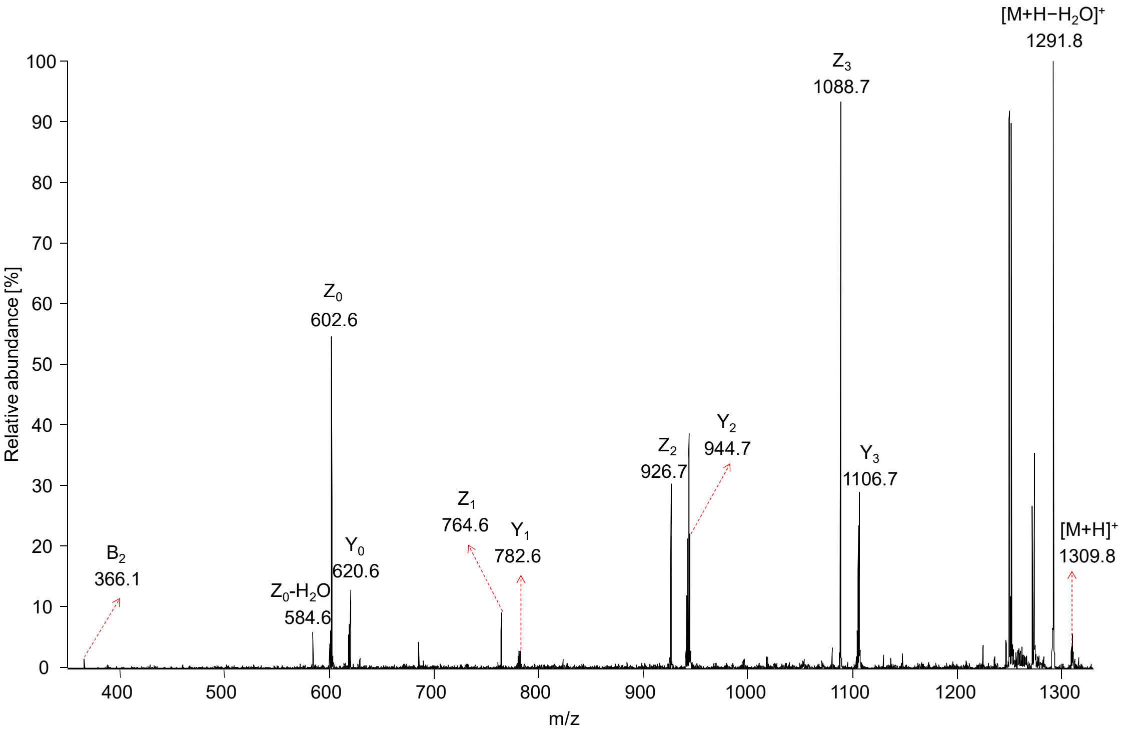

Figure S49. MS/MS spectrum of Gb4 40:2 at *m/z* 1309.8 with characteristic fragments.

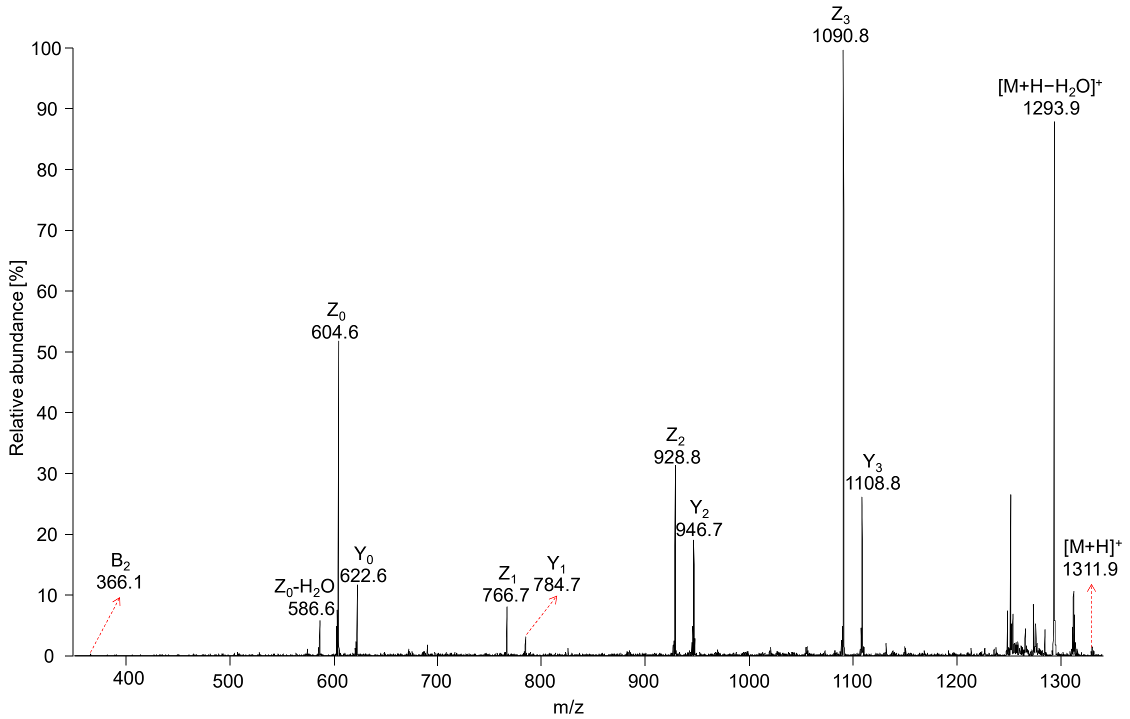

Figure S50. MS/MS spectrum of Gb4 40:1 at *m/z* 1311.9 with characteristic fragments.

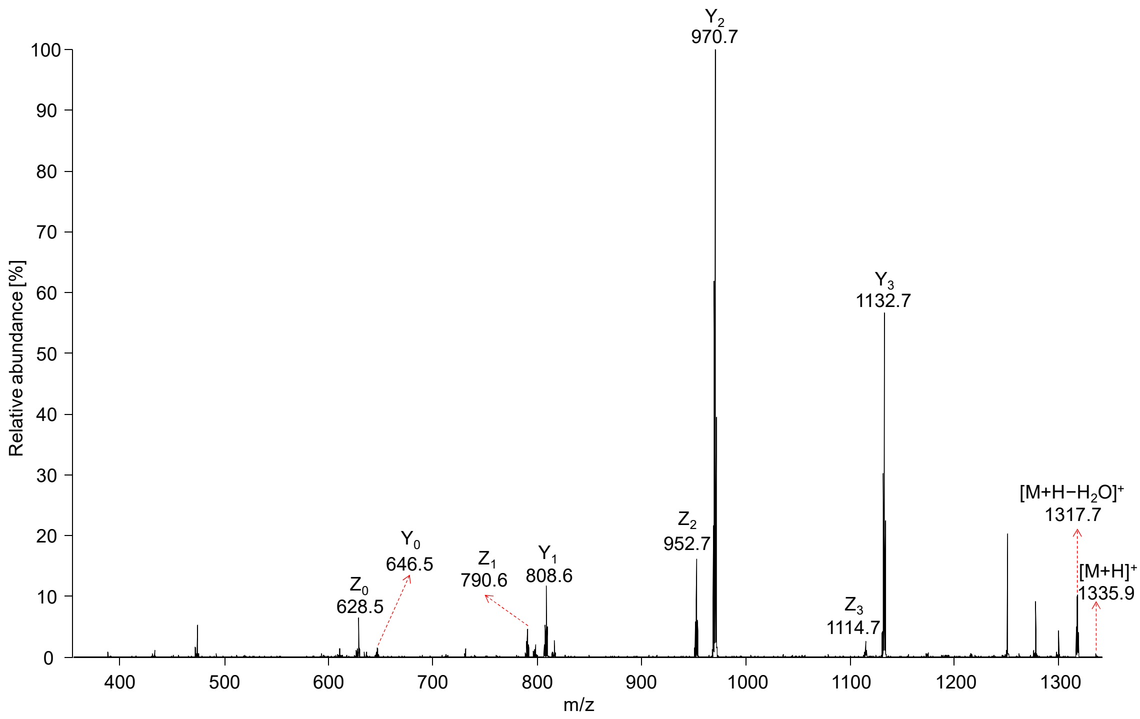

Figure S51. MS/MS spectrum of Gb4 42:3 at *m/z* 1335.9 with characteristic fragments.

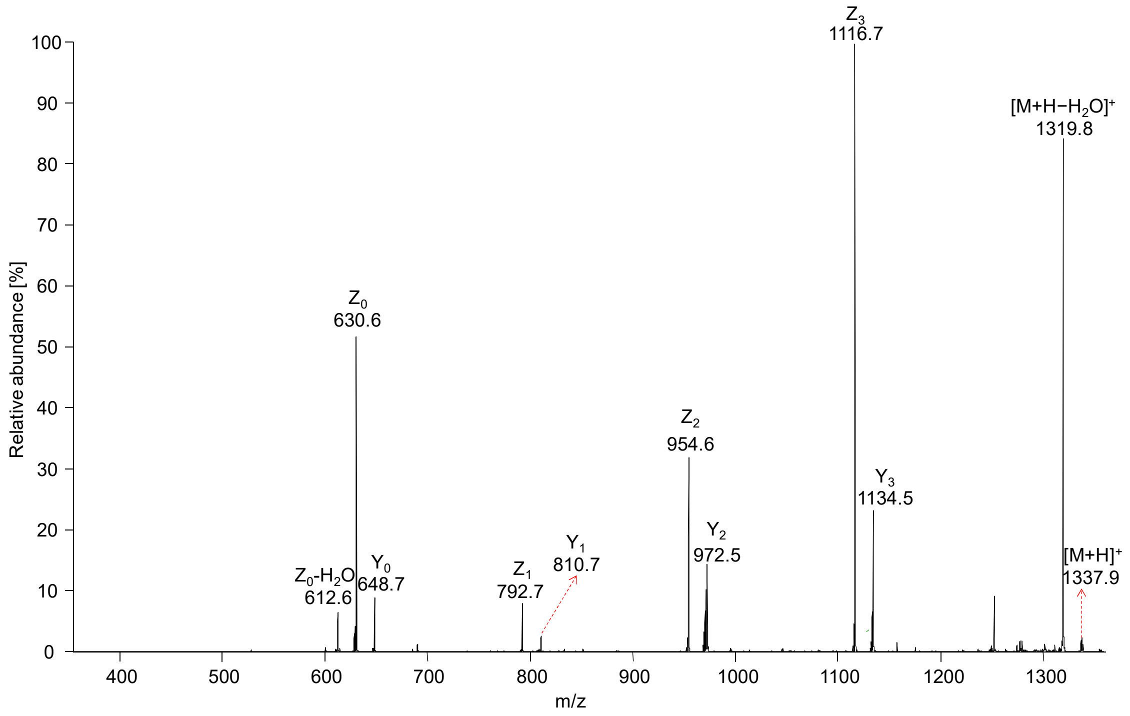

Figure S52. MS/MS spectrum of Gb_4 42:2 at *m/z* 1337.9 with characteristic fragments.

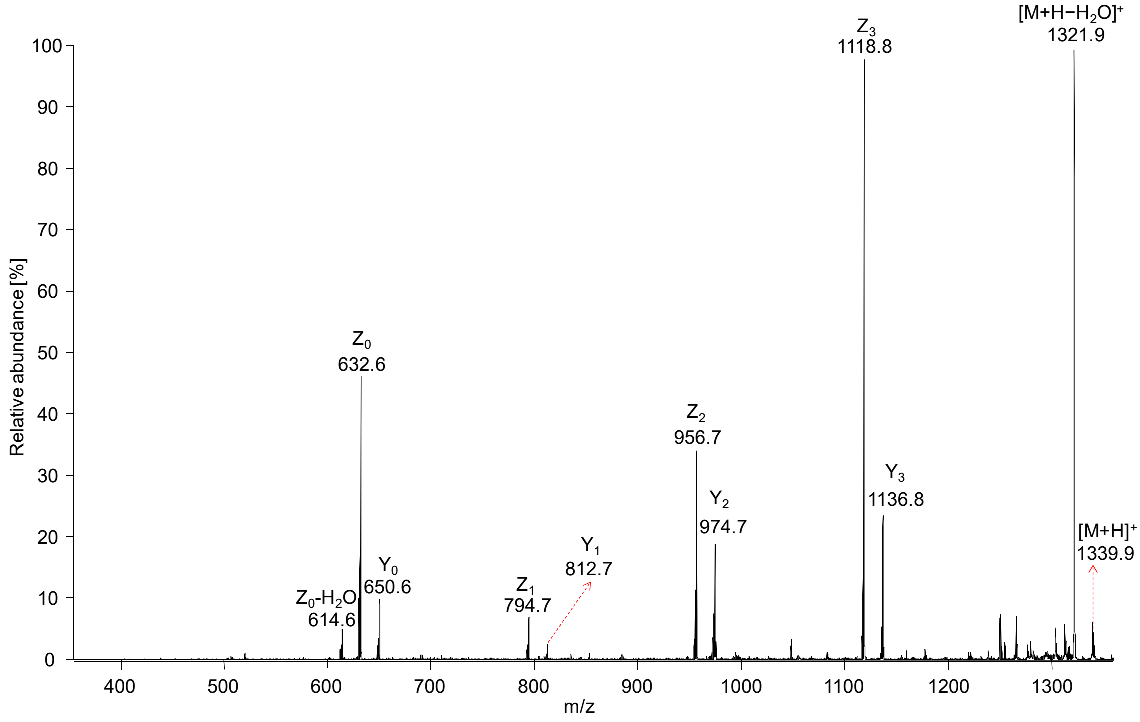

Figure S53. MS/MS spectrum of Gb_4 42:1 at *m/z* 1339.9 with characteristic fragments.

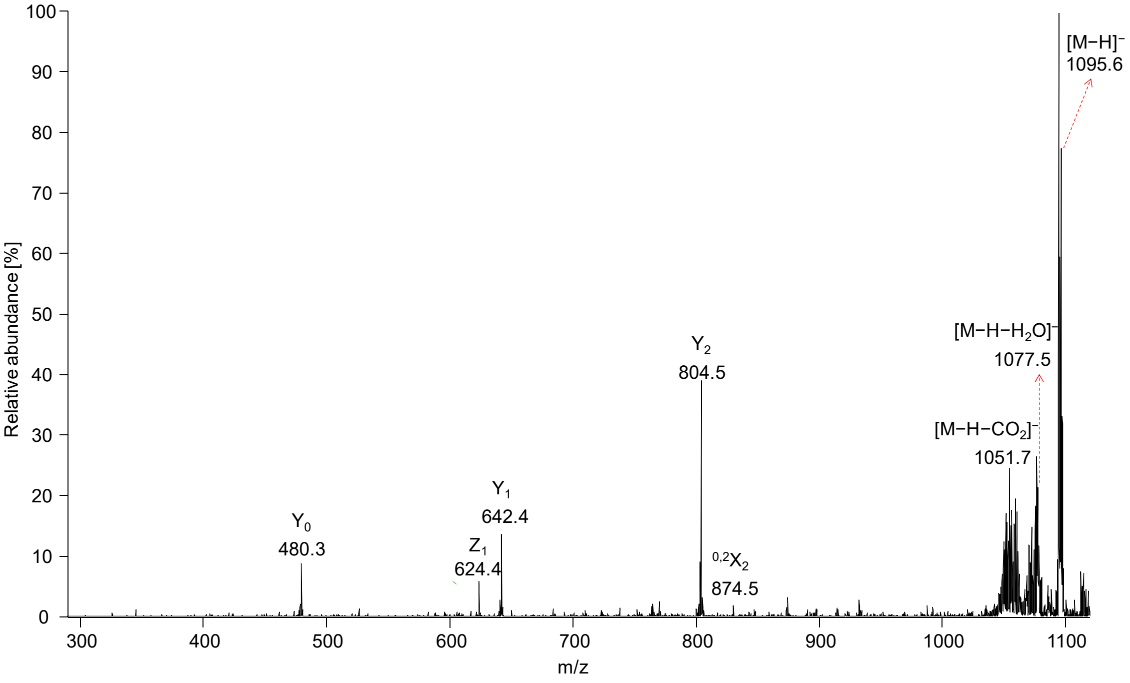

Figure S54. MS/MS spectrum of GM3 30:1 at *m/z* 1095.6 with characteristic fragments.

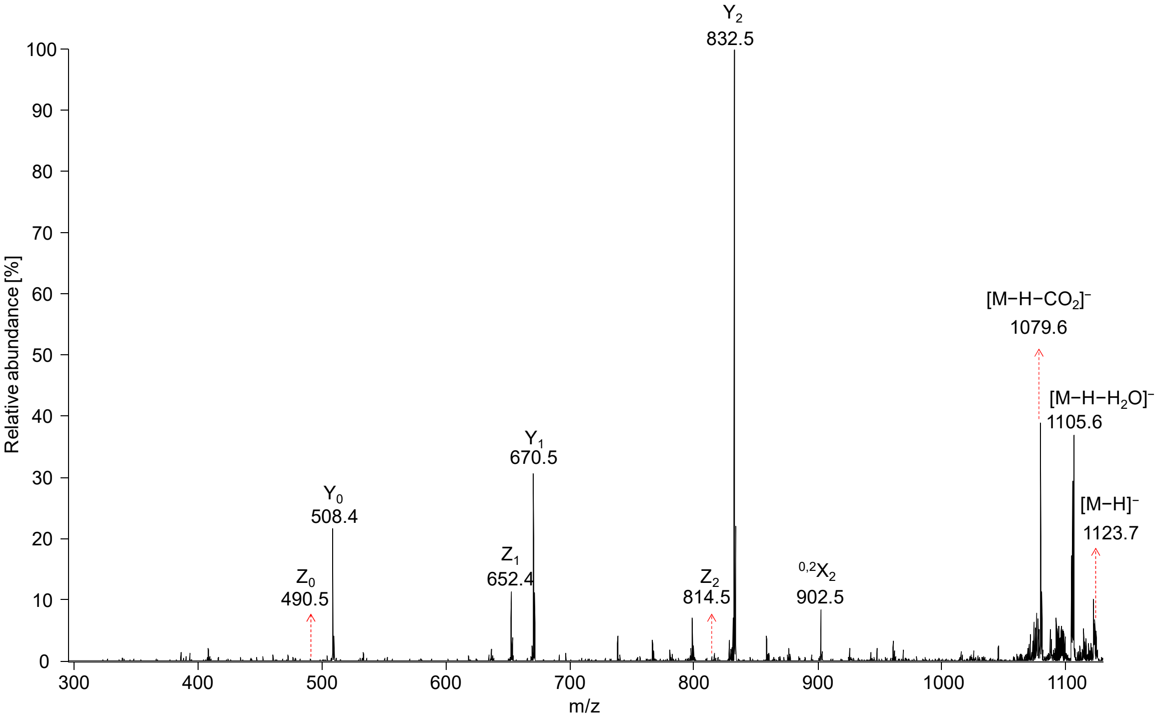

Figure S55. MS/MS spectrum of GM3 32:1 at *m/z* 1123.7 with characteristic fragments.

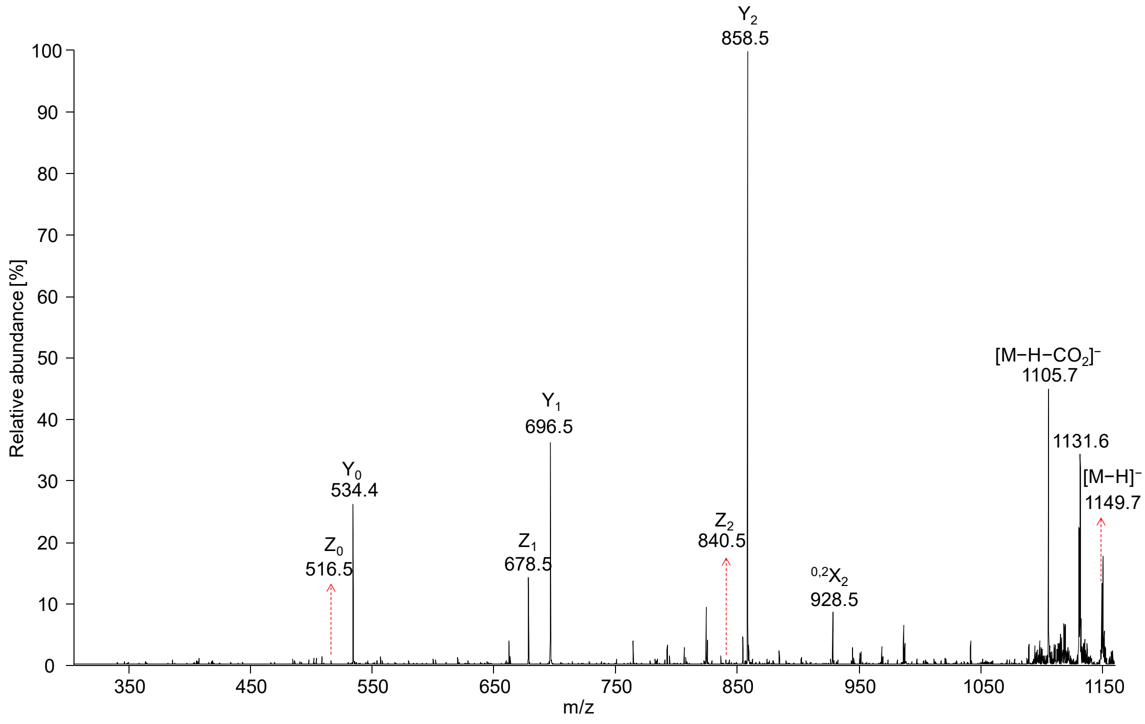

Figure S56. MS/MS spectrum of GM3 34:2 at *m/z* 1149.7 with characteristic fragments.

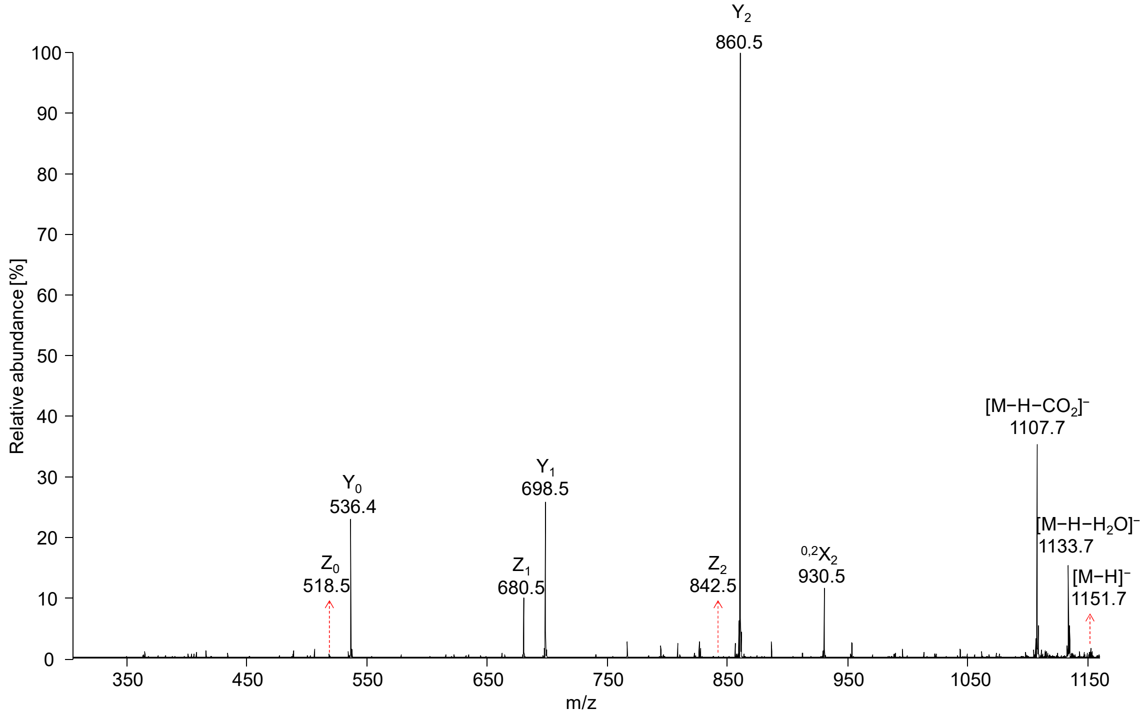

Figure S57. MS/MS spectrum of GM3 34:1 at *m/z* 1151.7 with characteristic fragments.

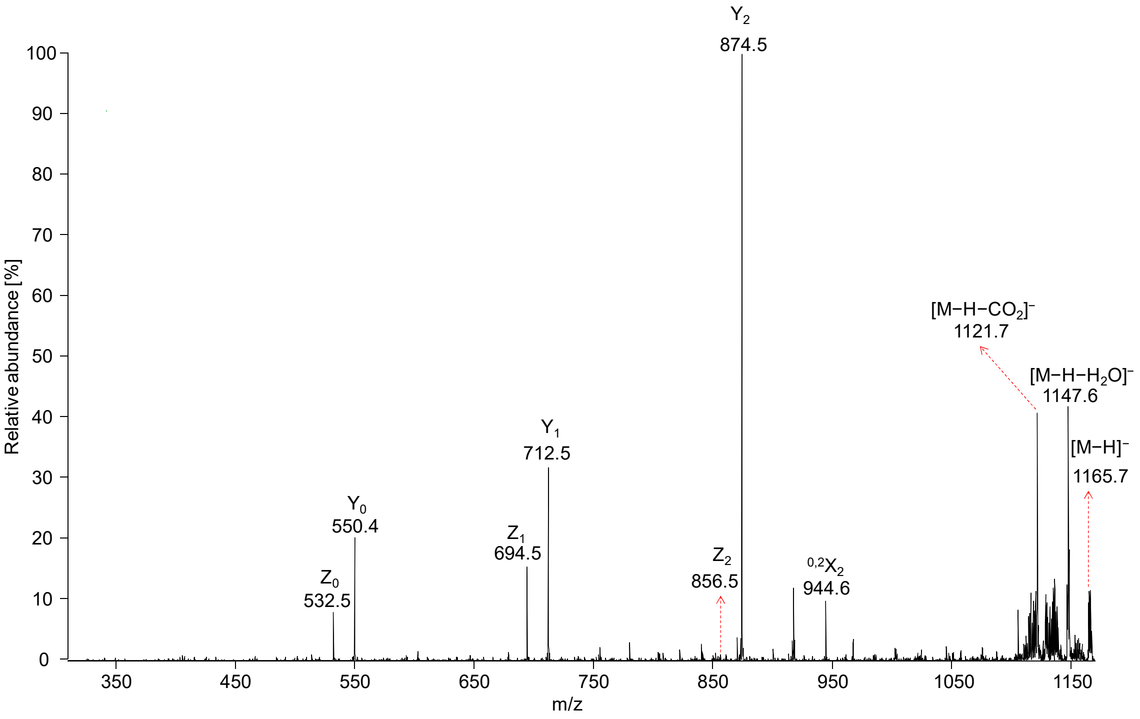

Figure S58. MS/MS spectrum of GM3 34:2 OH at *m/z* 1165.7 with characteristic fragments.

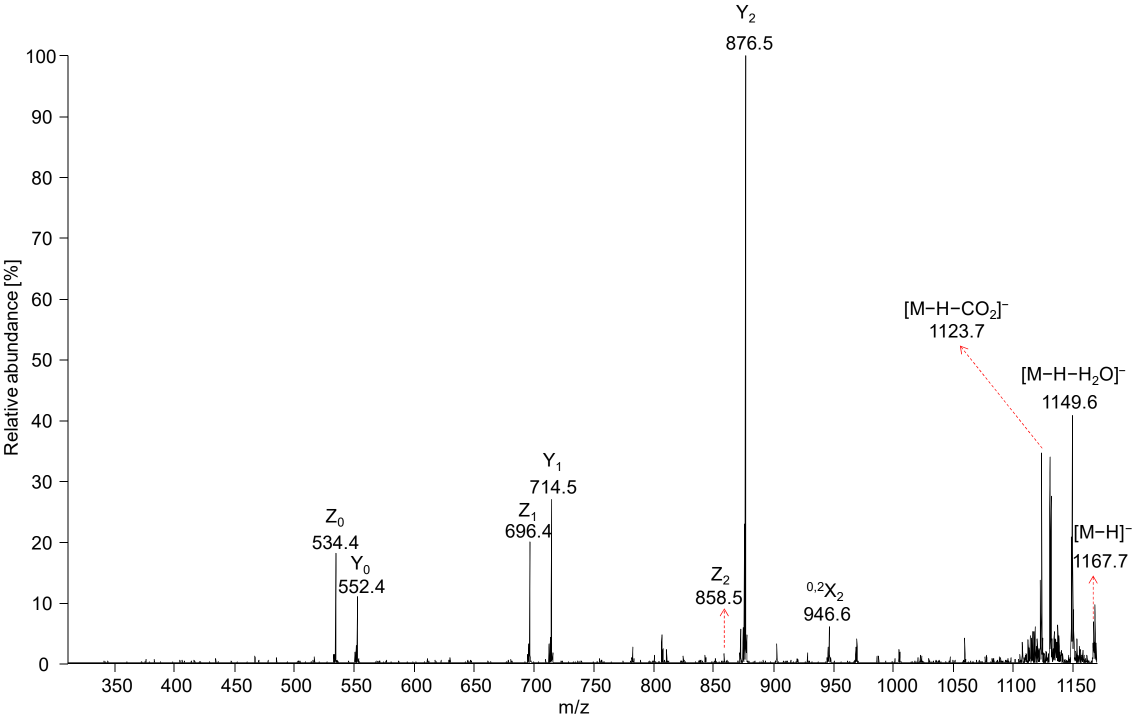

Figure S59. MS/MS spectrum of GM3 34:1 OH at *m/z* 1167.7 with characteristic fragments.

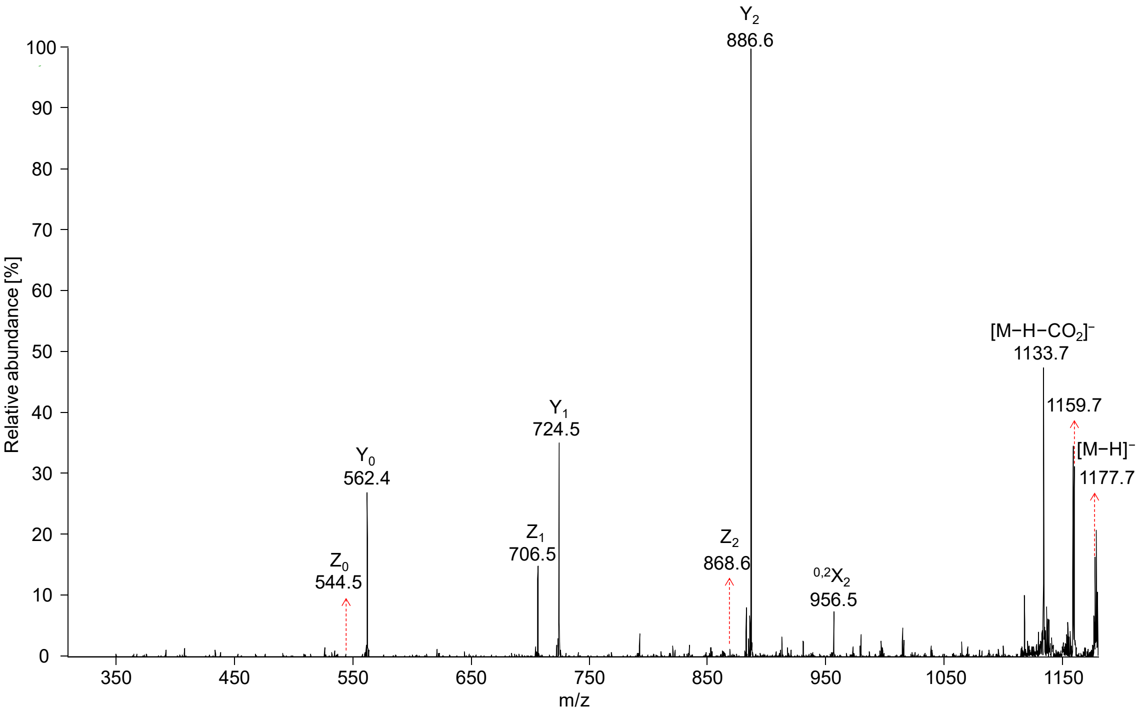

Figure S60. MS/MS spectrum of GM3 36:2 at *m/z* 1177.7 with characteristic fragments.

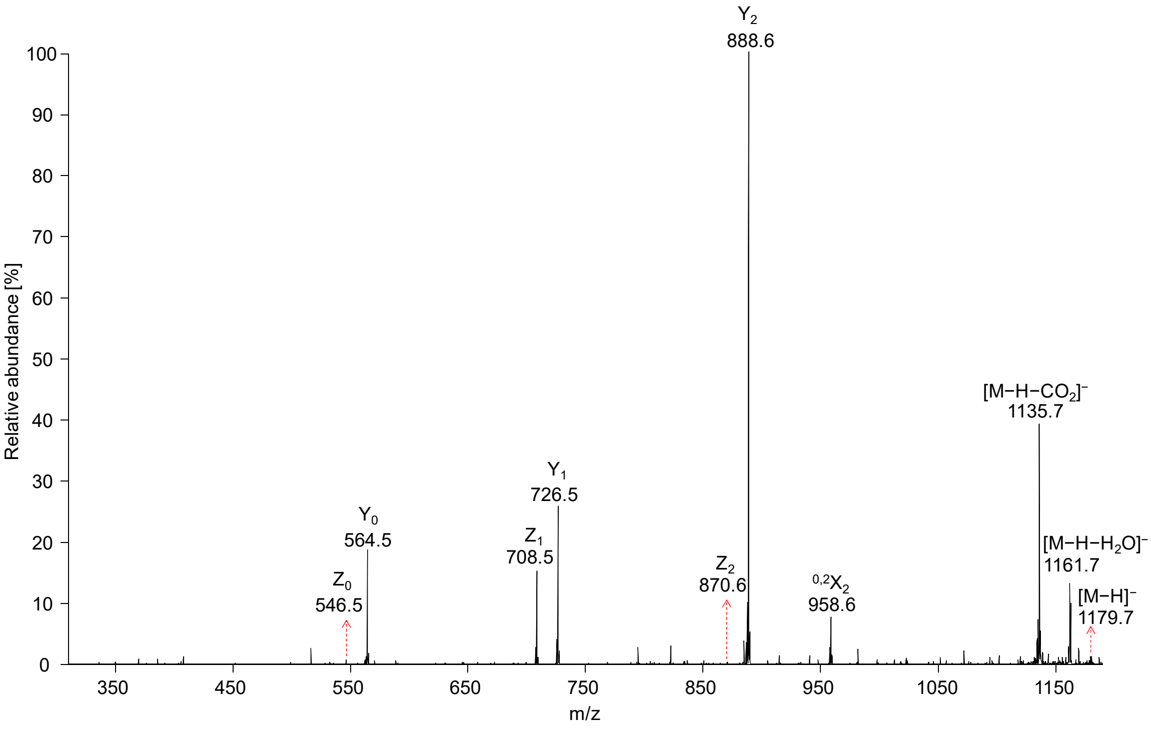

Figure S61. MS/MS spectrum of GM3 36:1 at *m/z* 1179.7 with characteristic fragments.

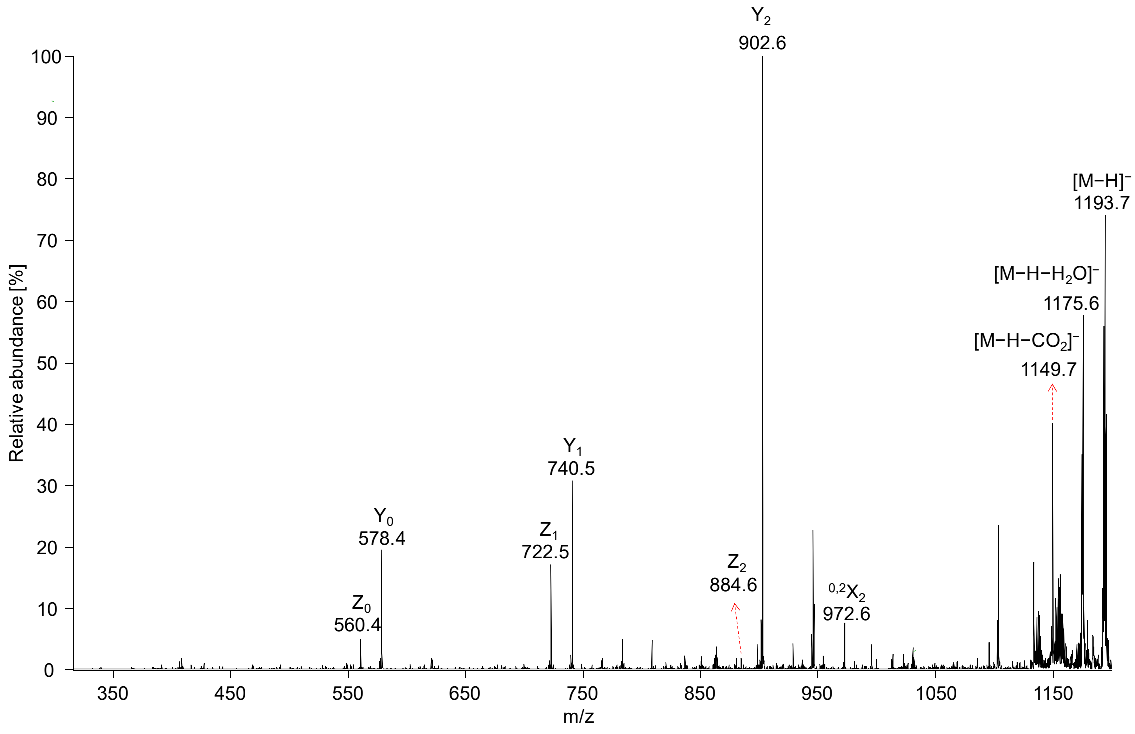

Figure S62. MS/MS spectrum of GM_3 36:2 OH at *m/z* 1193.7 with characteristic fragments.

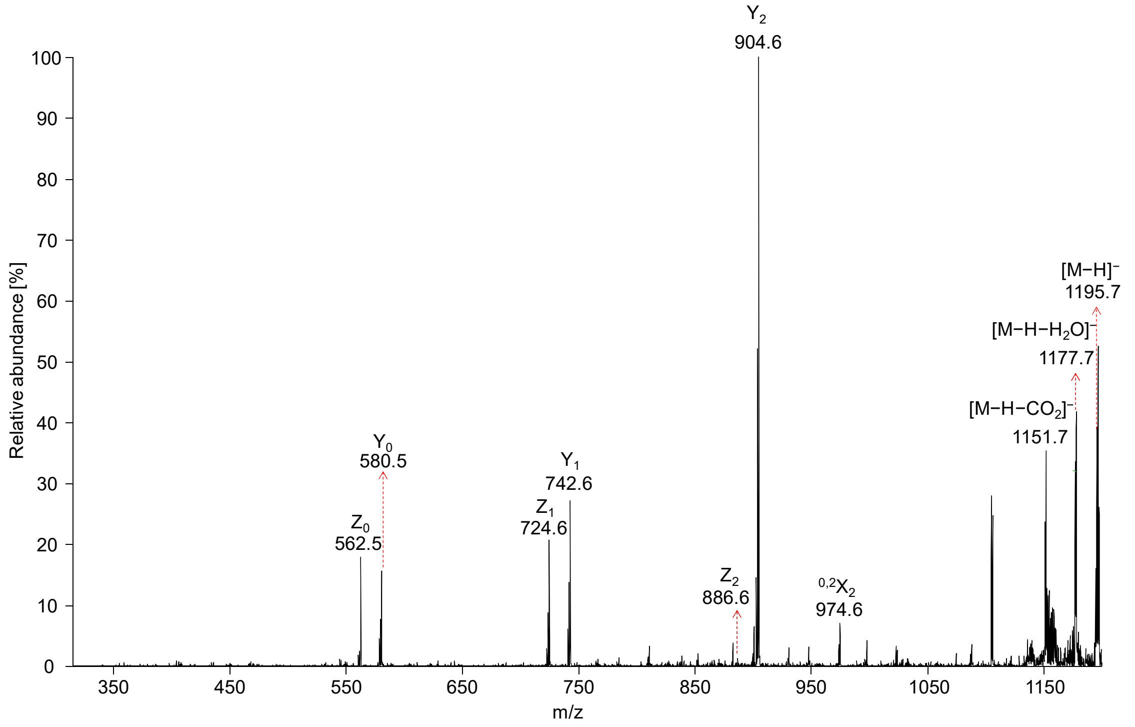

Figure S63. MS/MS spectrum of GM_3 36:1 OH at *m/z* 1195.7 with characteristic fragments.

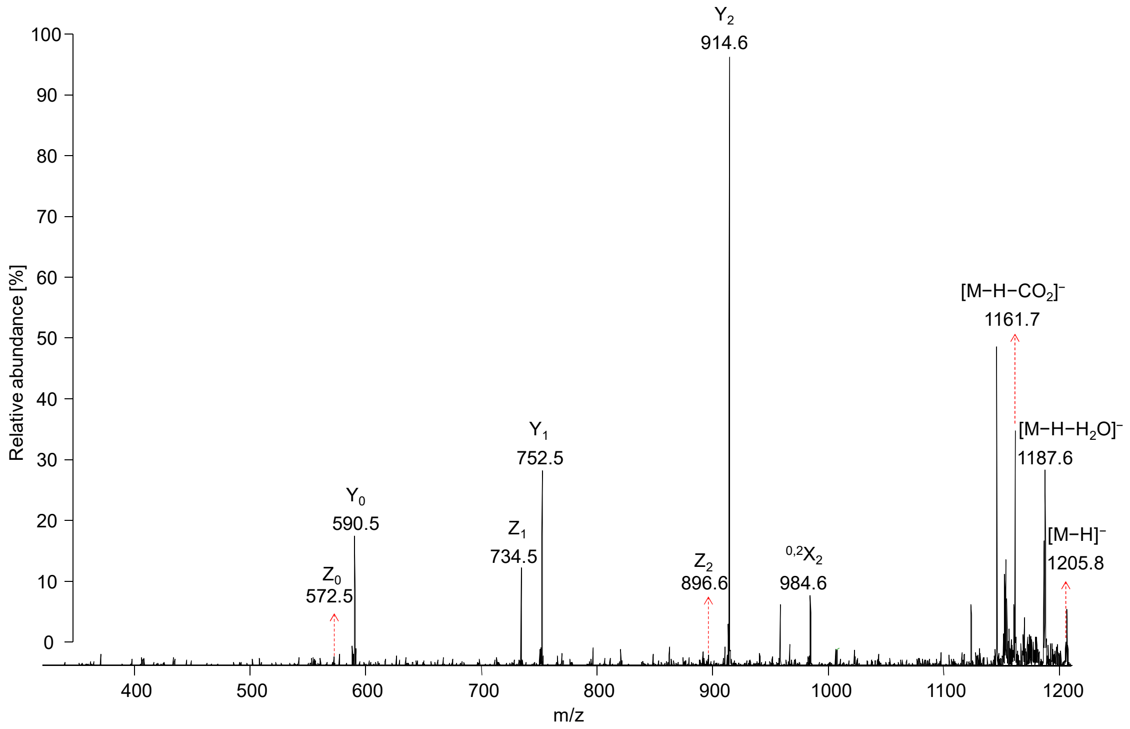

Figure S64. MS/MS spectrum of GM3 38:2 at *m/z* 1205.8 with characteristic fragments.

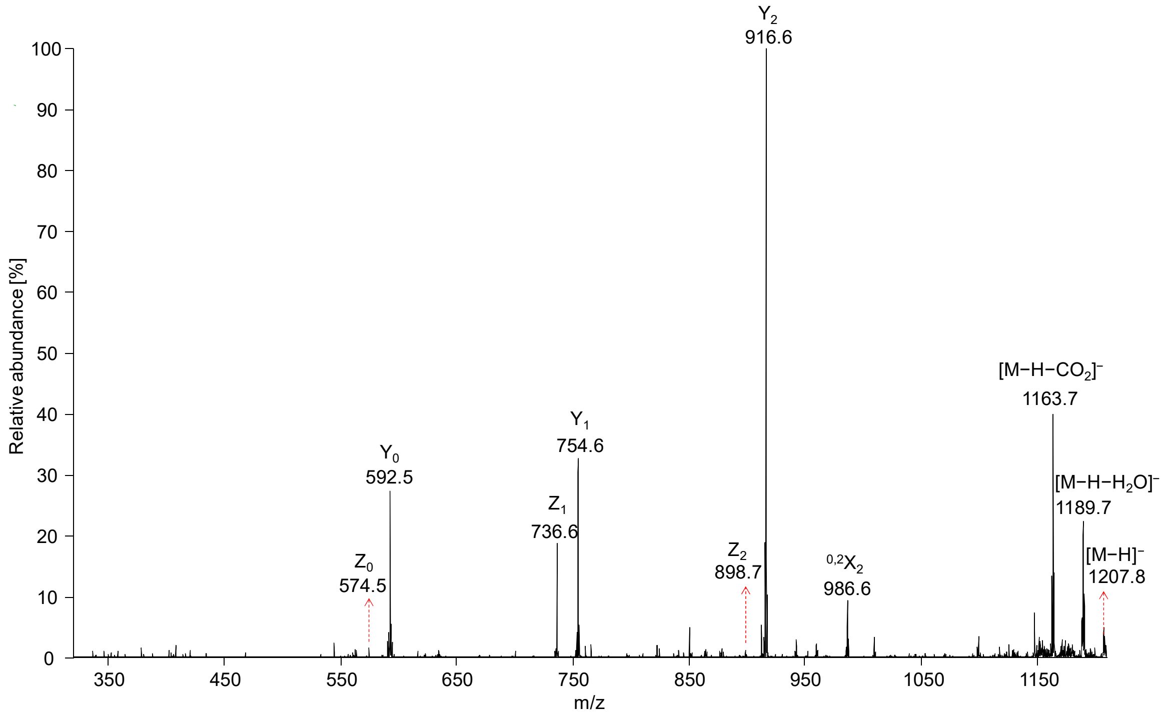

Figure S65. MS/MS spectrum of GM3 38:1 at *m/z* 1207.8 with characteristic fragments.

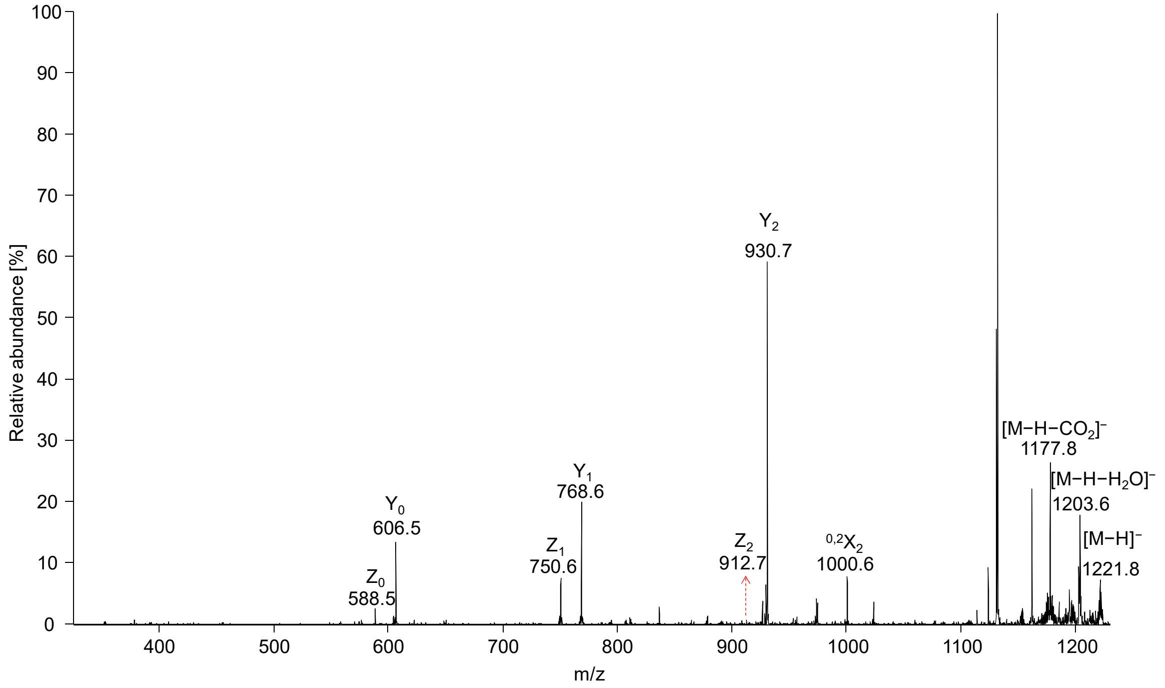

Figure S66. MS/MS spectrum of GM3 38:2 OH at *m/z* 1221.8 with characteristic fragments.

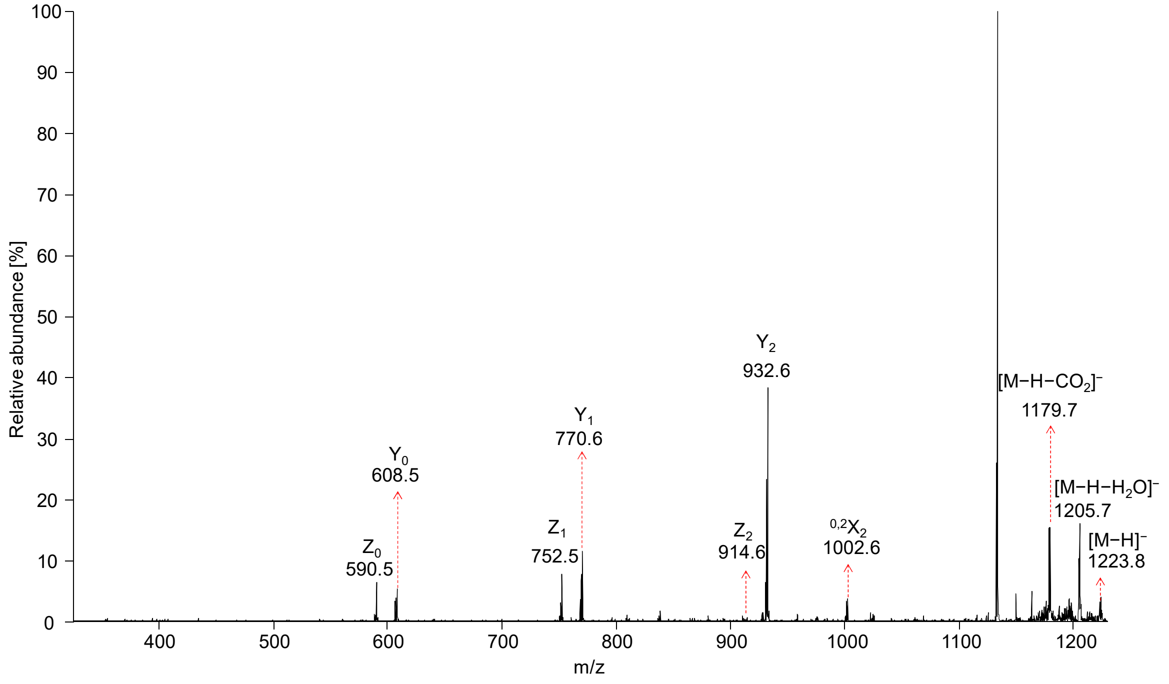

Figure S67. MS/MS spectrum of GM3 38:1 OH at *m/z* 1223.8 with characteristic fragments.

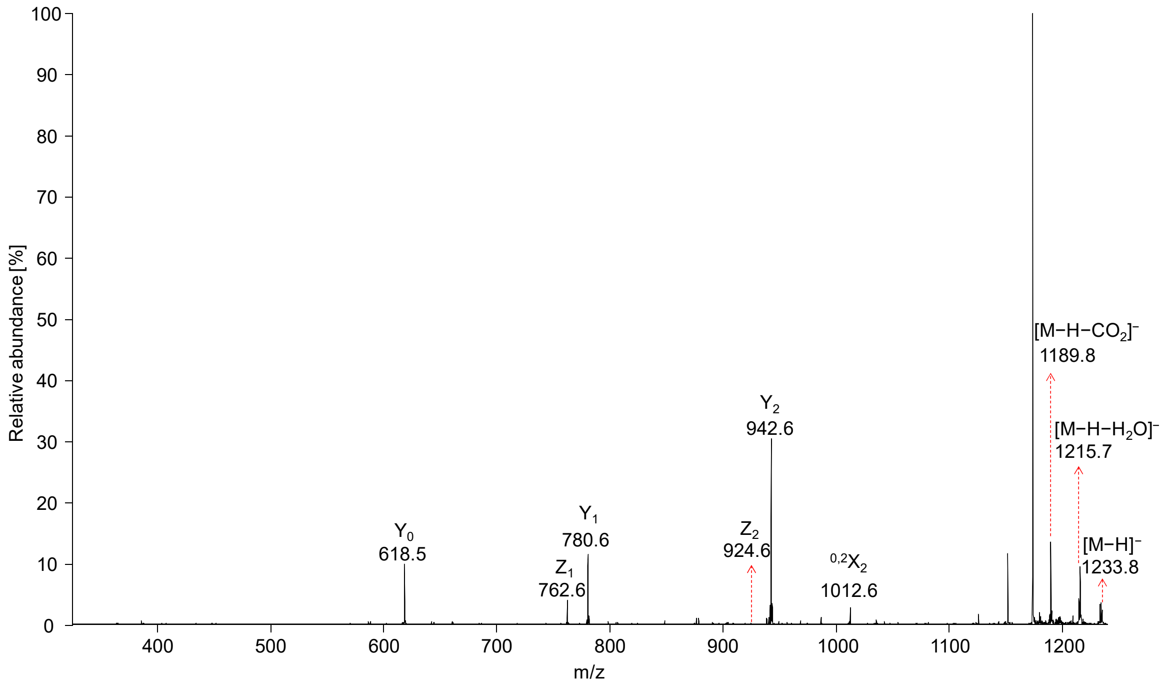

Figure S68. MS/MS spectrum of GM3 40:2 at *m/z* 1233.8 with characteristic fragments.

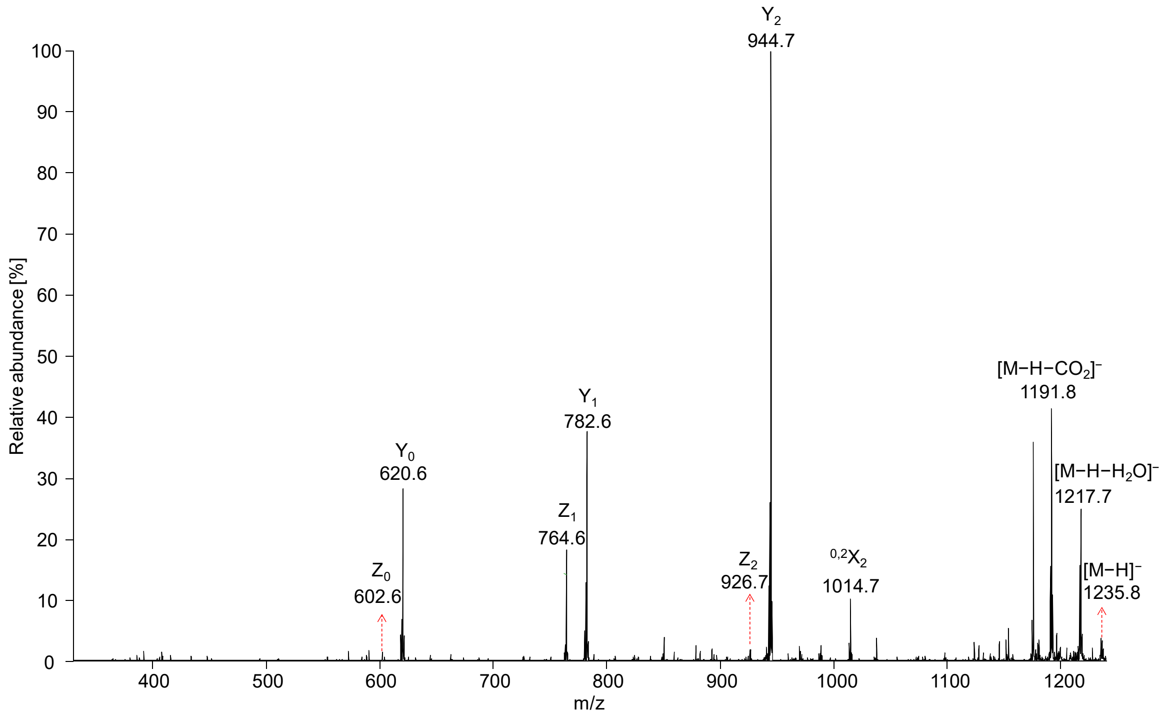

Figure S69. MS/MS spectrum of GM3 40:1 at *m/z* 1235.8 with characteristic fragments.

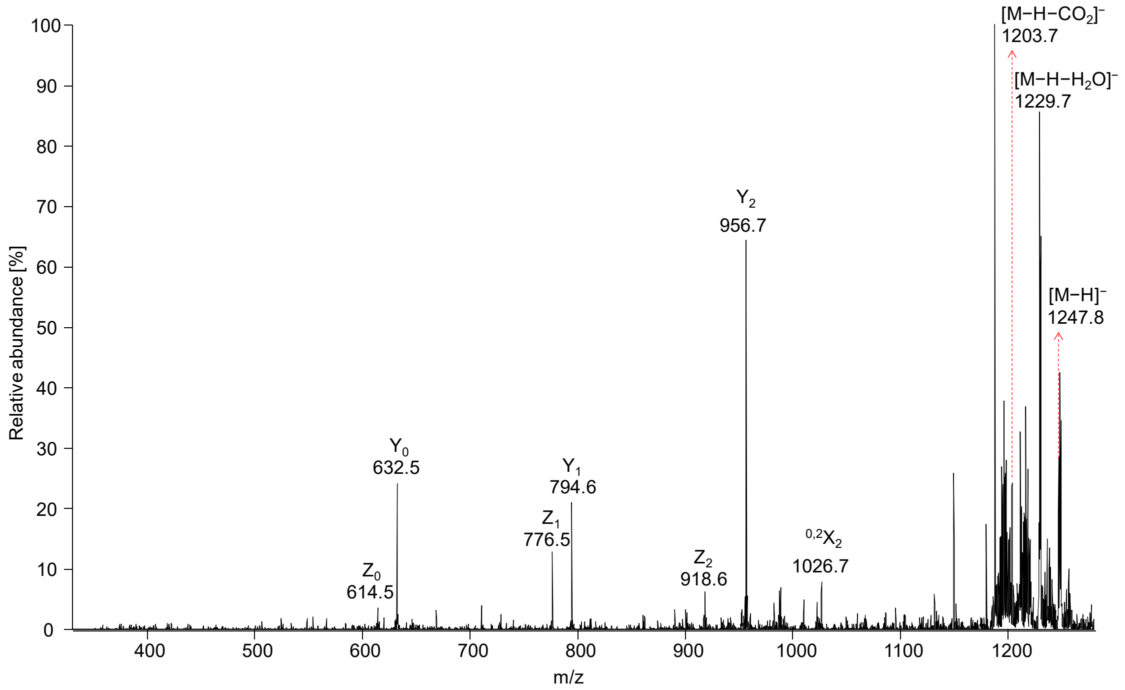

Figure S70. MS/MS spectrum of GM3 40:3 OH at *m/z* 1247.8 with characteristic fragments.

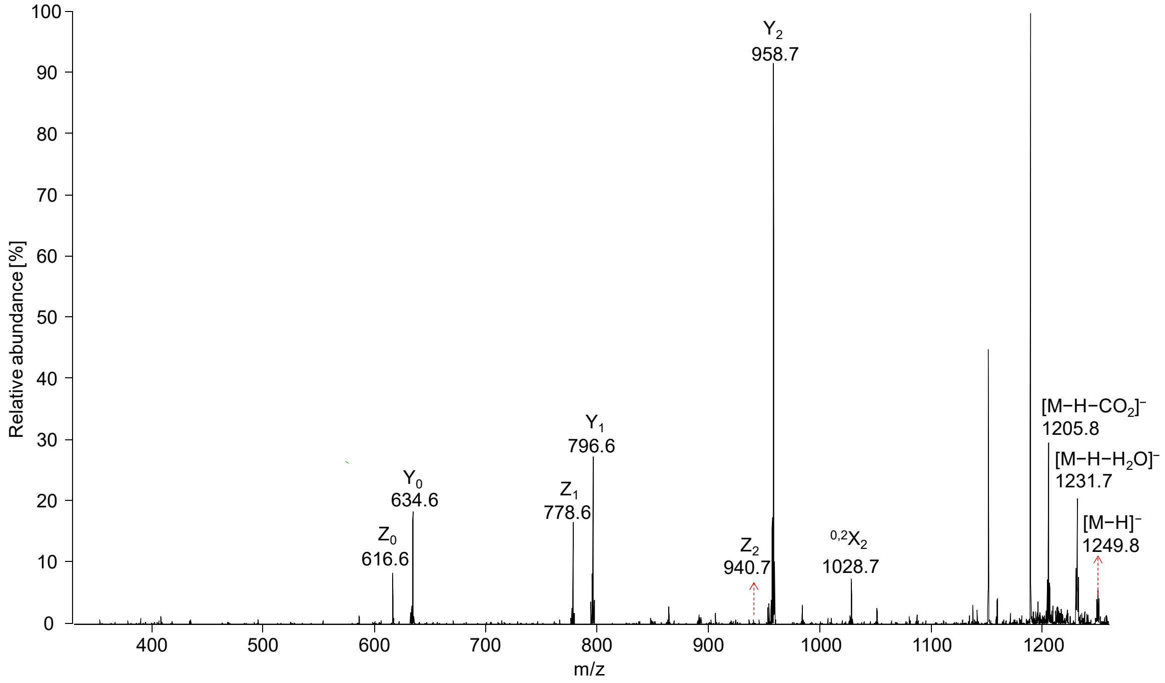

Figure S71. MS/MS spectrum of GM3 40:2 OH at *m/z* 1249.8 with characteristic fragments.

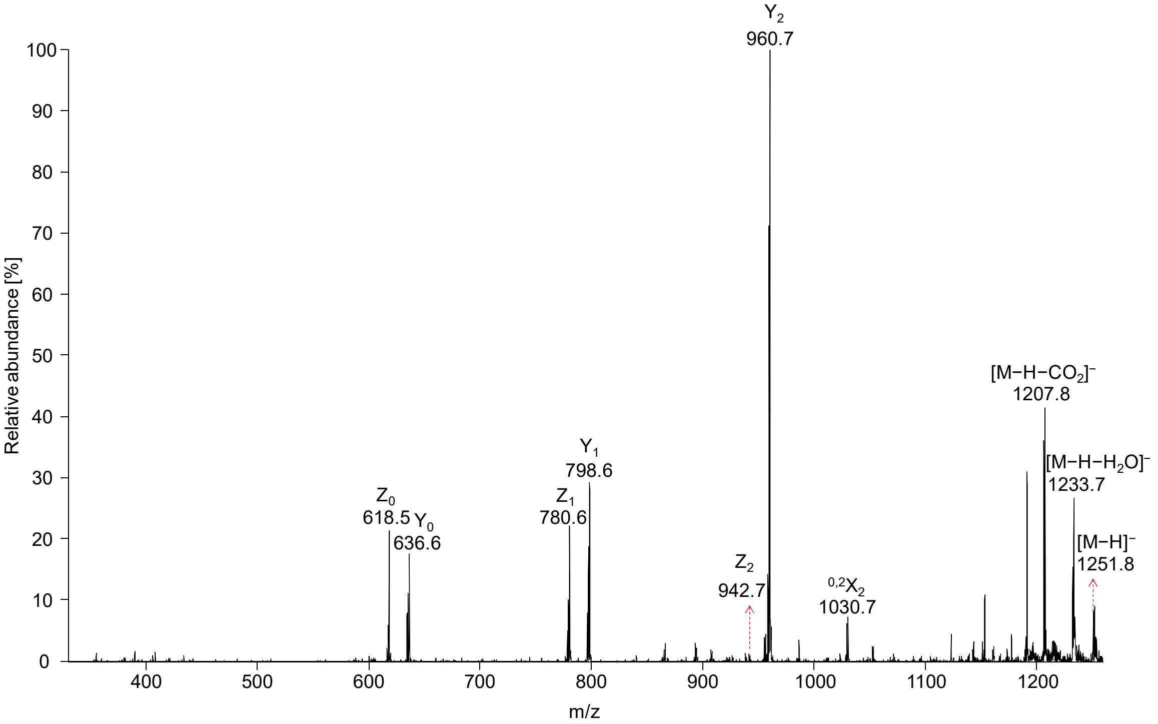

Figure S72. MS/MS spectrum of GM3 40:1 OH at *m/z* 1251.8 with characteristic fragments.

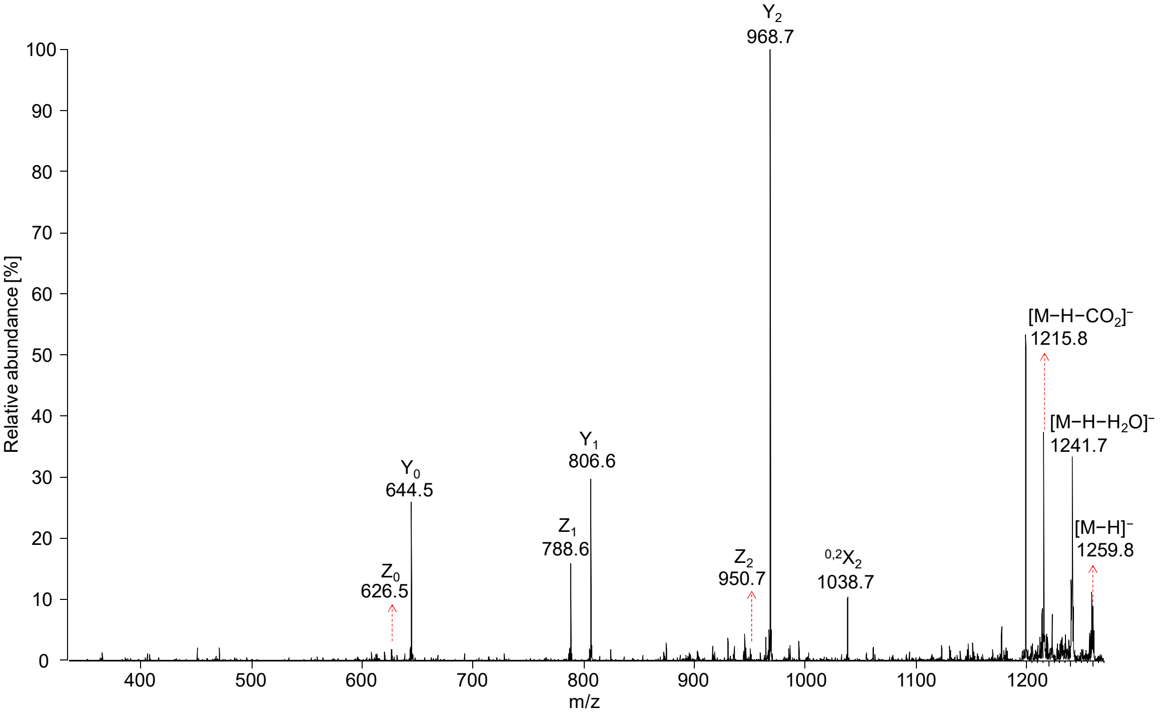

Figure S73. MS/MS spectrum of GM3 42:3 at *m/z* 1259.8 with characteristic fragments.

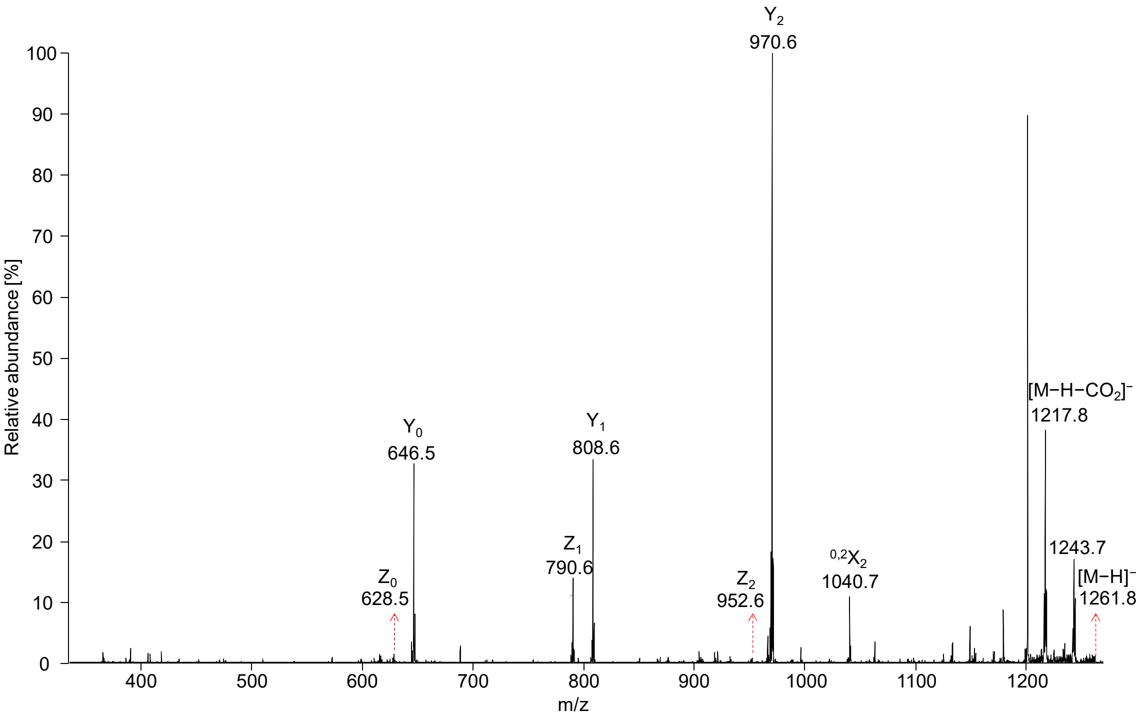

Figure S74. MS/MS spectrum of GM3 42:2 at *m/z* 1261.8 with characteristic fragments.

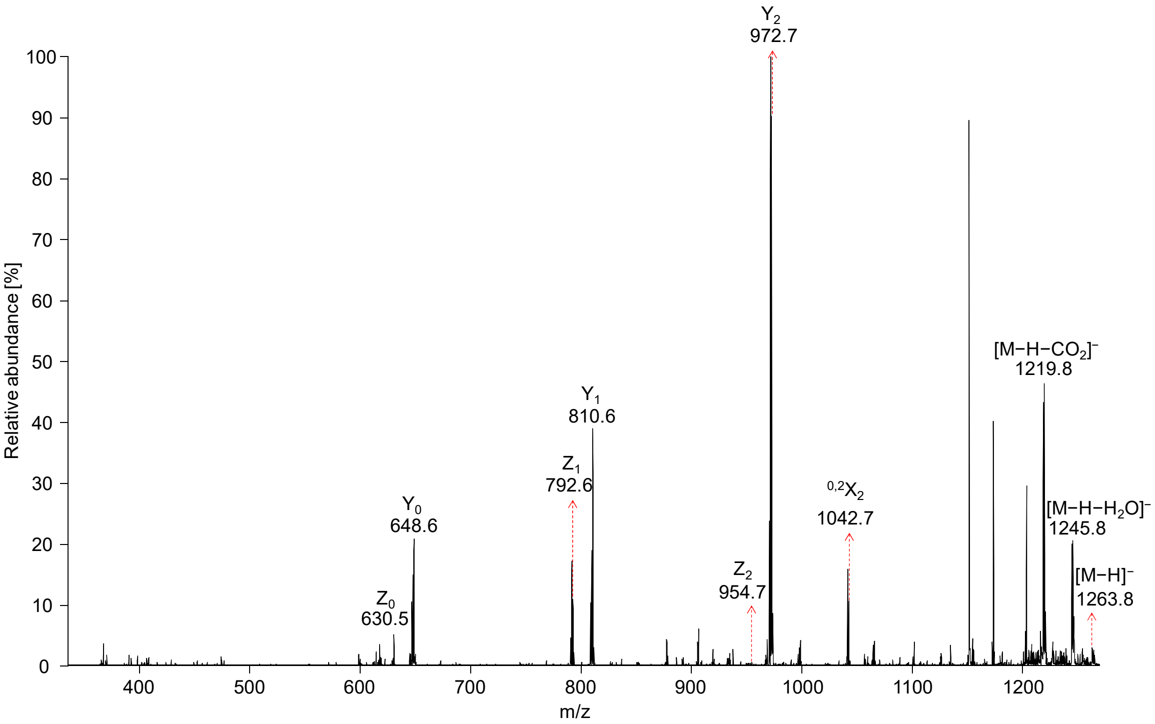

Figure S75. MS/MS spectrum of GM3 42:1 at *m/z* 1263.8 with characteristic fragments.

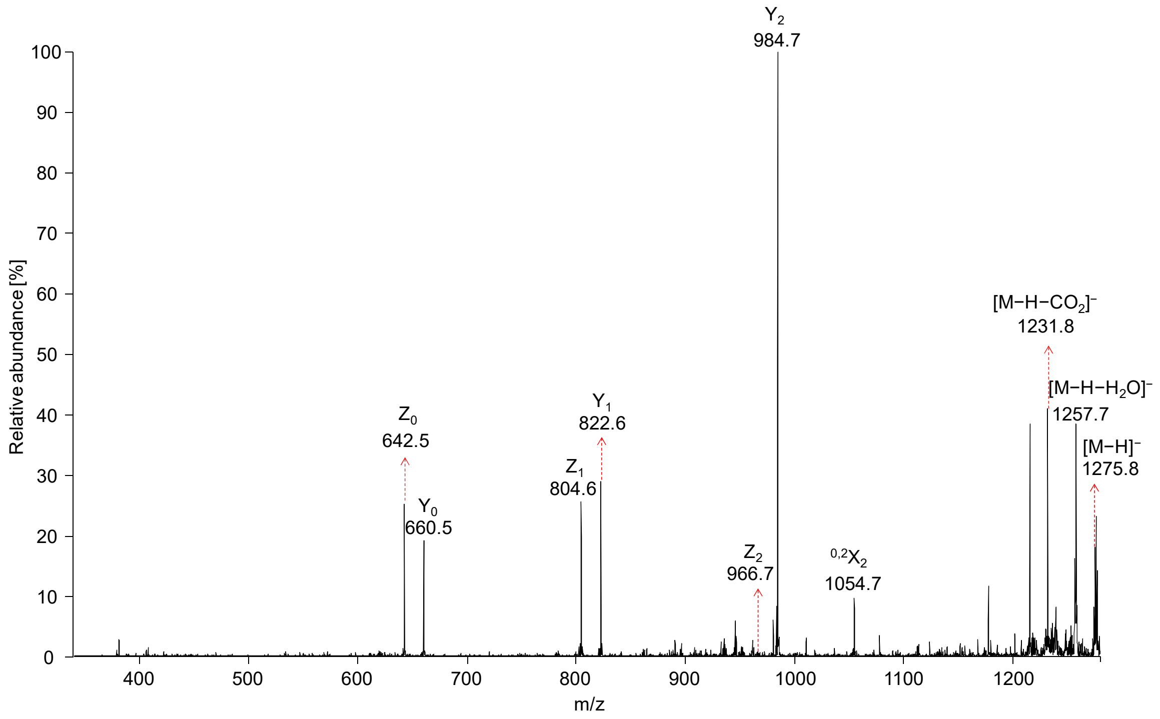

Figure S76. MS/MS spectrum of GM3 42:3 OH at *m/z* 1275.8 with characteristic fragments.

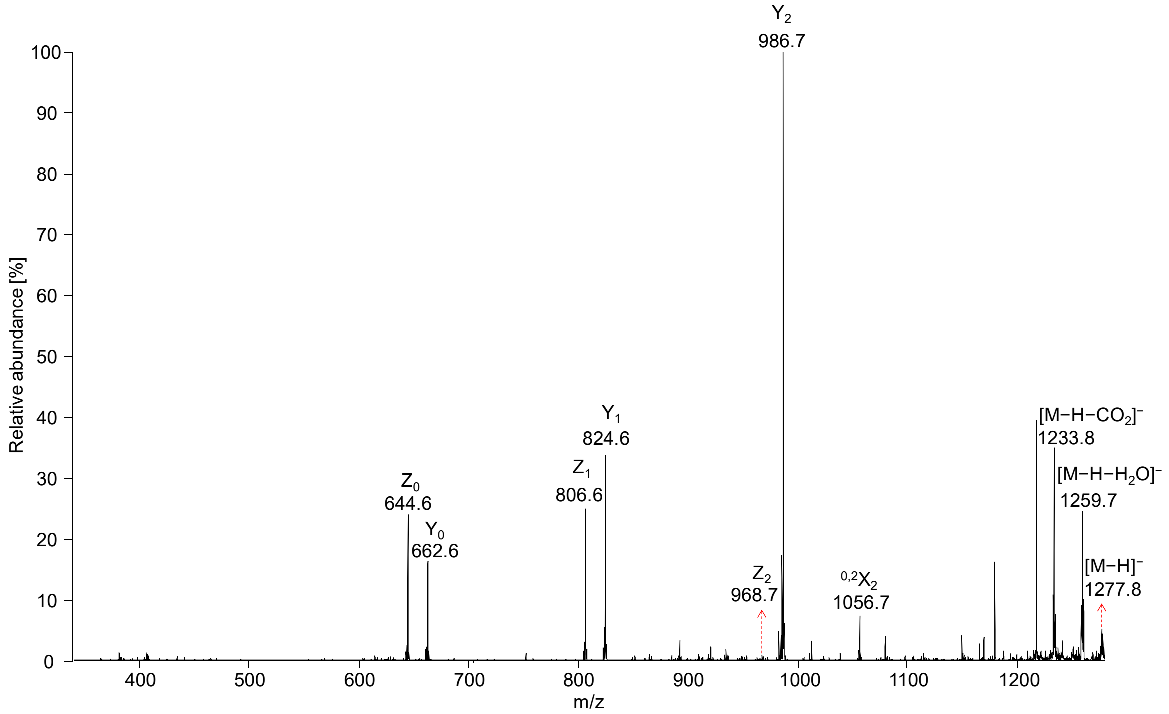

Figure S77. MS/MS spectrum of GM3 42:2 OH at *m/z* 1277.8 with characteristic fragments.

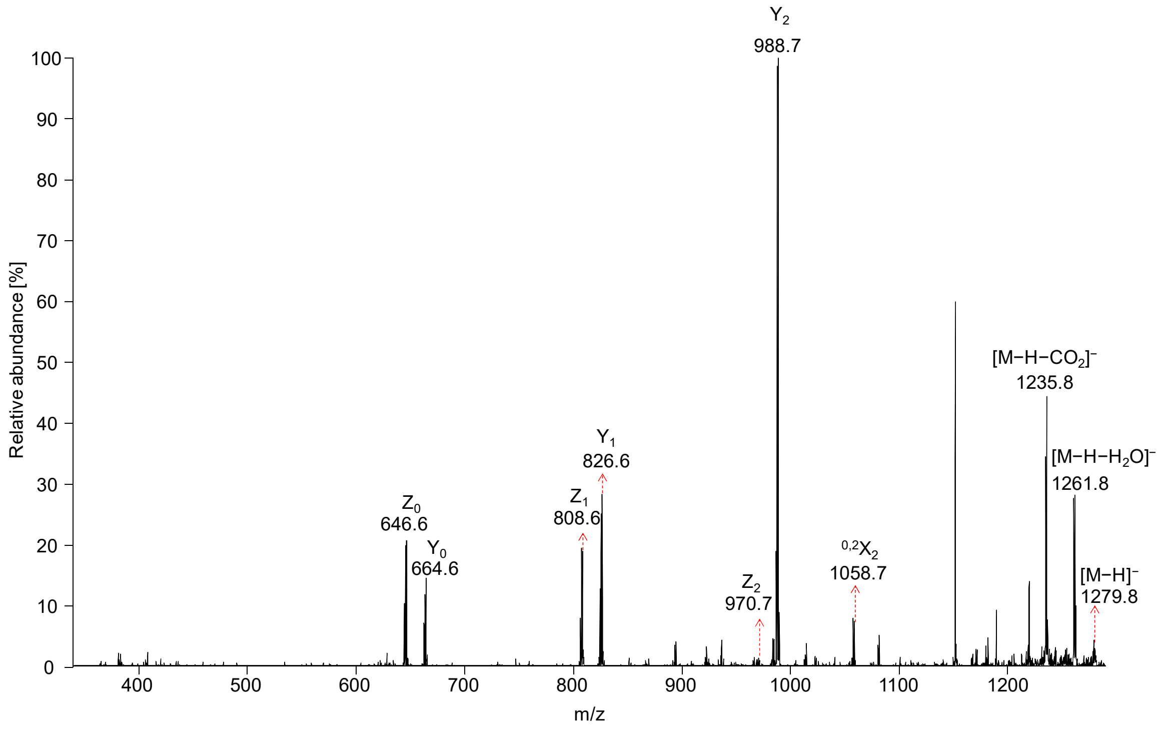

Figure S78. MS/MS spectrum of GM3 42:1 OH at *m/z* 1279.8 with characteristic fragments.

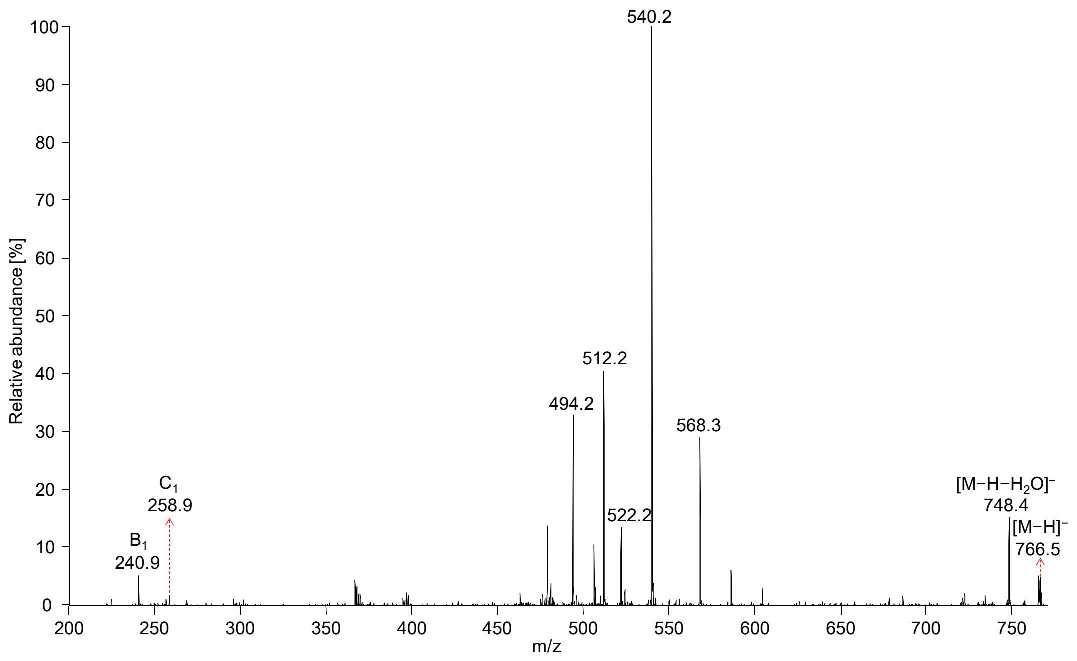

Figure S79. MS/MS spectrum of SHexCer 32:1 OH at *m/z* 766.5 with characteristic fragments. The presence of hydroxyl group is confirmed by the ion cluster at *m/z* 568, 540, and 522 (typical for sphingosine base 18:1), and *m/z* 540, 512, and 494 (typical for sphingosine base 16:1).

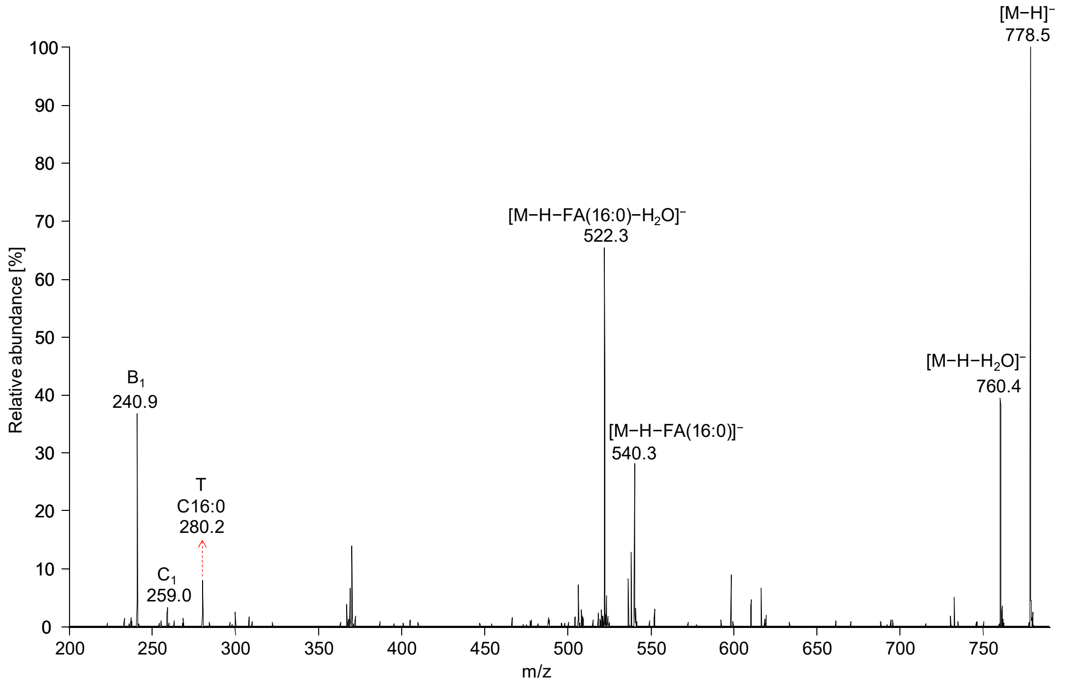

Figure S80. MS/MS spectrum of SHexCer 34:1 at *m/z* 778.5 with characteristic fragments.

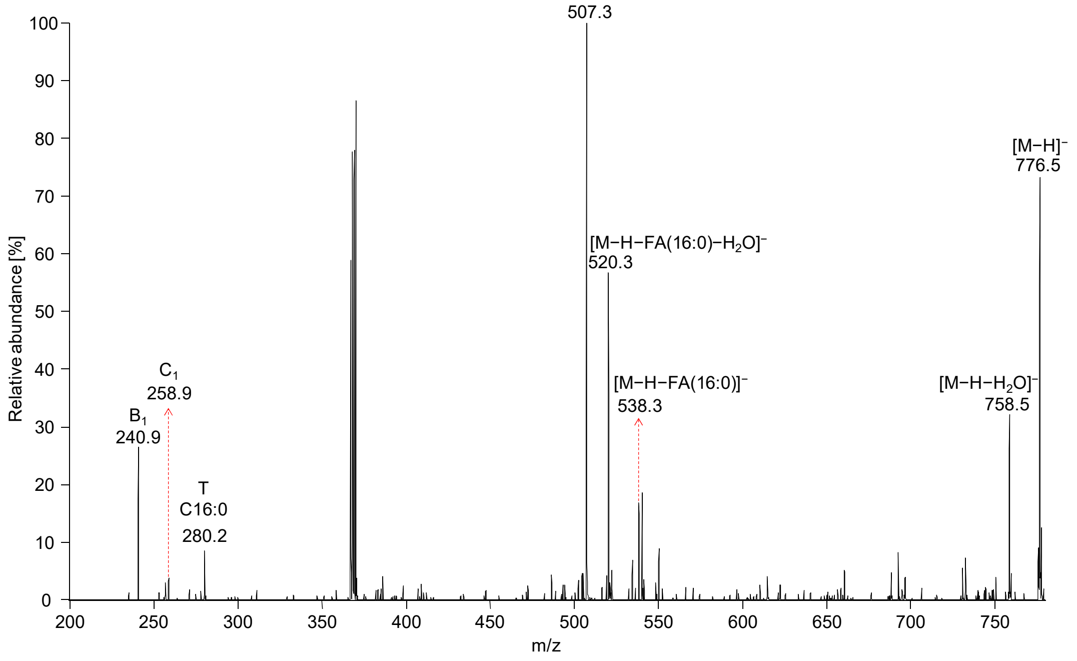

Figure S81. MS/MS spectrum of SHexCer 34:2 at *m/z* 776.5 with characteristic fragments.

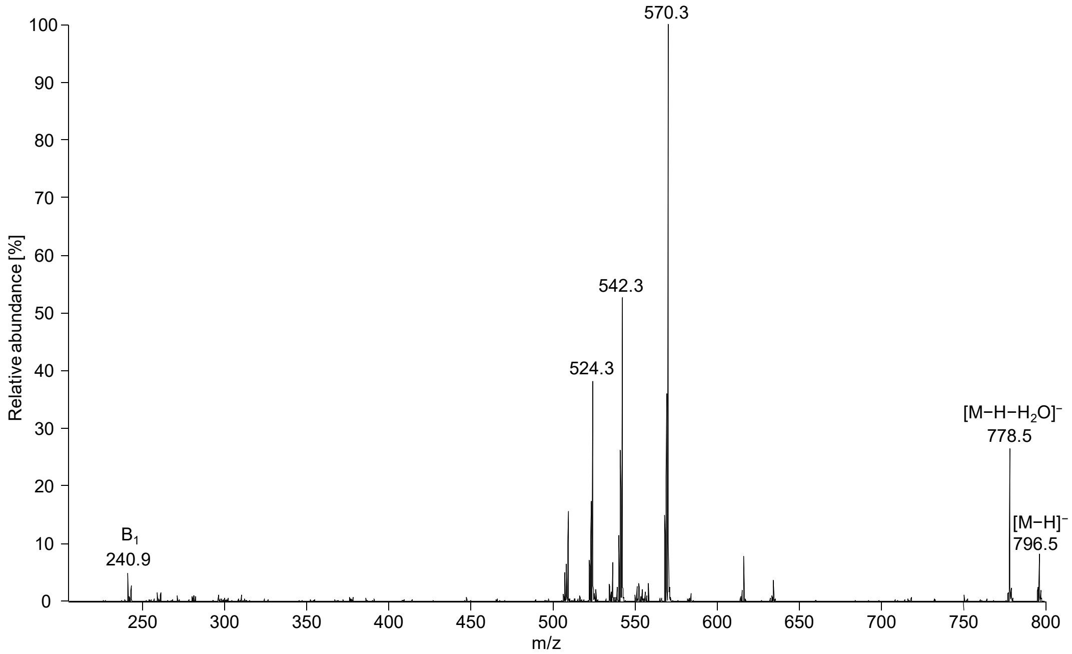

Figure S82. MS/MS spectrum of SHexCer 34:0 OH at *m/z* 796.5 with characteristic fragments. The presence of hydroxyl group is confirmed by the ion cluster at *m/z* 570, 542, and 524 (typical for sphingosine base 18:0).

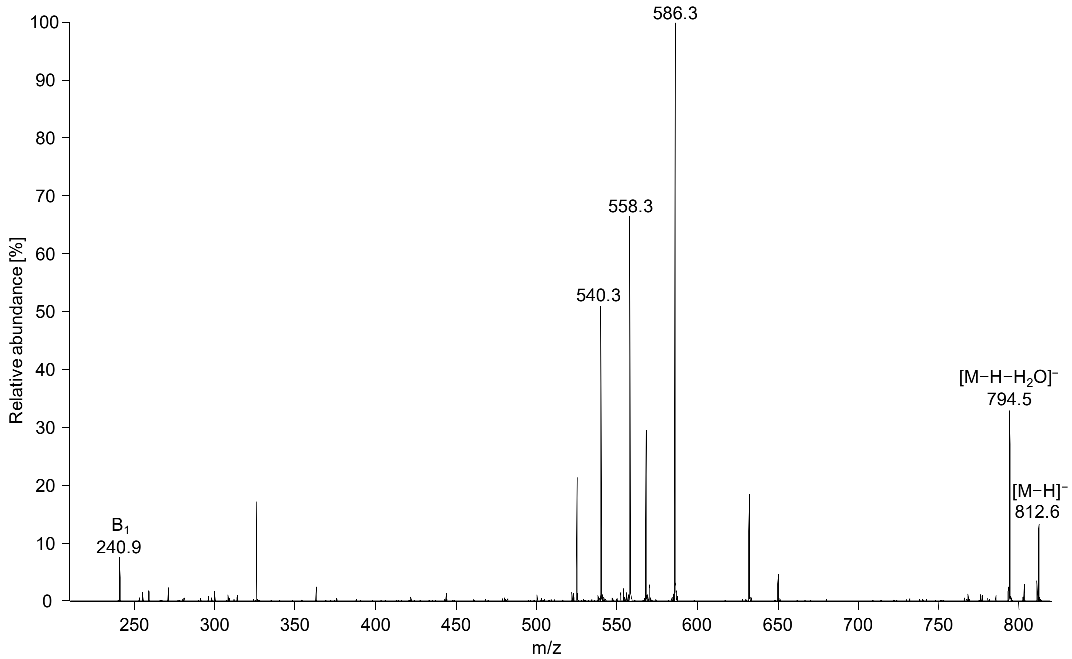

Figure S83. MS/MS spectrum of SHexCer 34:0 2OH at *m/z* 812.6 with characteristic fragments. The presence of two hydroxyl group is confirmed by the ion cluster at *m/z* 586, 558, and 540 (typical for sphingosine base 18:1 OH).

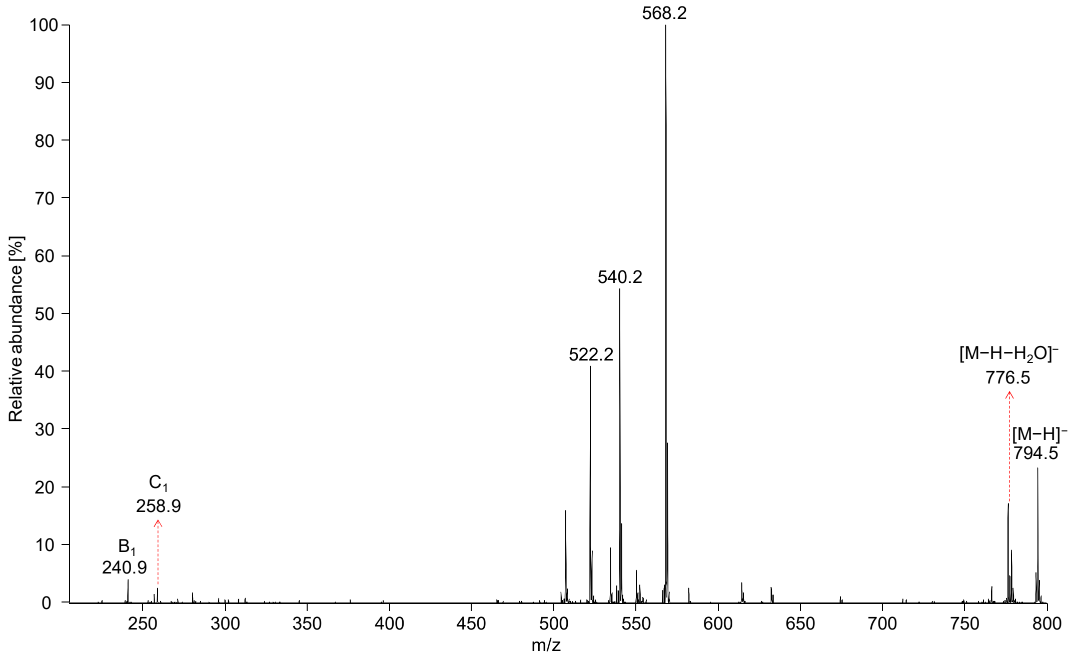

Figure S84. MS/MS spectrum of SHexCer 34:1 OH at *m/z* 794.5 with characteristic fragments. The presence of hydroxyl group is confirmed by the ion cluster at *m/z* 568, 540, and 522 (typical for sphingosine base 18:1).

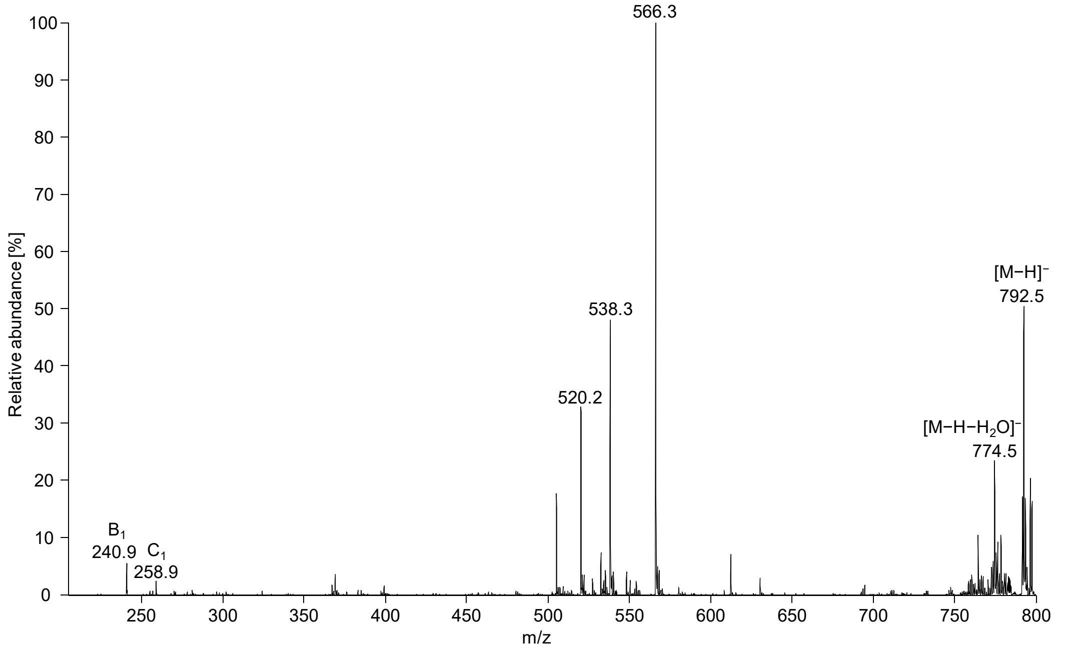

Figure S85. MS/MS spectrum of SHexCer 34:2 OH at *m/z* 792.5 with characteristic fragments. The presence of hydroxyl group is confirmed by the ion cluster at *m/z* 566, 538, and 520 (typical for sphingosine base 18:2).

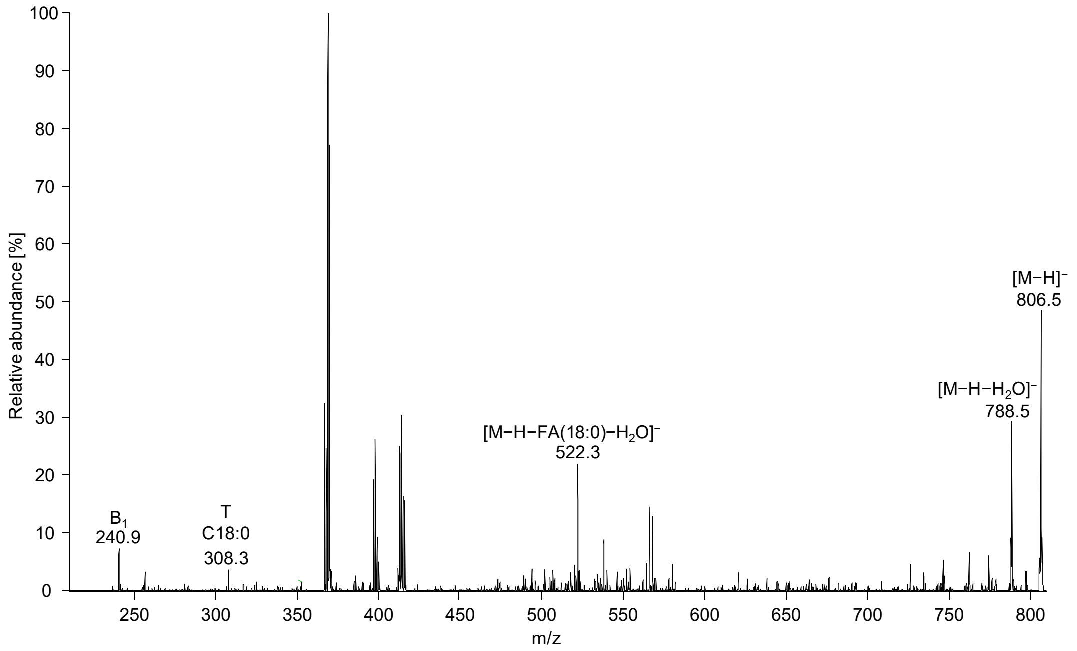

Figure S86. MS/MS spectrum of SHexCer 36:1 at *m/z* 806.5 with characteristic fragments.

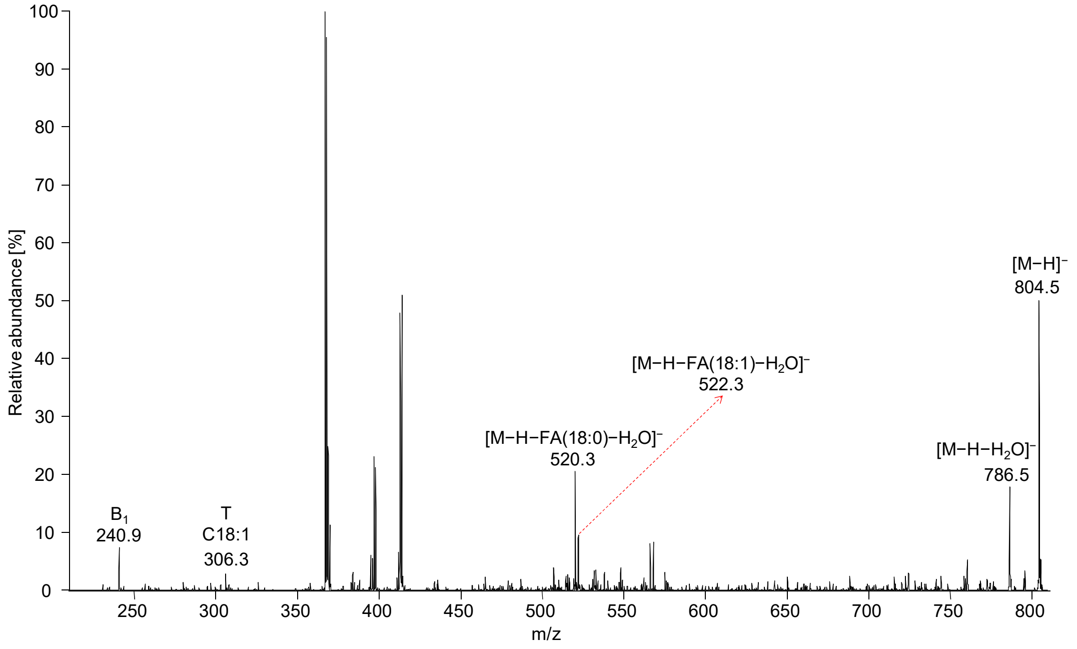

Figure S87. MS/MS spectrum of SHexCer 36:2 at *m/z* 804.5 with characteristic fragments.

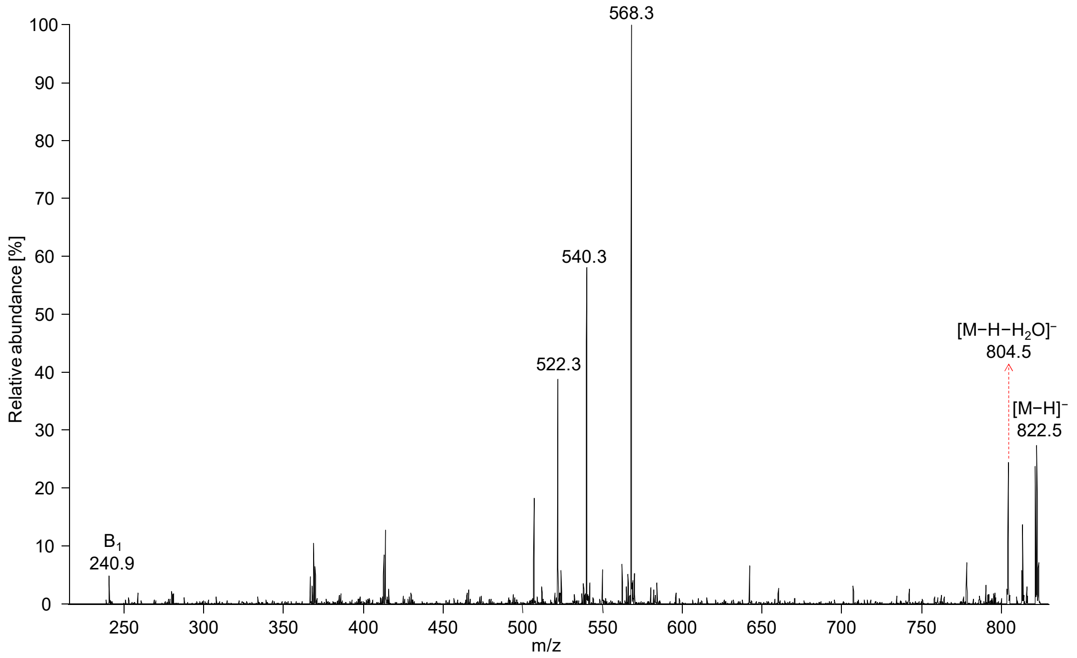

Figure S88. MS/MS spectrum of SHexCer 36:1 OH at *m/z* 822.5 with characteristic fragments. The presence of hydroxyl group is confirmed by the ion cluster at *m/z* 568, 540, and 522 (typical for sphingosine base 18:1).

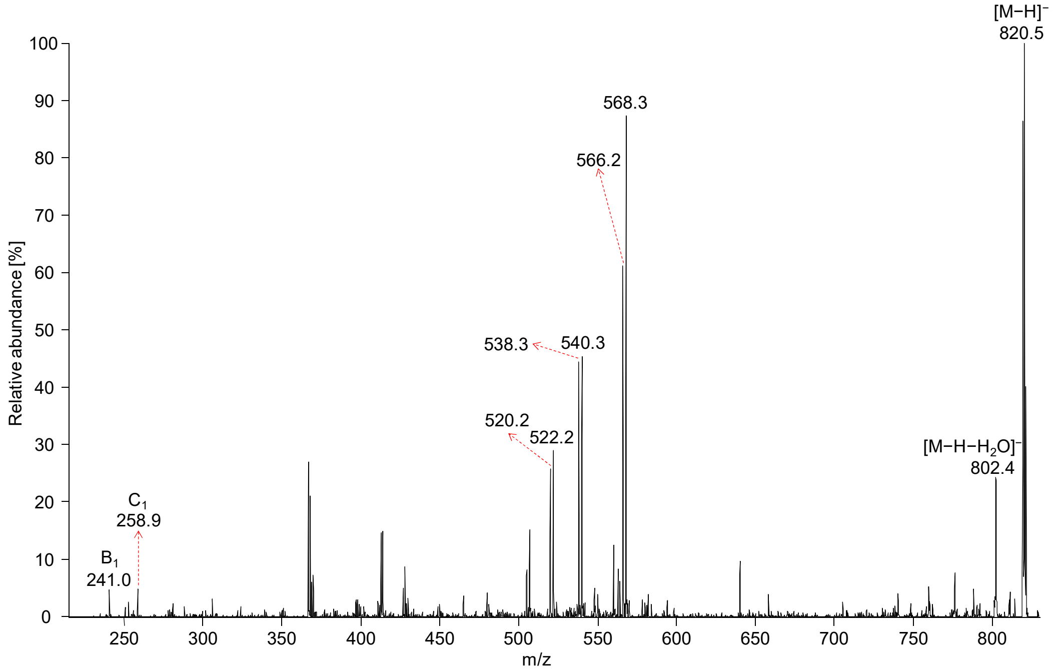

Figure S89. MS/MS spectrum of SHexCer 36:2 OH at *m/z* 820.5 with characteristic fragments. The presence of hydroxyl group is confirmed by the ion cluster at *m/z* 568, 540, and 522 (typical for sphingosine base 18:1) and *m/z* 566, 538, and 520 (typical for sphingosine base 18:2).

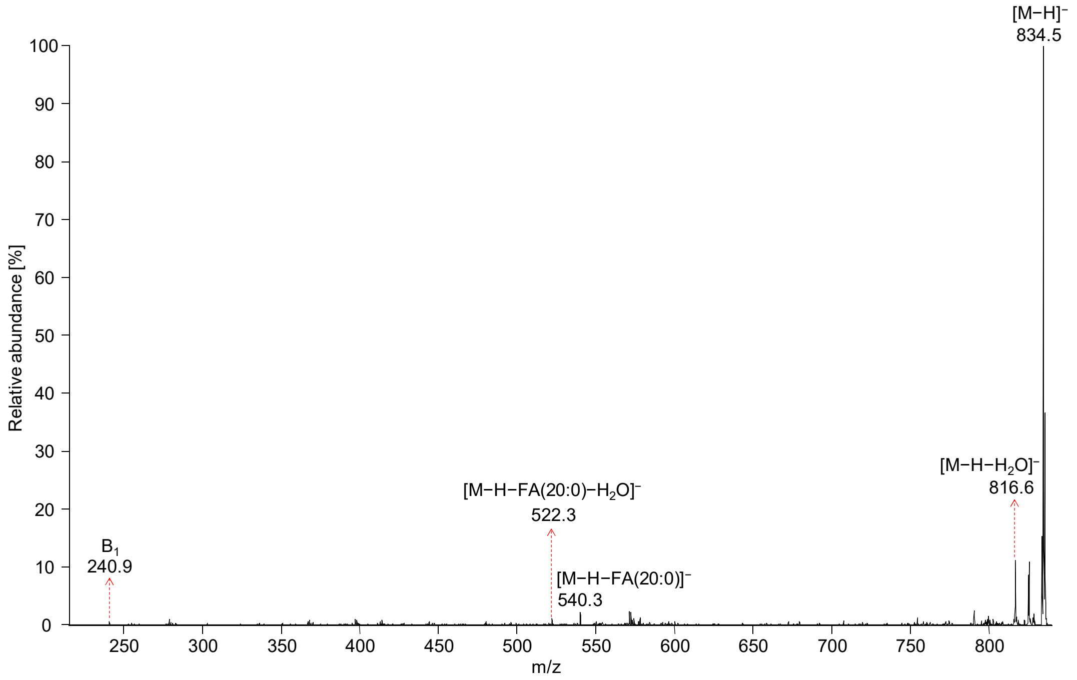

Figure S90. MS/MS spectrum of SHexCer 38:1 at *m/z* 834.5 with characteristic fragments.

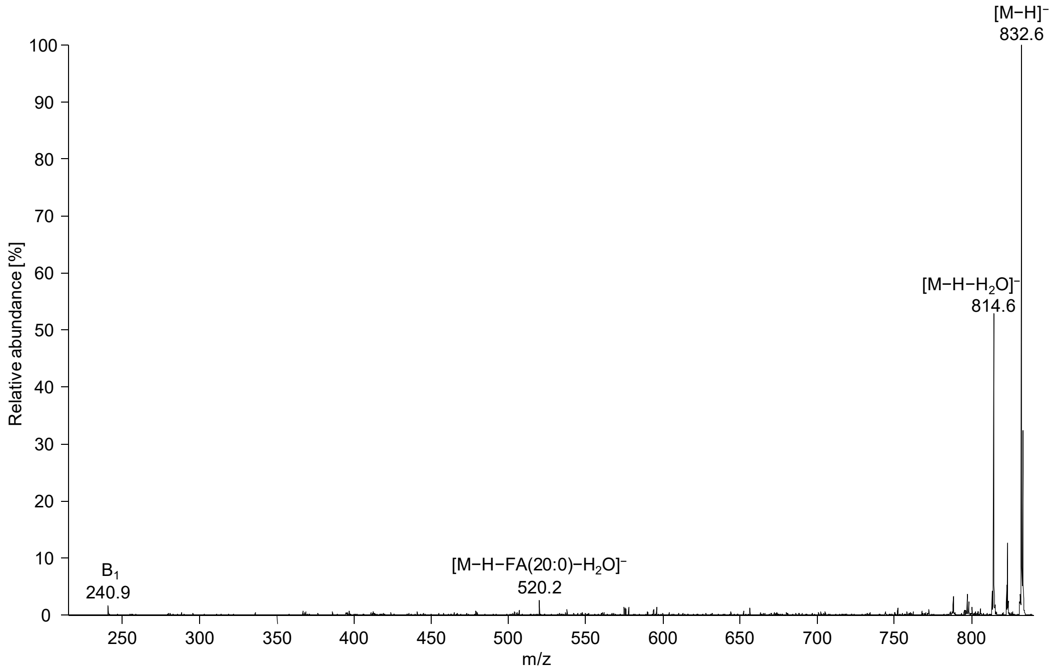

Figure S91. MS/MS spectrum of SHexCer 38:2 at *m/z* 832.6 with characteristic fragments.

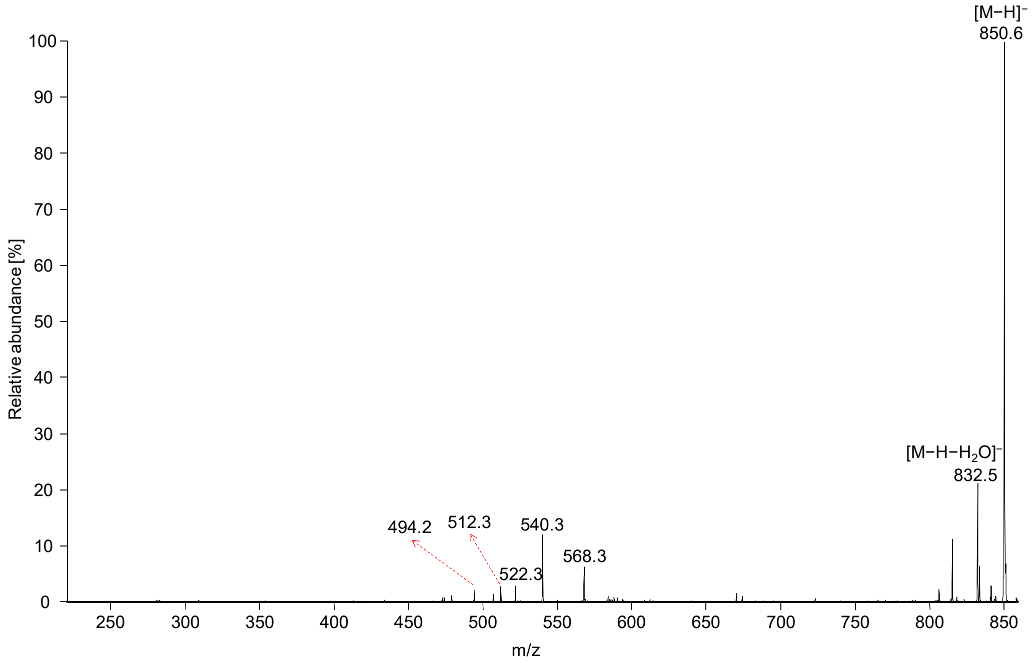

Figure S92. MS/MS spectrum of SHexCer 38:1 OH at *m/z* 850.6 with characteristic fragments. The presence of hydroxyl group is confirmed by the ion cluster at *m/z* 568, 540, and 522 (typical for sphingosine base 18:1) and *m/z* 540, 512, and 494 (typical for sphingosine base 16:1).

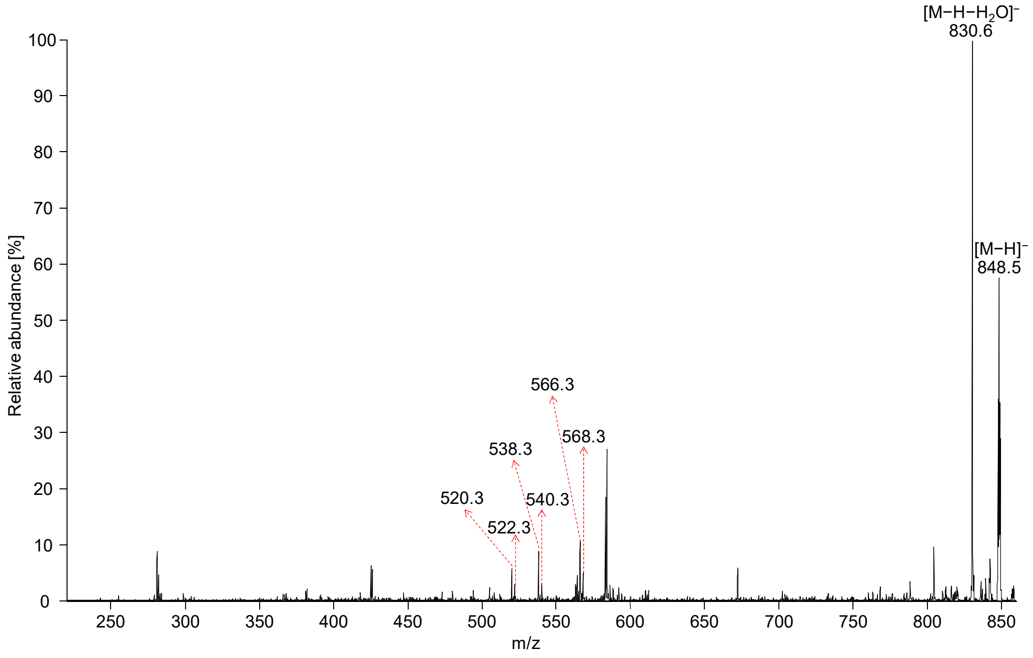

Figure S93. MS/MS spectrum of SHexCer 38:2 OH at *m/z* 848.5 with characteristic fragments. The presence of hydroxyl group is confirmed by the ion cluster at *m/z* 568, 540, and 522 (typical for sphingosine base 18:1) and *m/z* 566, 538, and 520 (typical for sphingosine base 18:2).

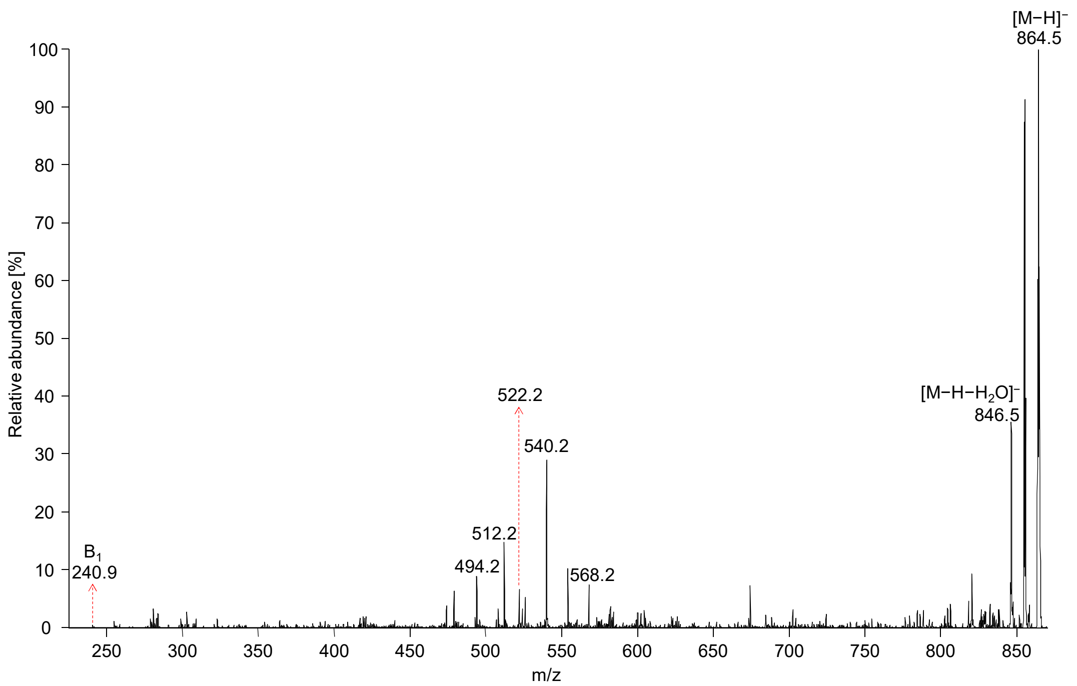

Figure S94. MS/MS spectrum of SHexCer 39:1 OH at *m/z* 864.5 with characteristic fragments. The presence of hydroxyl group is confirmed by the ion cluster at *m/z* 568, 540, and 522 (typical for sphingosine base 18:1) and *m/z* 540, 512, and 494 (typical for sphingosine base 16:1).

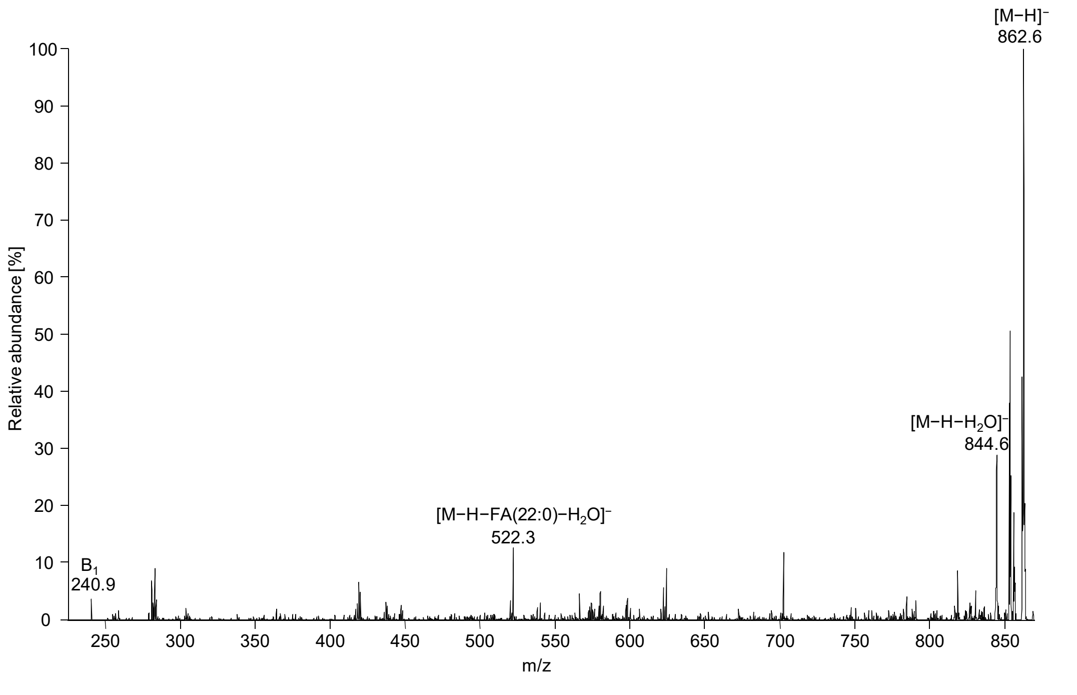

Figure S95. MS/MS spectrum of SHexCer 40:1 at *m/z* 862.6 with characteristic fragments.

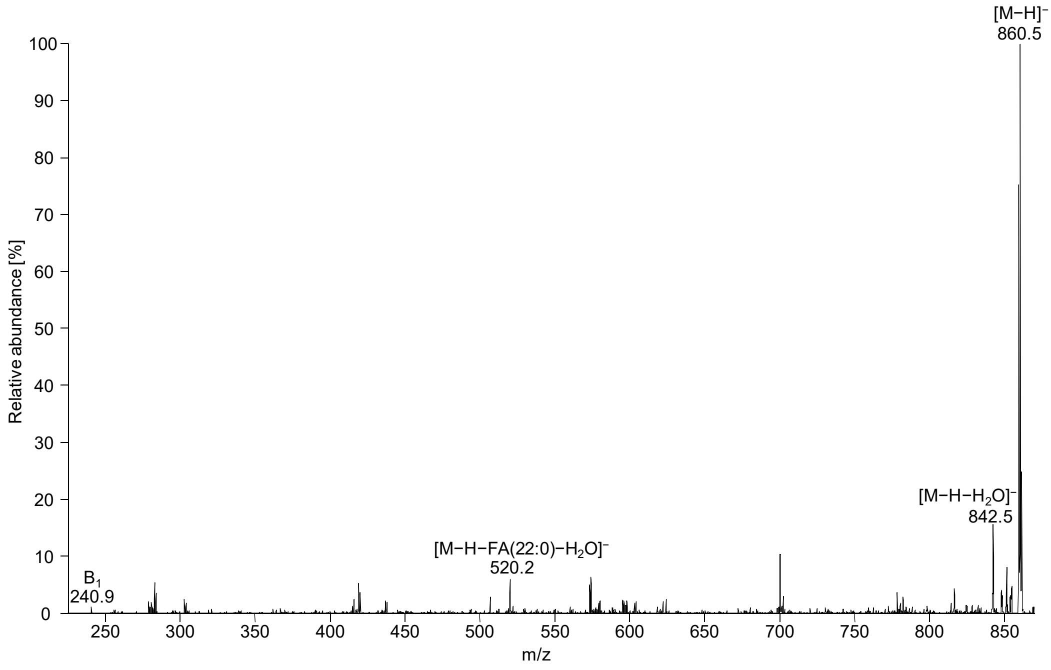

Figure S96. MS/MS spectrum of SHexCer 40:2 at *m/z* 860.5 with characteristic fragments.

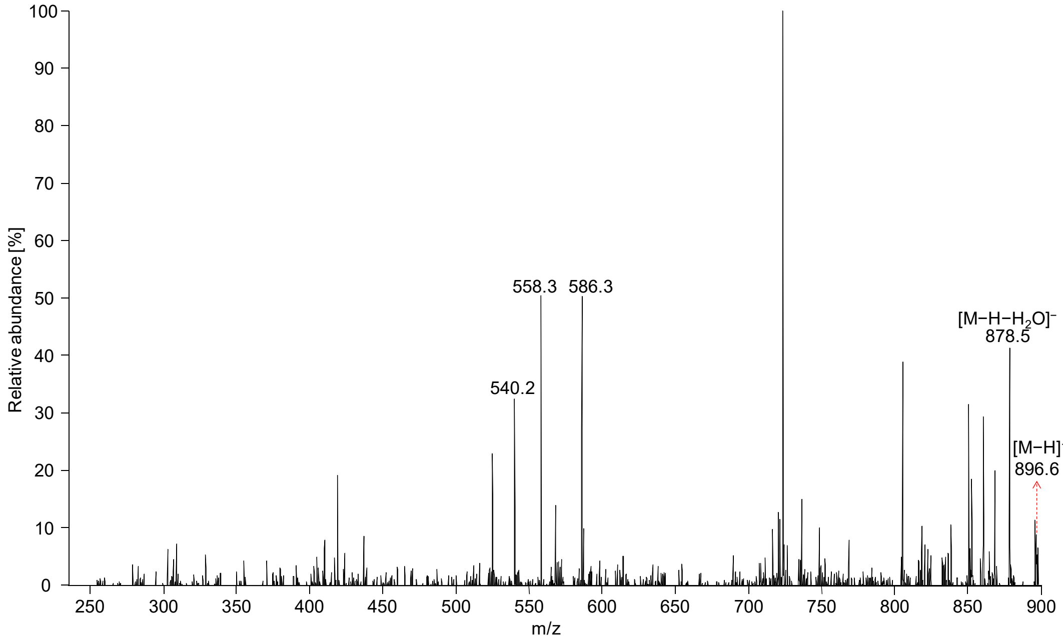

Figure S97. MS/MS spectrum of SHexCer 40:0 2OH at *m/z* 896.6 with characteristic fragments. The presence of two hydroxyl group is confirmed by the ion cluster at *m/z* 586, 558, and 540 (typical for sphingosine base 18:1 OH).

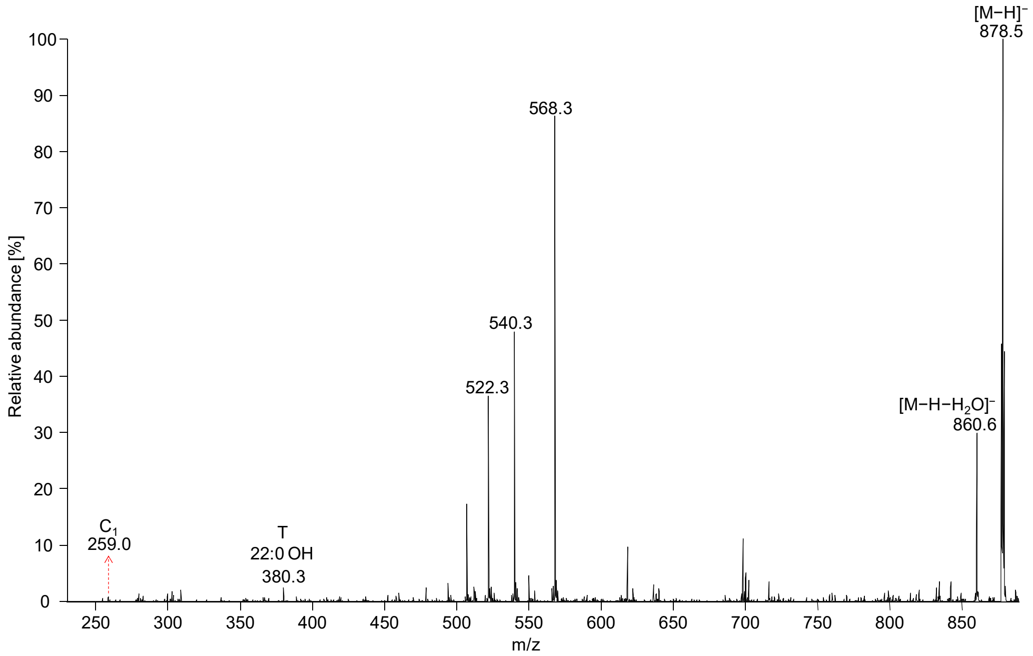

Figure S98. MS/MS spectrum of SHexCer 40:1 OH at *m/z* 878.5 with characteristic fragments. The presence of hydroxyl group is confirmed by the ion cluster at *m/z* 568, 540, and 522 (typical for sphingosine base 18:1).

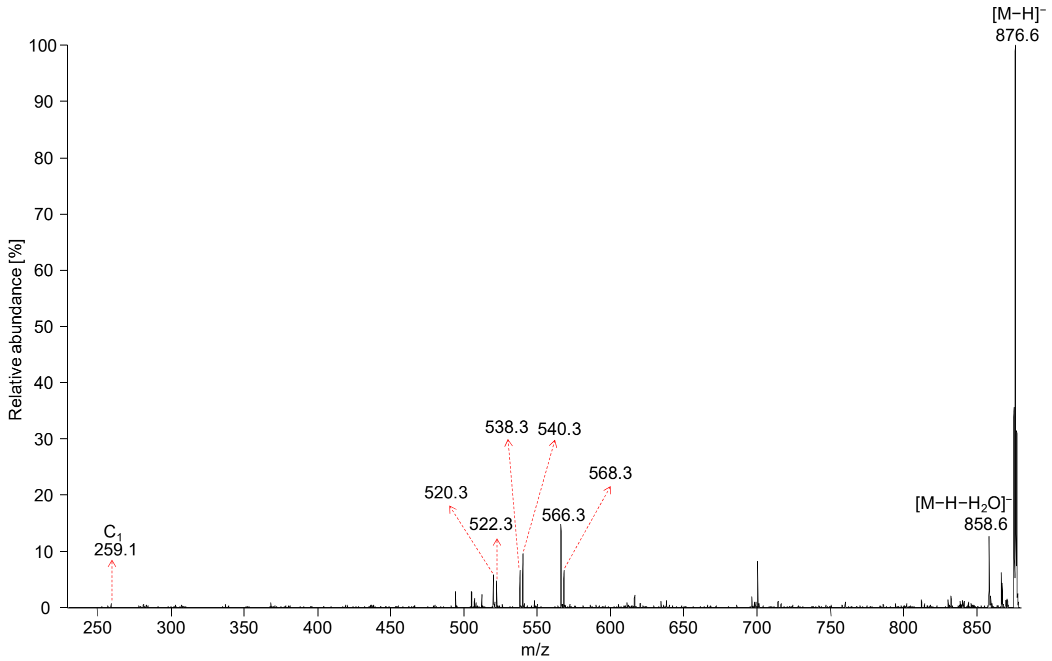

Figure S99. MS/MS spectrum of SHexCer 40:2 OH at *m/z* 876.6 with characteristic fragments. The presence of hydroxyl group is confirmed by the ion cluster at *m/z* 568, 540, and 522 (typical for sphingosine base 18:1) and *m/z* 566, 538, and 520 (typical for sphingosine base 18:2).

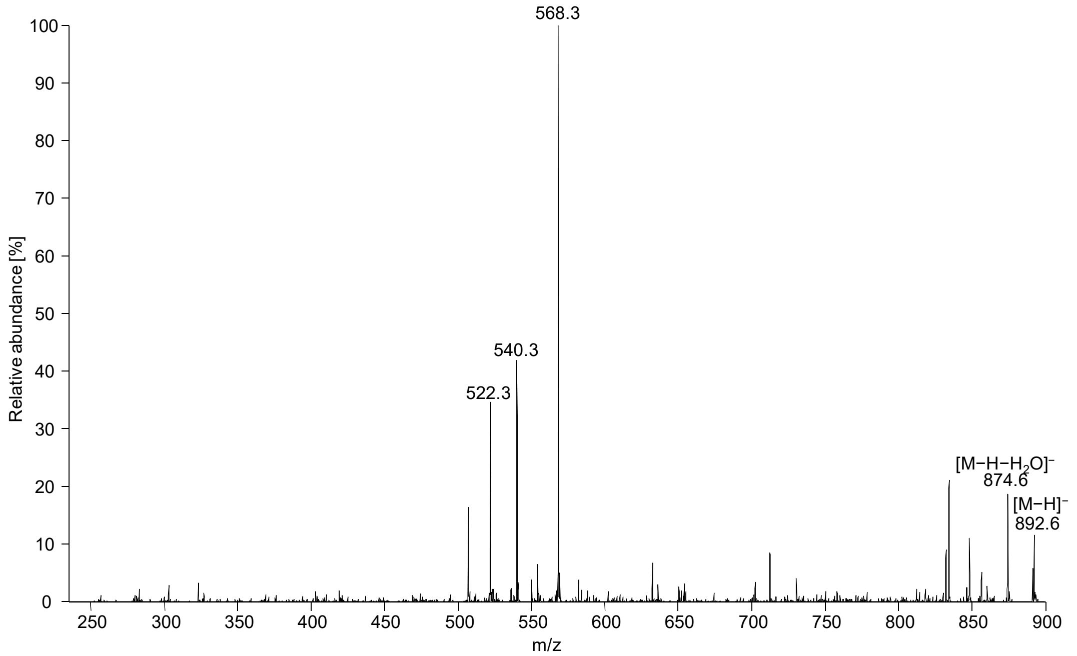

Figure S100. MS/MS spectrum of SHexCer 41:1 OH at *m/z* 892.6 with characteristic fragments. The presence of hydroxyl group is confirmed by the ion cluster at *m/z* 568, 540, and 522 (typical for sphingosine base 18:1).

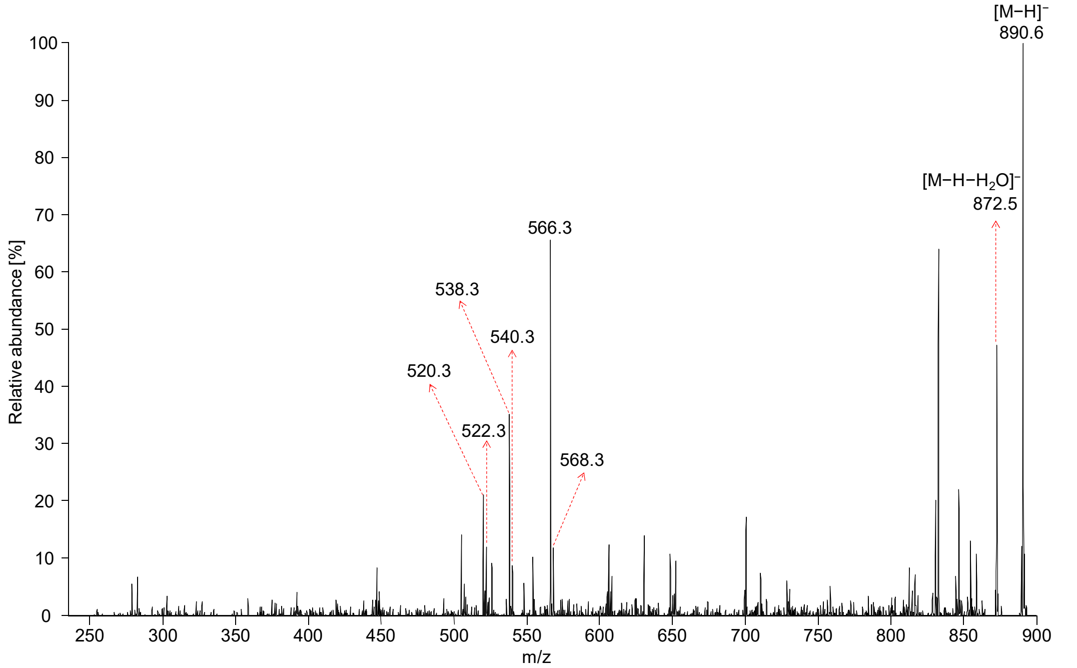

Figure S101. MS/MS spectrum of SHexCer 41:2 OH at *m/z* 890.6 with characteristic fragments. The presence of hydroxyl group is confirmed by the ion cluster at *m/z* 568, 540, and 522 (typical for sphingosine base 18:1) and *m/z* 566, 538, and 520 (typical for sphingosine base 18:2).

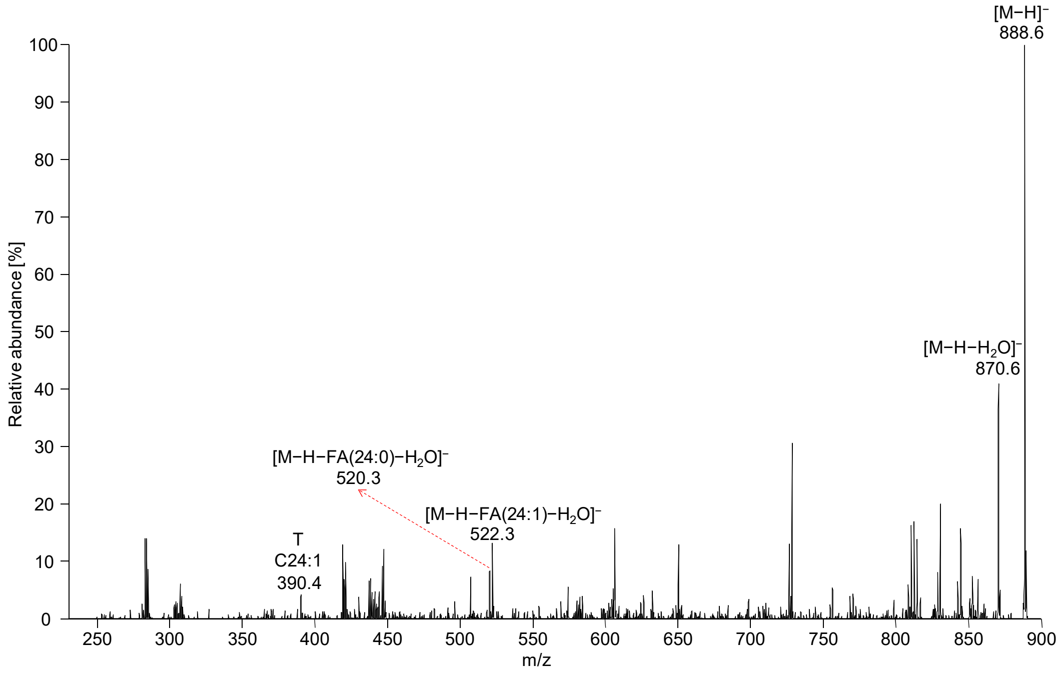

Figure S102. MS/MS spectrum of SHexCer 42:2 at *m/z* 888.6 with characteristic fragments.

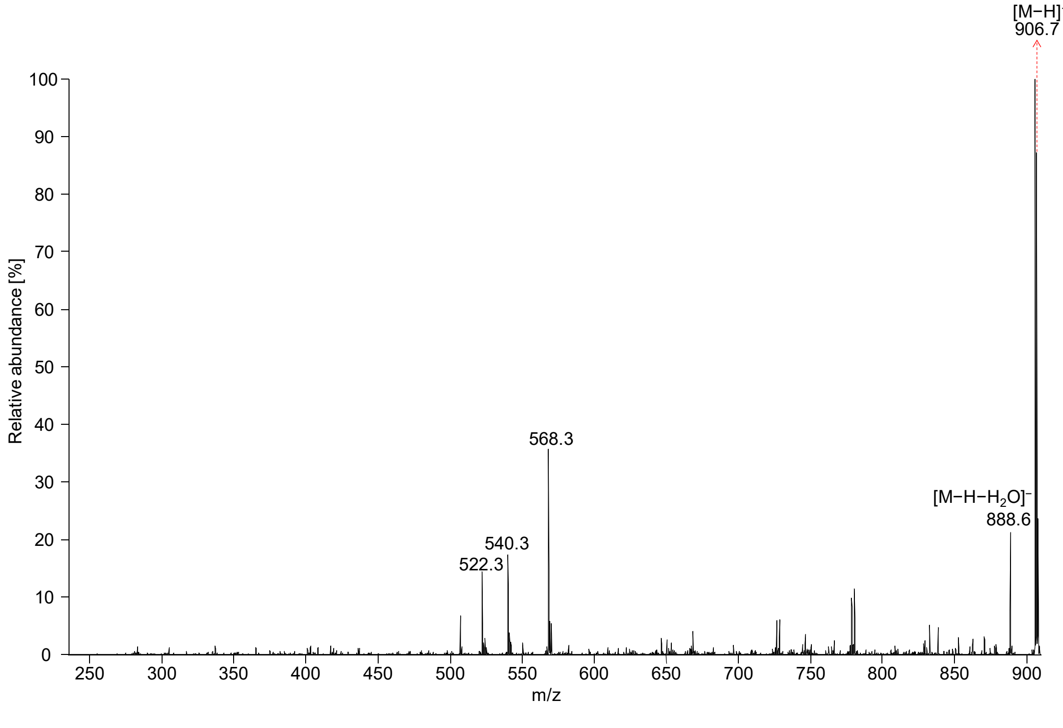

Figure S103. MS/MS spectrum of SHexCer 42:1 OH at *m/z* 906.7 with characteristic fragments. The presence of hydroxyl group is confirmed by the ion cluster at *m/z* 568, 540, and 522 (typical for sphingosine base 18:1).

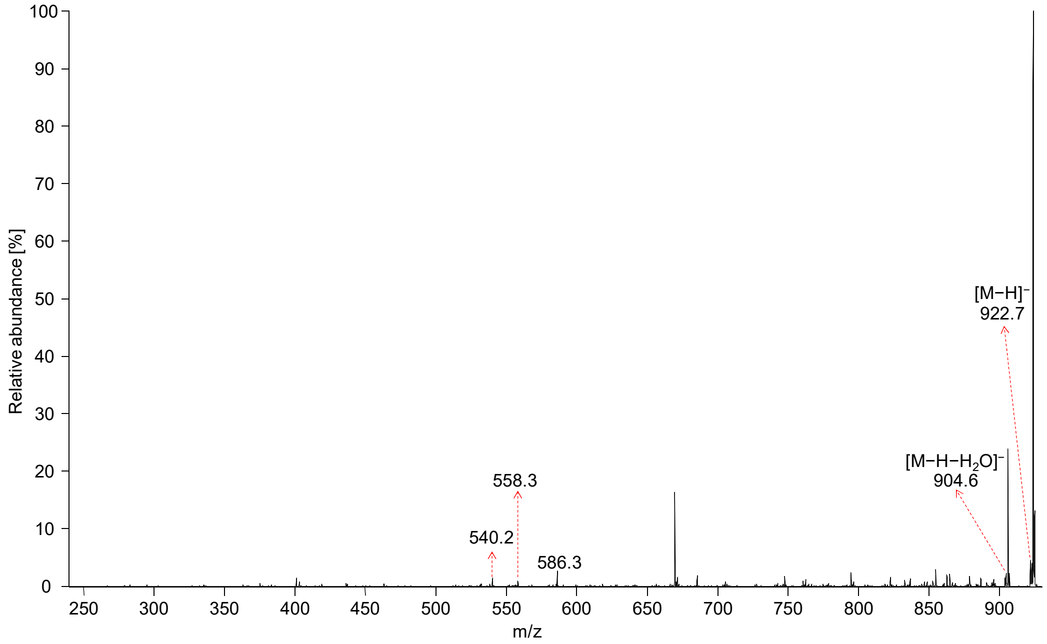

Figure S104. MS/MS spectrum of SHexCer 42:1 2OH at *m/z* 922.7 with characteristic fragments. The presence of two hydroxyl group is confirmed by the ion cluster at *m/z* 586, 558, and 540 (typical for sphingosine base 18:1 OH).

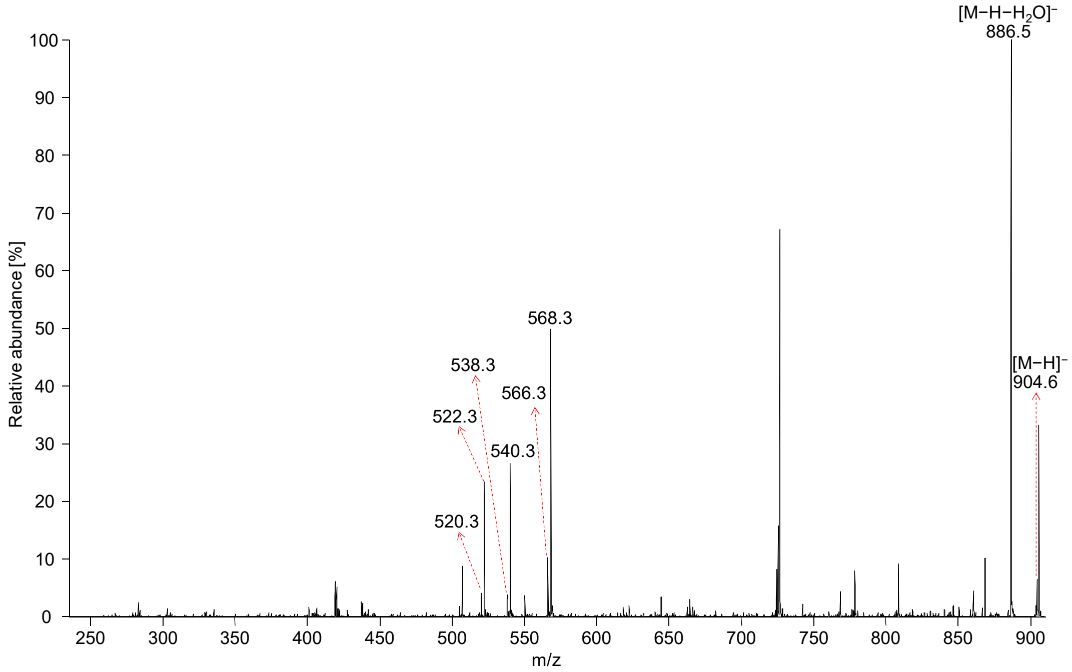

Figure S105. MS/MS spectrum of SHexCer 42:2 OH at *m/z* 904.6 with characteristic fragments. The presence of hydroxyl group is confirmed by the ion cluster at *m/z* 568, 540, and 522 (typical for sphingosine base 18:1) and *m/z* 566, 538, and 520 (typical for sphingosine base 18:2).

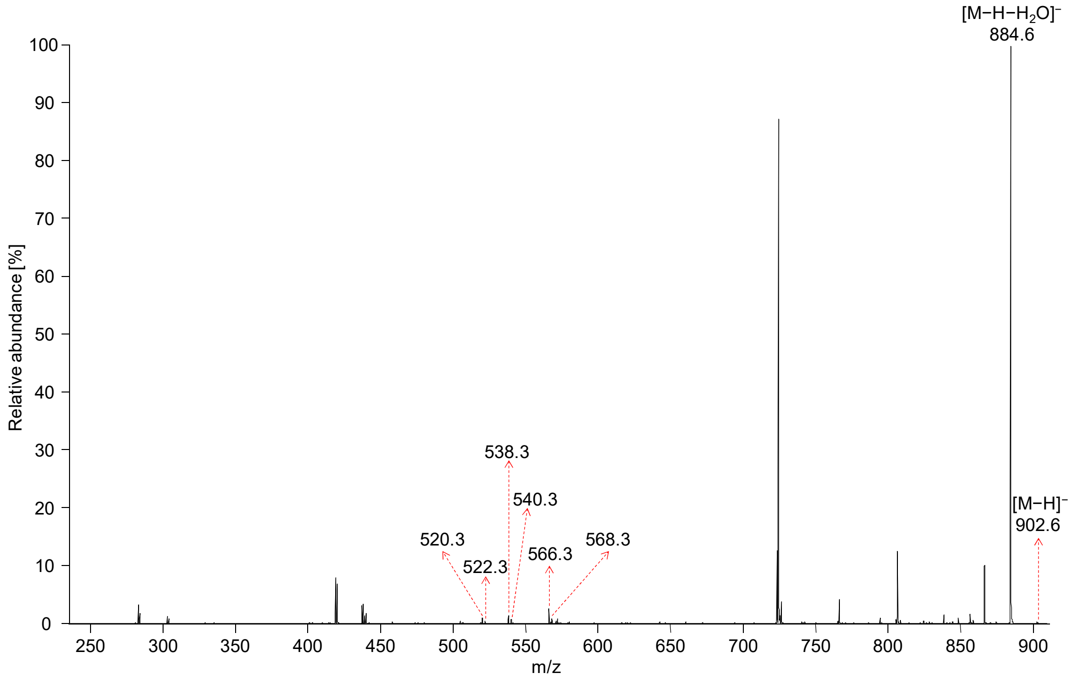

Figure S106. MS/MS spectrum of SHexCer 42:3 OH at *m/z* 902.6 with characteristic fragments. The presence of hydroxyl group is confirmed by the ion cluster at *m/z* 568, 540, and 522 (typical for sphingosine base 18:1) and *m/z* 566, 538, and 520 (typical for sphingosine base 18:2).

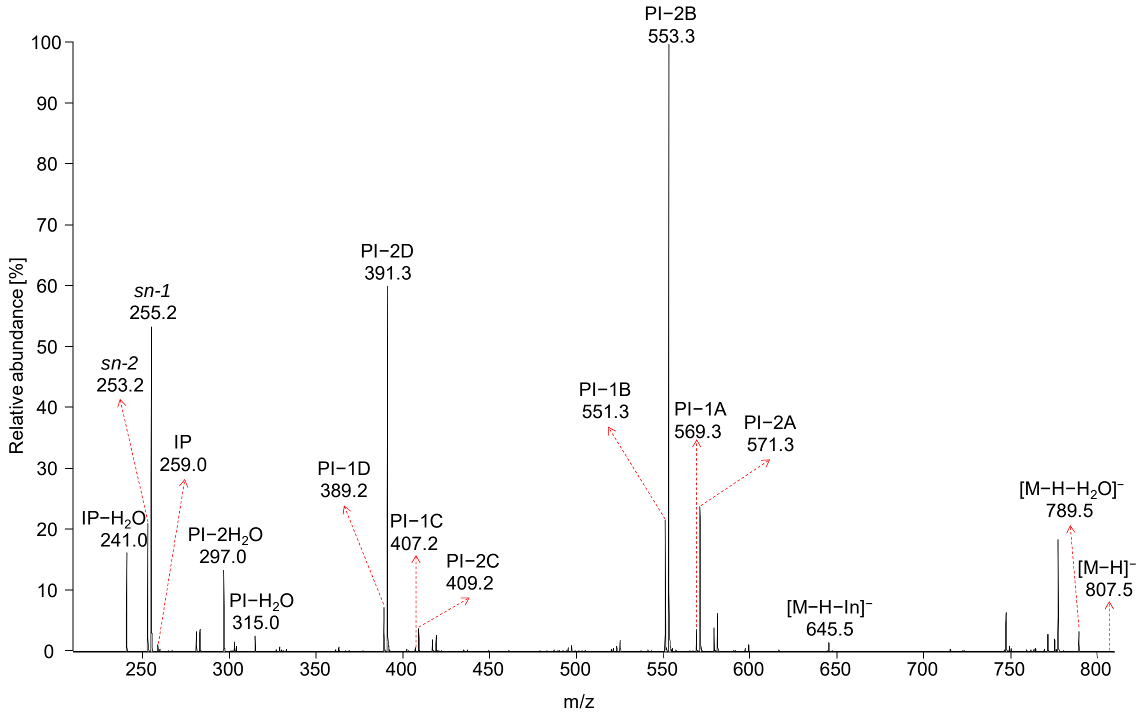

Figure S107. MS/MS spectrum of PI 32:1 at *m/z* 807.5 with characteristic fragments.

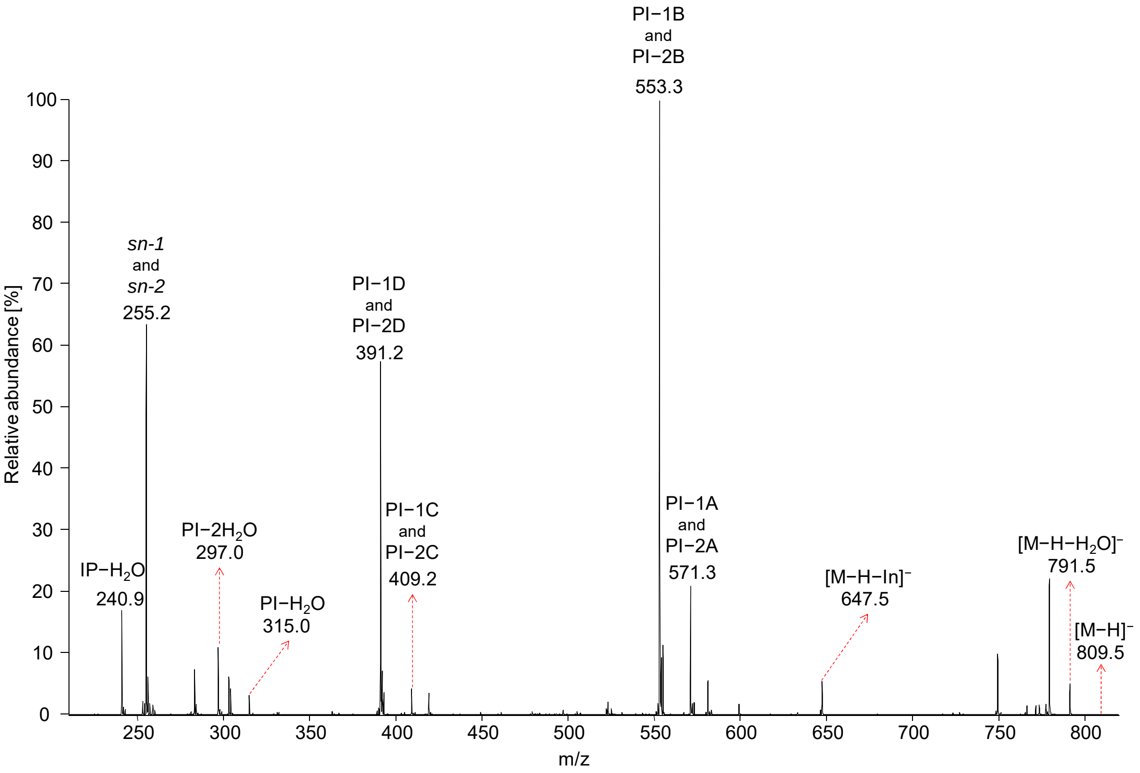

Figure S108. MS/MS spectrum of PI 32:0 at *m/z* 809.5 with characteristic fragments.

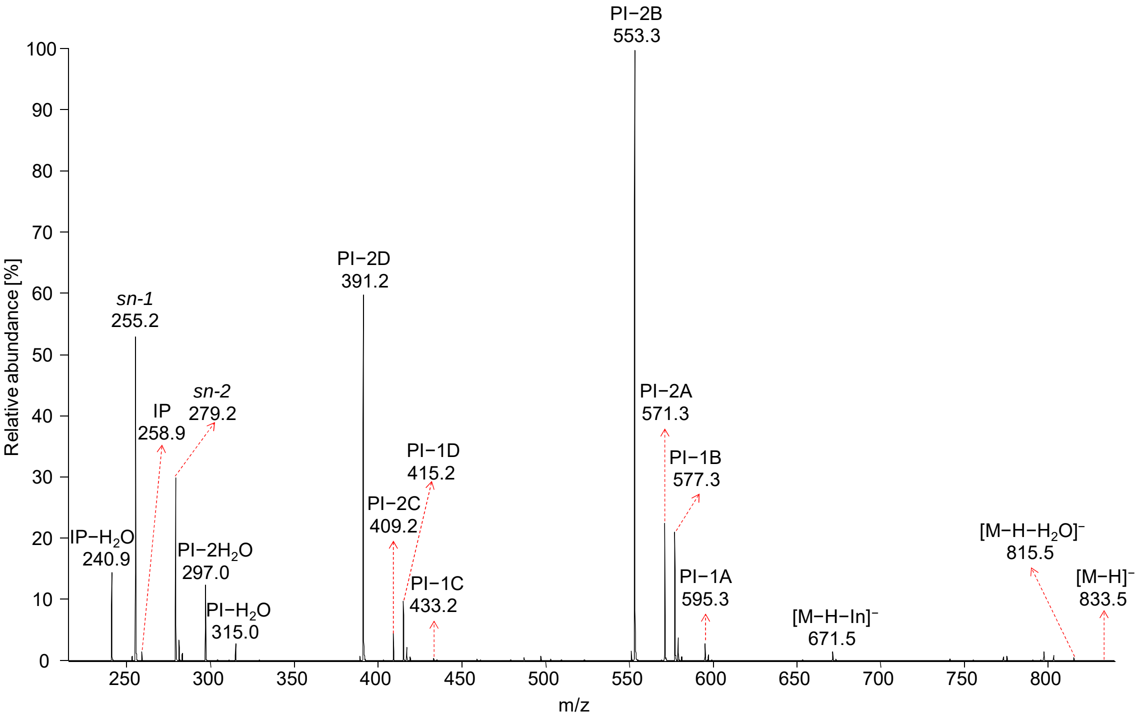

Figure S109. MS/MS spectrum of PI 34:2 at *m/z* 833.5 with characteristic fragments.

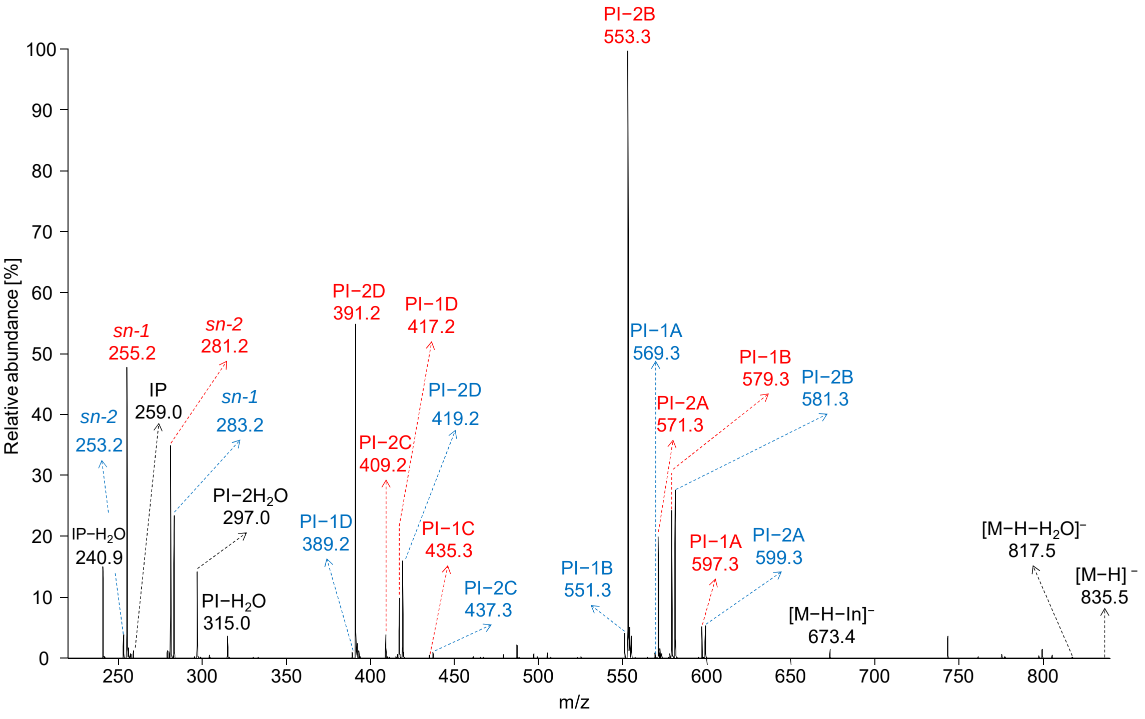

Figure S110. MS/MS spectrum of PI 34:1 at *m/z* 835.5 with characteristic fragments. The specific fragments for PI 16:0/18:1 (red color) and PI 18:0/16:1 (blue color) are shown.

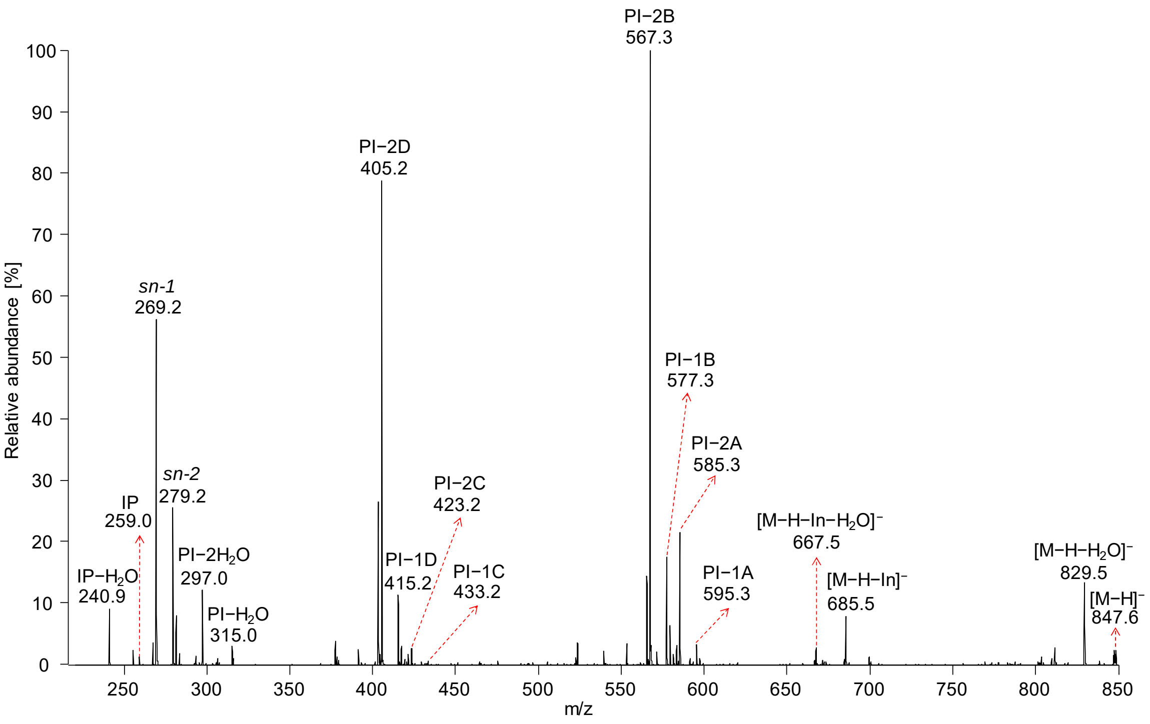

Figure S111. MS/MS spectrum of PI 35:2 at *m/z* 847.6 with characteristic fragments.

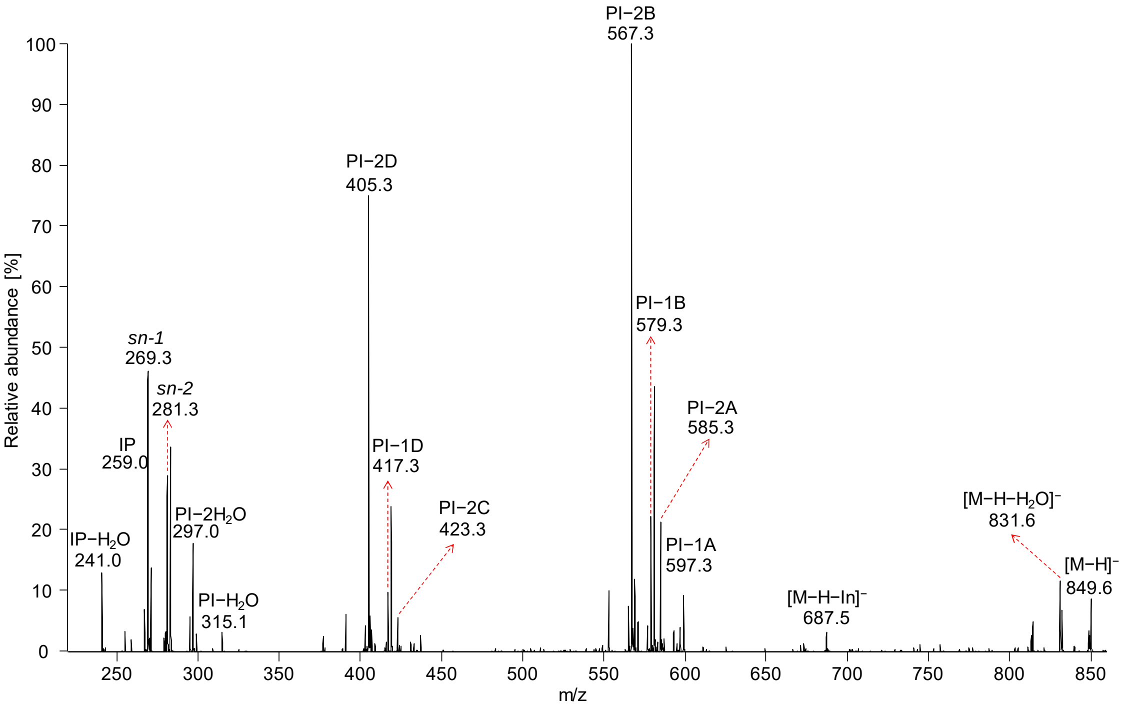

Figure S112. MS/MS spectrum of PI 35:1 at *m/z* 849.6 with characteristic fragments.

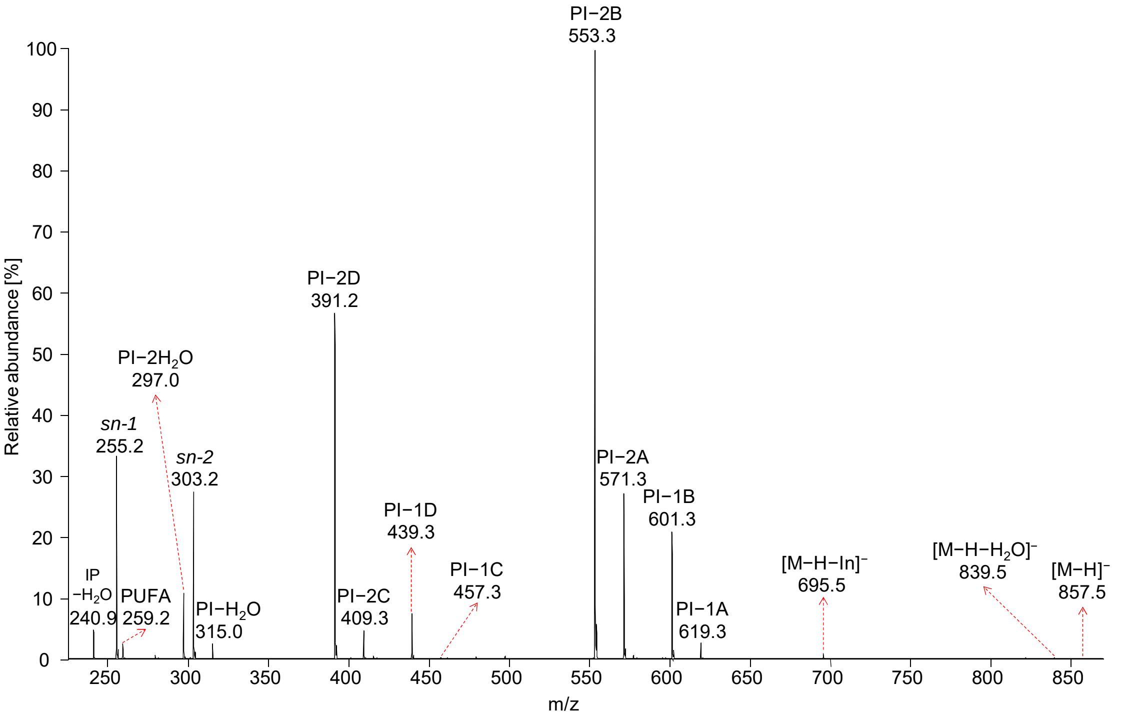

Figure S113. MS/MS spectrum of PI 36:4 at *m/z* 857.5 with characteristic fragments.

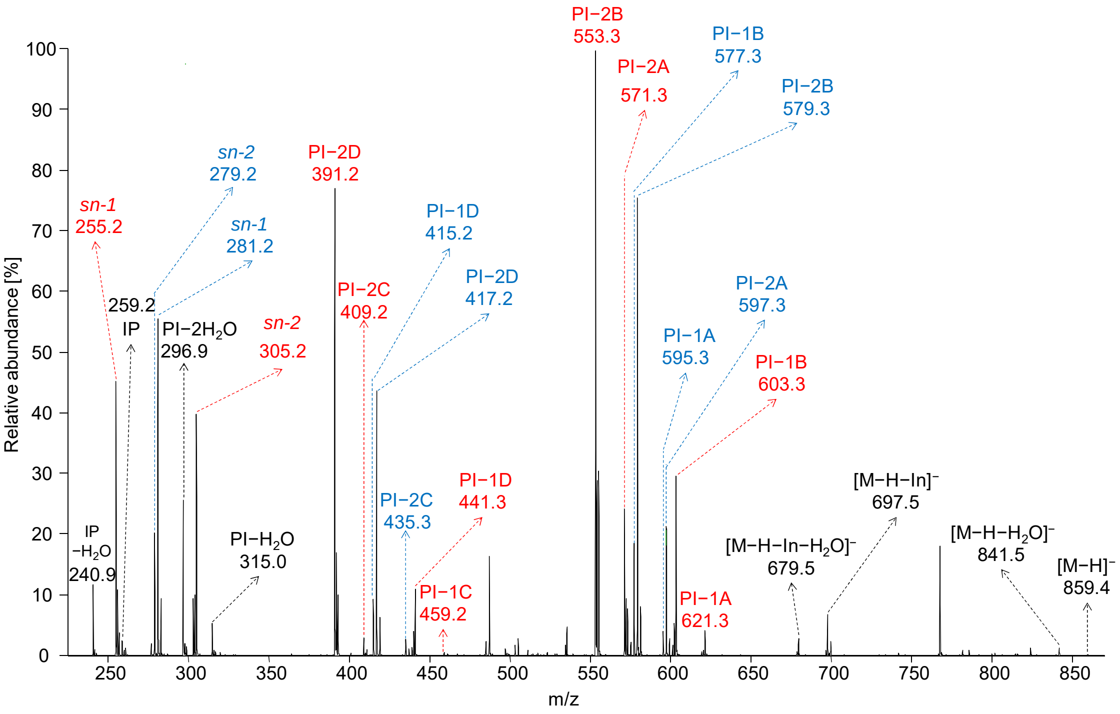

Figure S114. MS/MS spectrum of PI 36:3 at *m/z* 859.4 with characteristic fragments. The specific fragments for PI 16:0/20:3 (red color) and PI 18:1/18:2 (blue color) are shown.

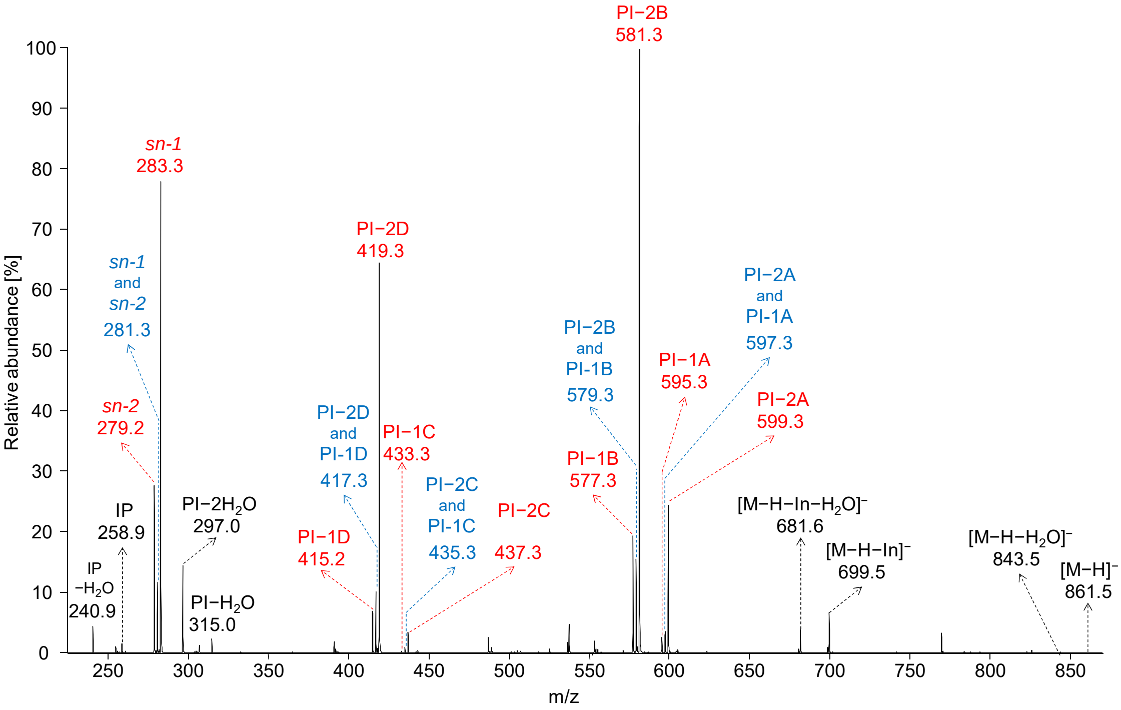

Figure S115. MS/MS spectrum of PI 36:2 at *m/z* 861.5 with characteristic fragments. The specific fragments for PI 18:0/18:2 (red color) and PI 18:1/18:1 (blue color) are shown.

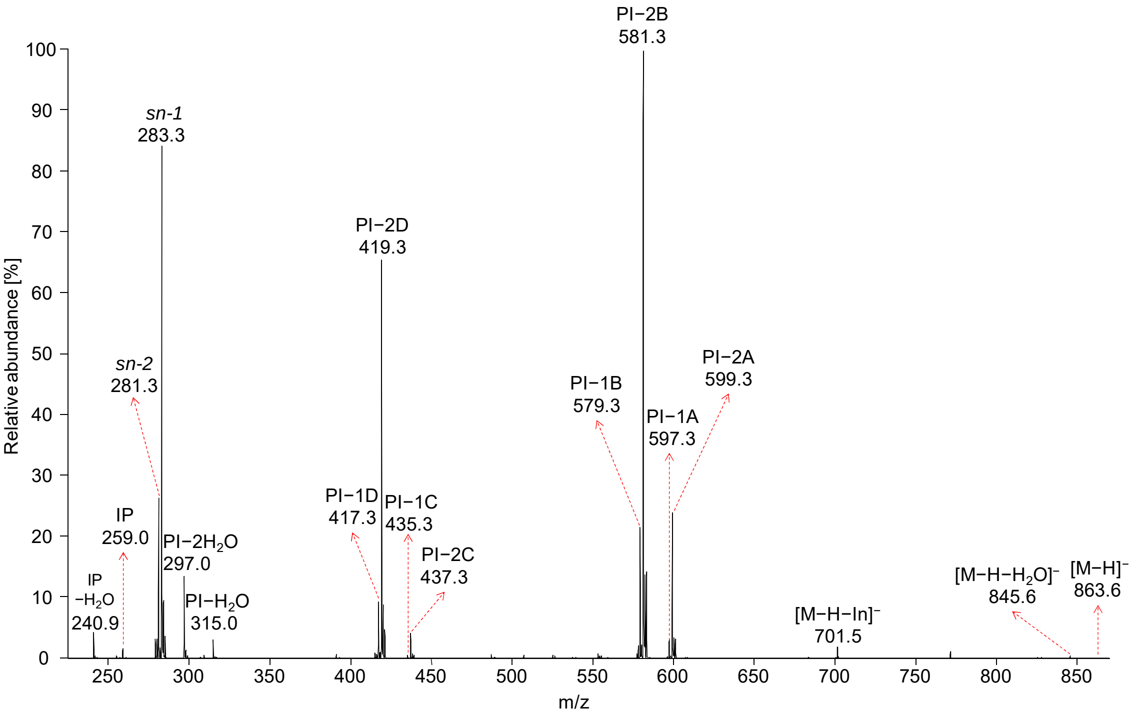

Figure S116. MS/MS spectrum of PI 36:1 at *m/z* 863.6 with characteristic fragments.

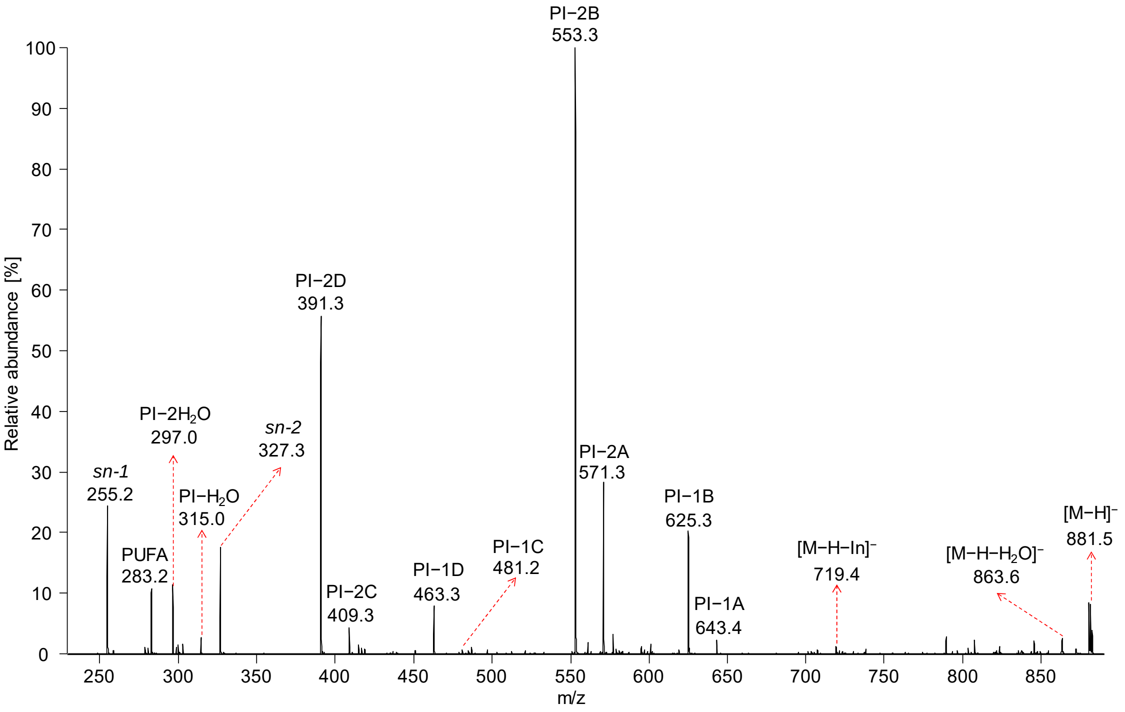

Figure S117. MS/MS spectrum of PI 38:6 at *m/z* 881.5 with characteristic fragments.

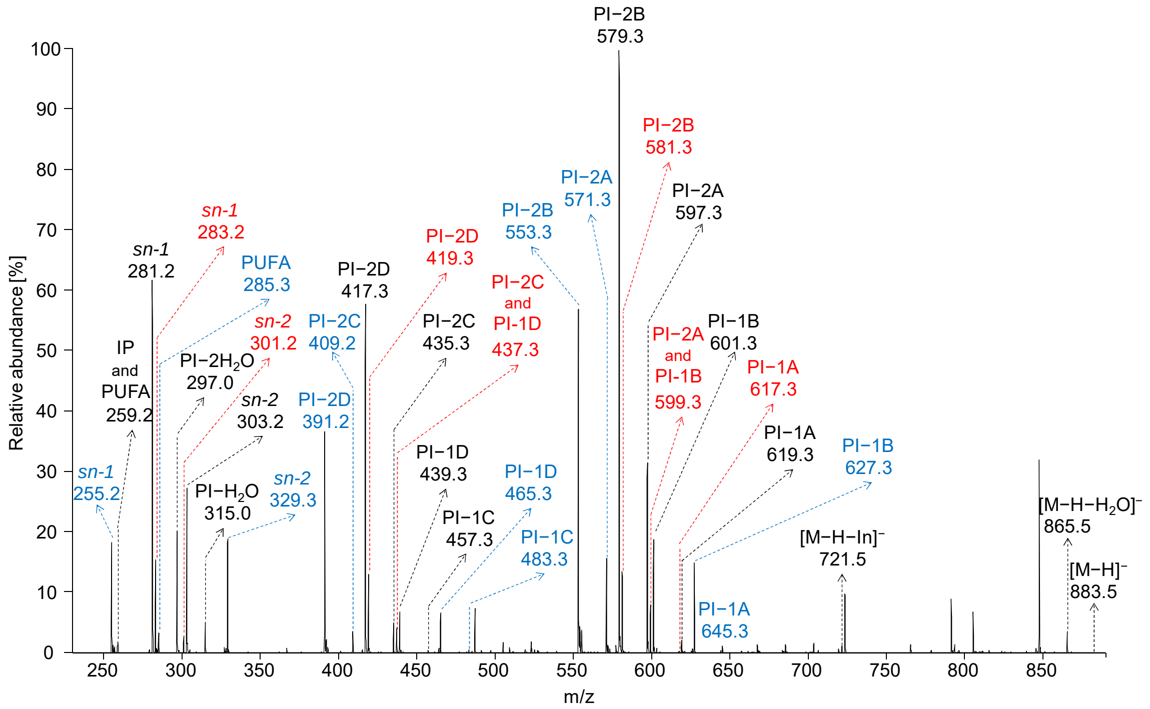

Figure S118. MS/MS spectrum of PI 38:5 at *m/z* 883.5 with characteristic fragments. The specific fragments for PI 18:1/20:4 (black color), PI 18:0/20:5 (red color), and PI 16:0/22:5 (blue color) are shown.

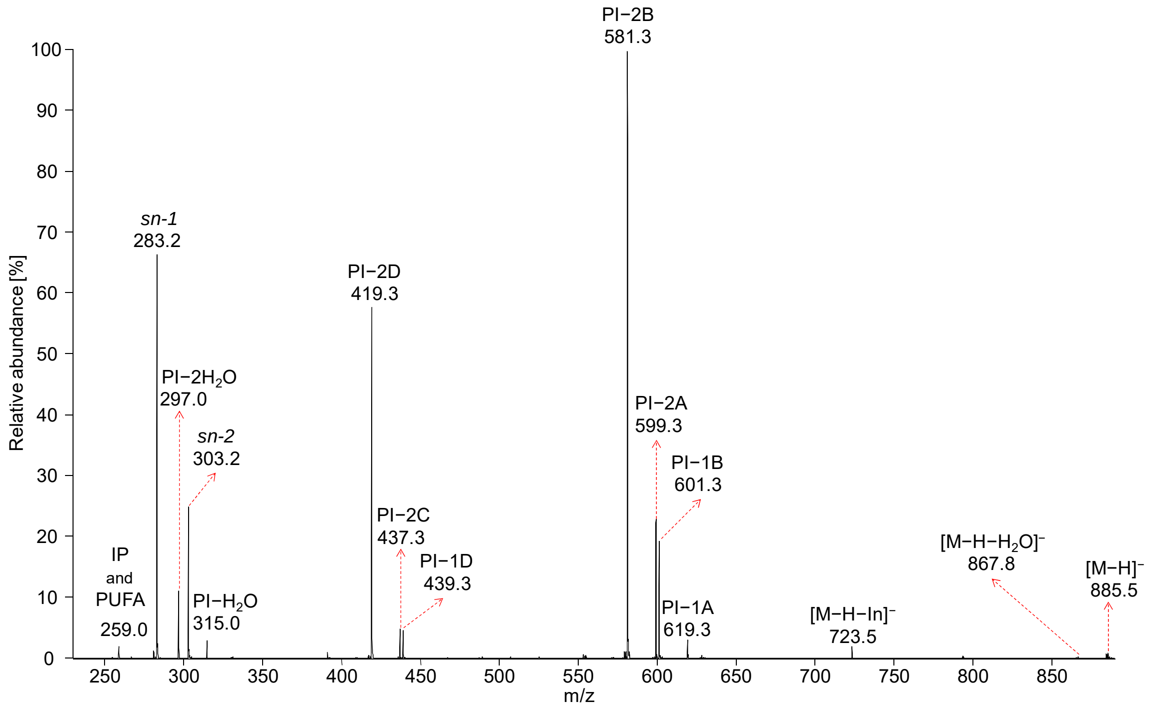

Figure S119. MS/MS spectrum of PI 38:4 at *m/z* 885.5 with characteristic fragments.

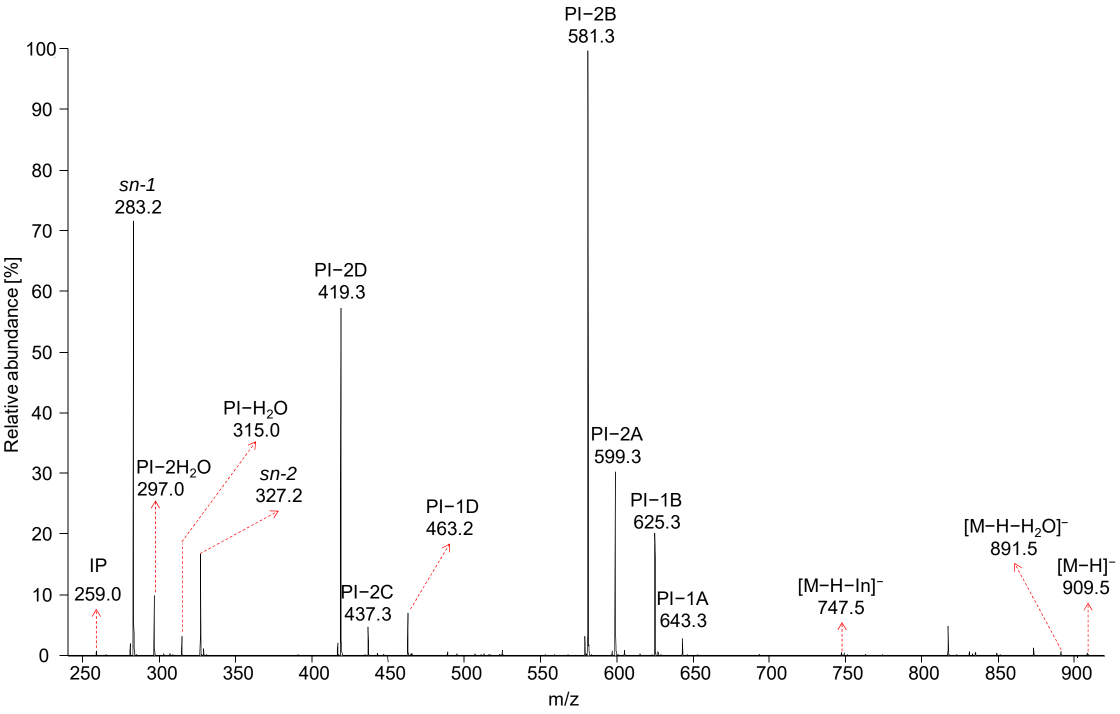

Figure S120. MS/MS spectrum of PI 40:6 at *m/z* 909.5 with characteristic fragments.

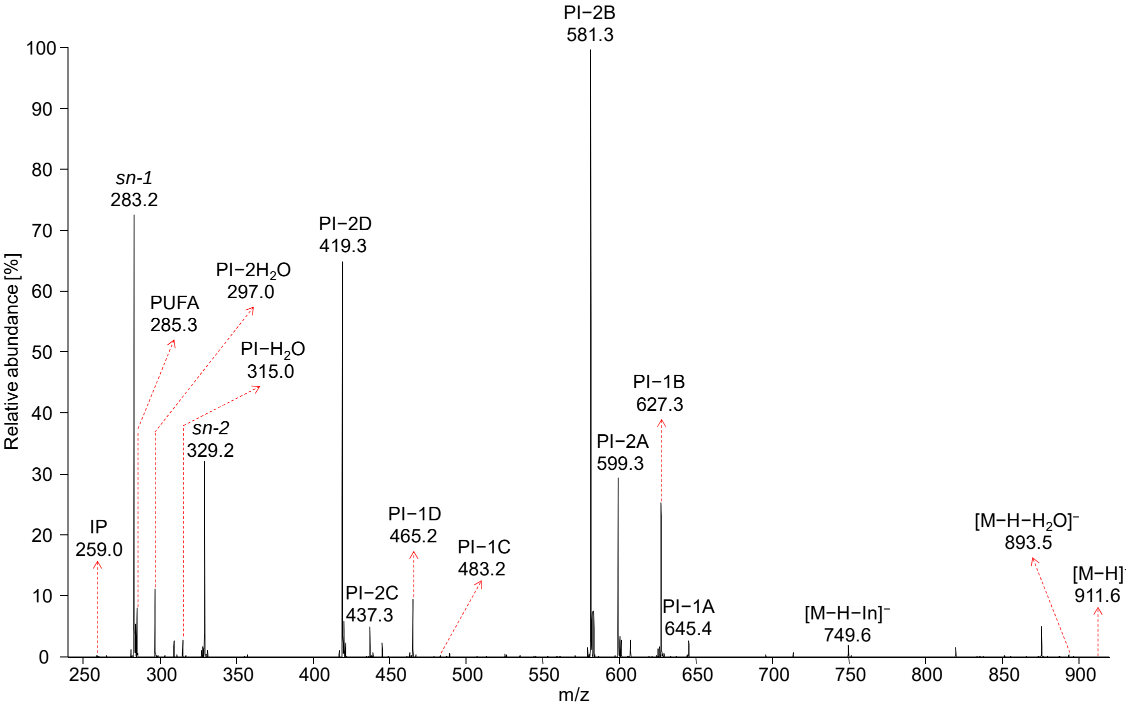

Figure S121. MS/MS spectrum of PI 40:5 at *m/z* 911.6 with characteristic fragments.

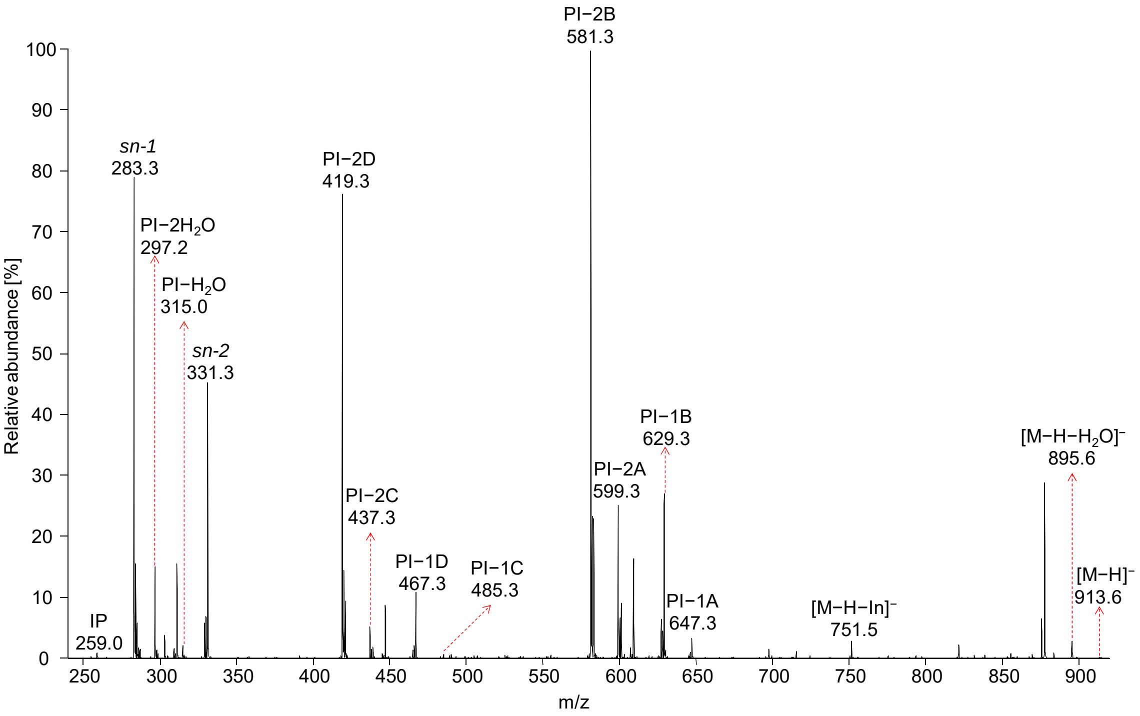

Figure S122. MS/MS spectrum of PI 40:4 at *m/z* 913.6 with characteristic fragments.

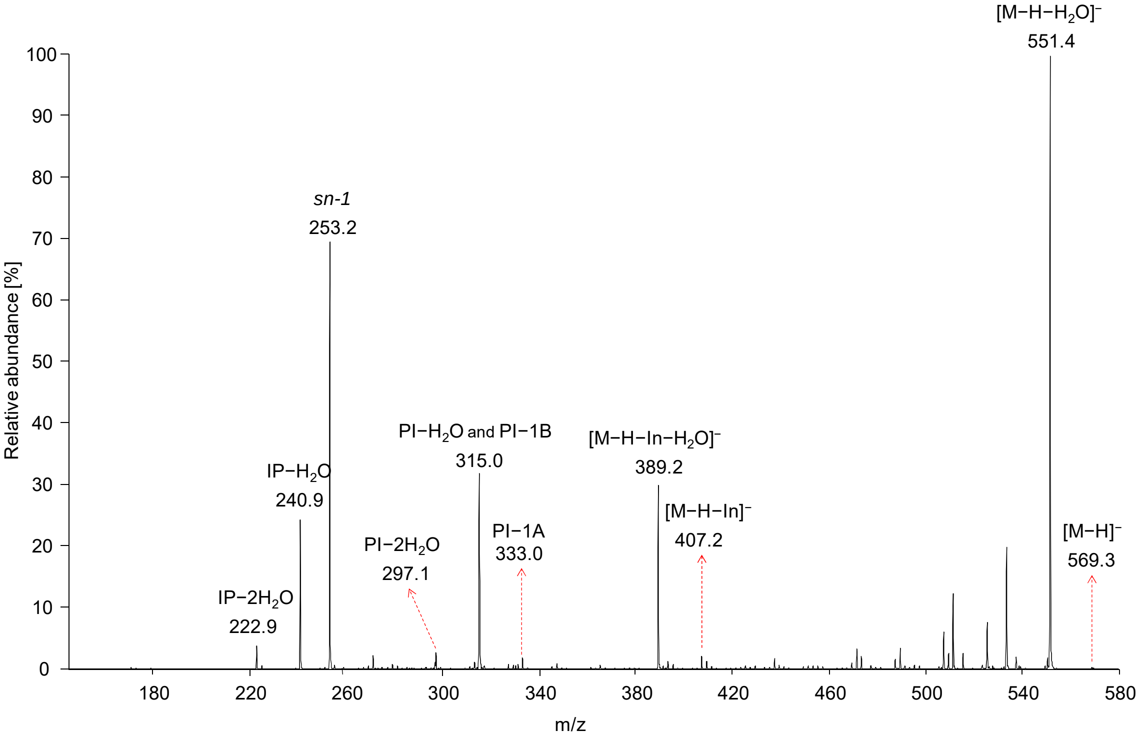

Figure S123. MS/MS spectrum of LPI 16:1 at *m/z* 569.3 with characteristic fragments.

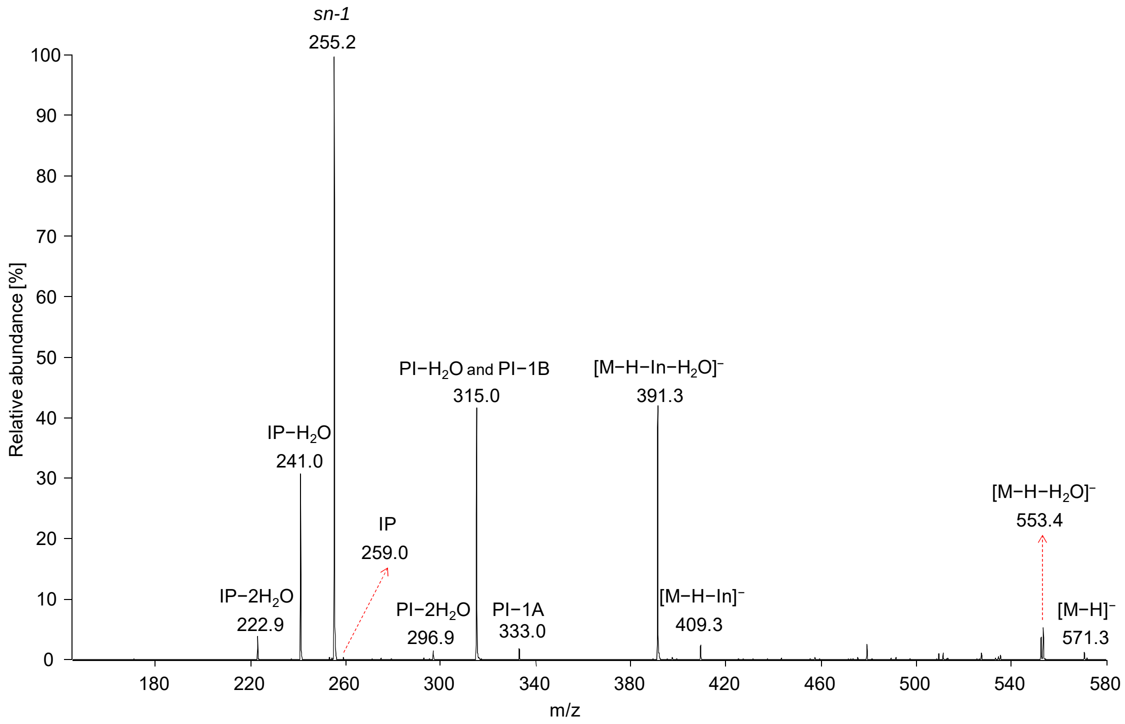

Figure S124. MS/MS spectrum of LPI 16:0 at *m/z* 571.3 with characteristic fragments.

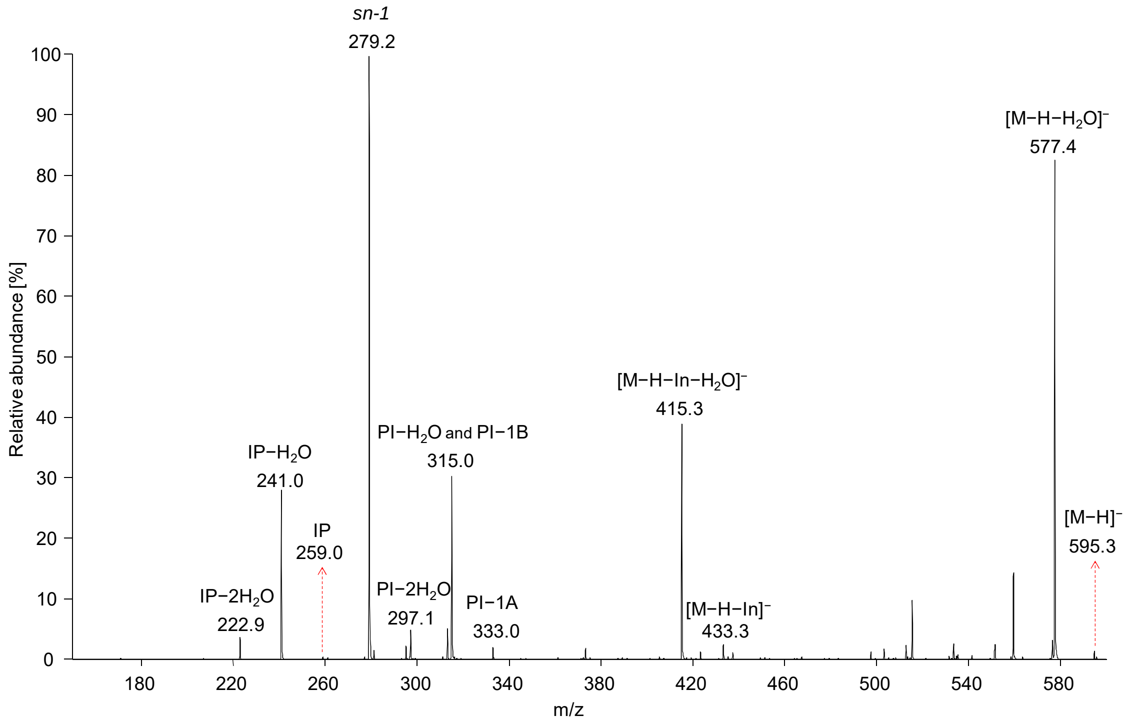

Figure S125. MS/MS spectrum of LPI 18:2 at *m/z* 595.3 with characteristic fragments.

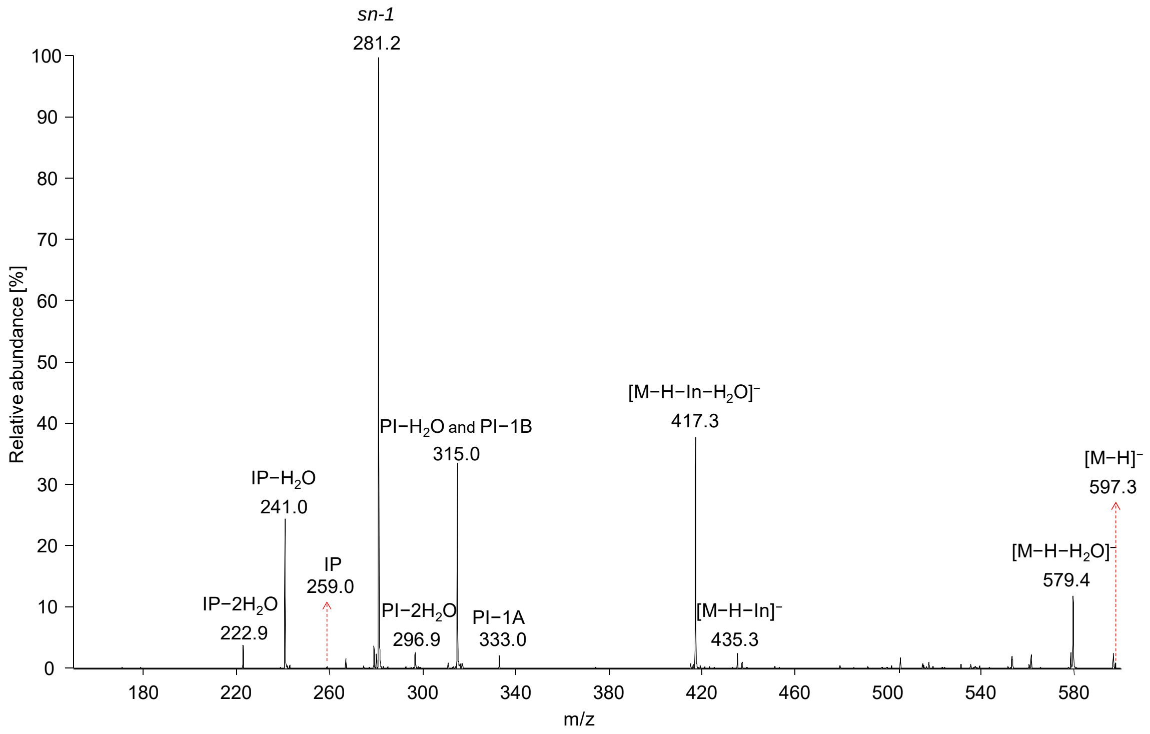

Figure S126. MS/MS spectrum of LPI 18:1 at *m/z* 597.3 with characteristic fragments.

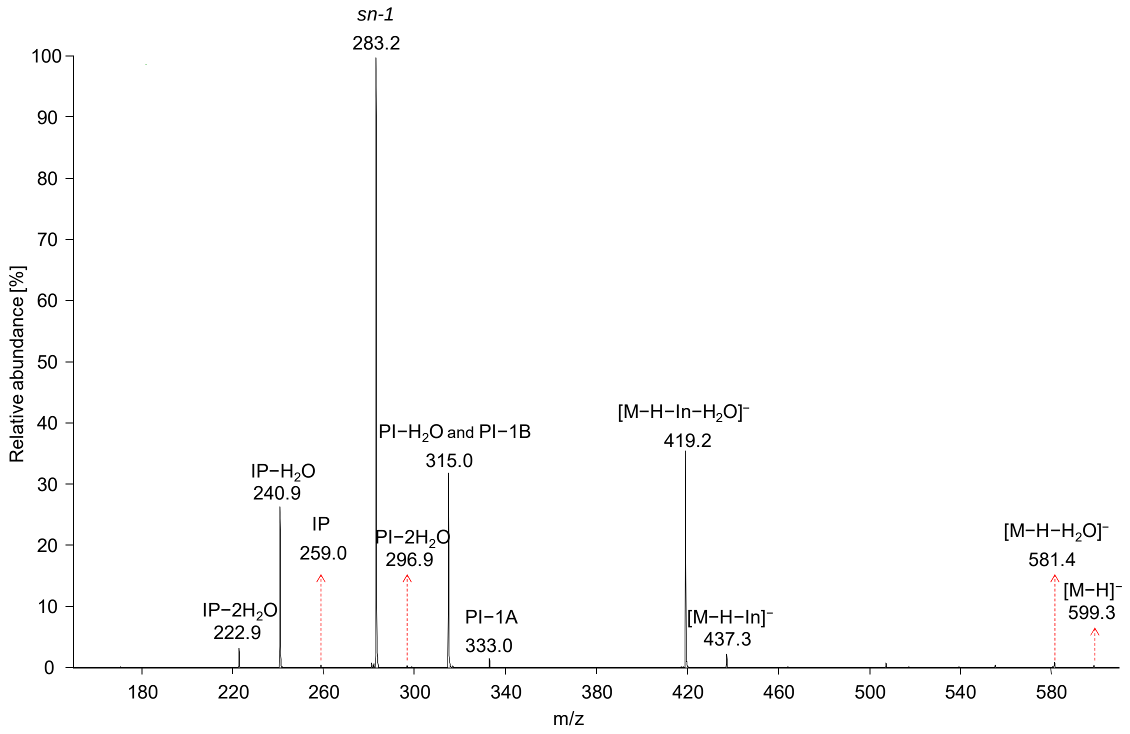

Figure S127. MS/MS spectrum of LPI 18:0 at *m/z* 599.3 with characteristic fragments.

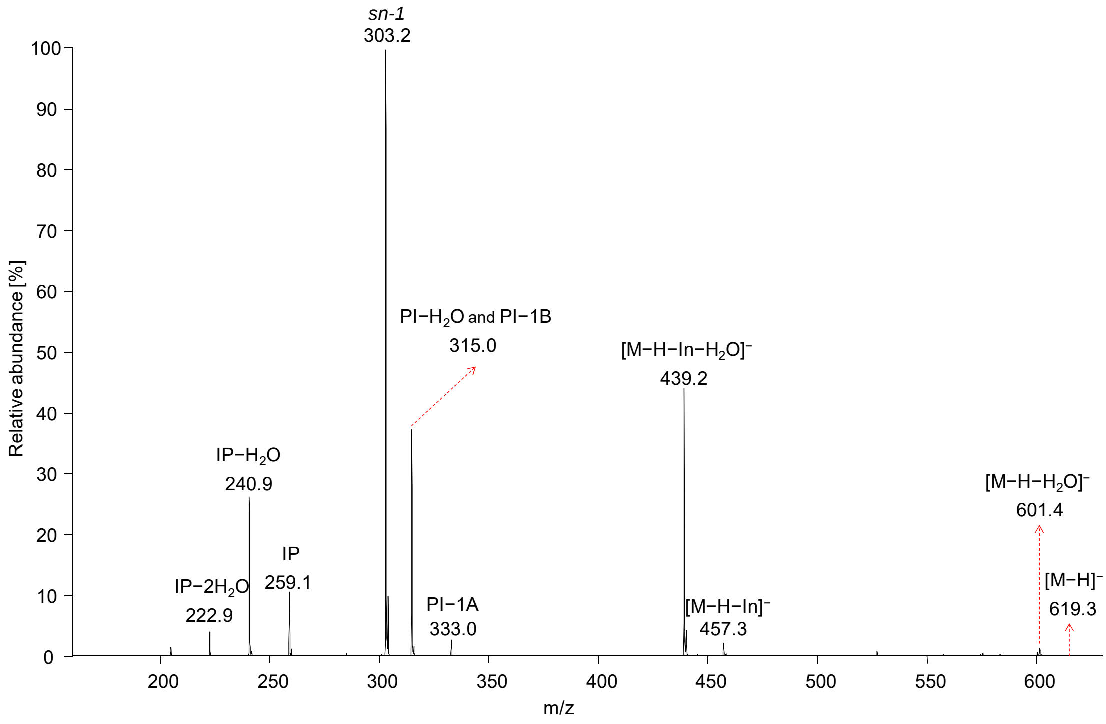

Figure S128. MS/MS spectrum of LPI 20:4 at *m/z* 619.3 with characteristic fragments.

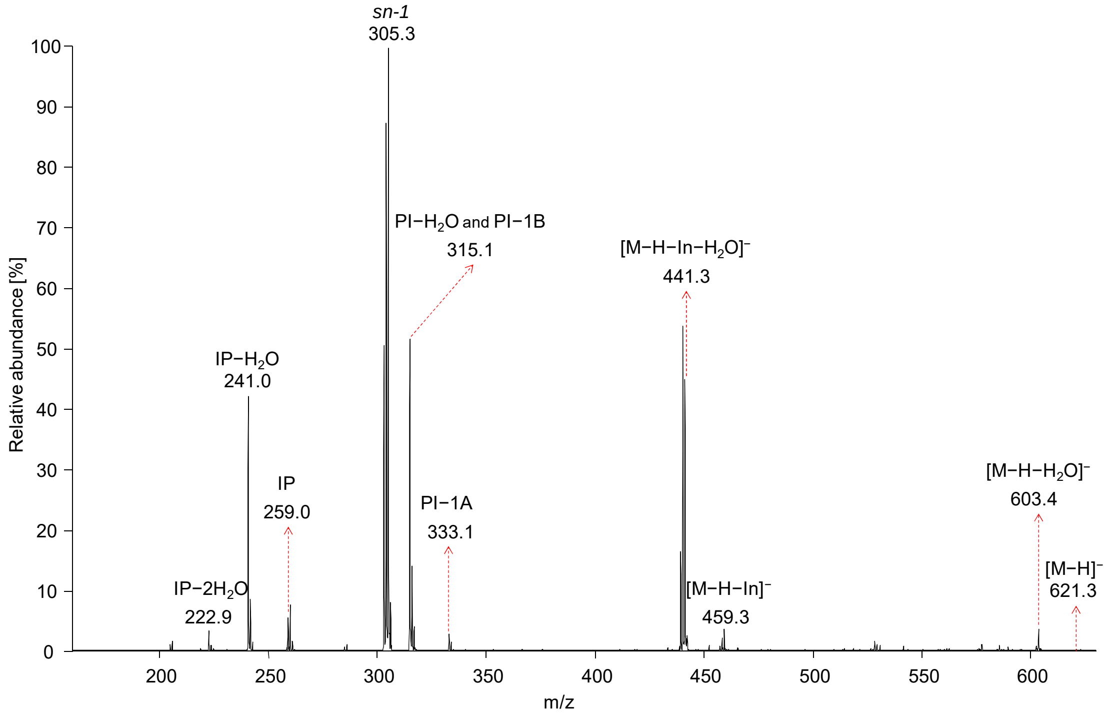

Figure S129. MS/MS spectrum of LPI 20:3 at *m/z* 621.3 with characteristic fragments.

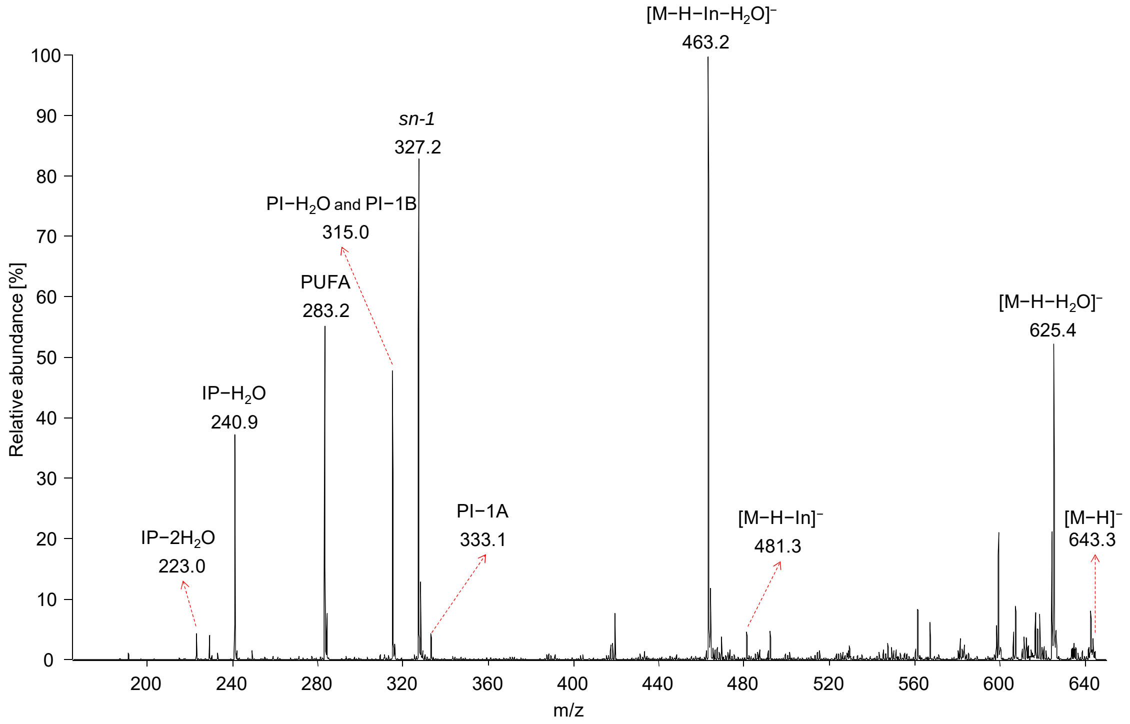

Figure S130. MS/MS spectrum of LPI 22:6 at *m/z* 643.3 with characteristic fragments.

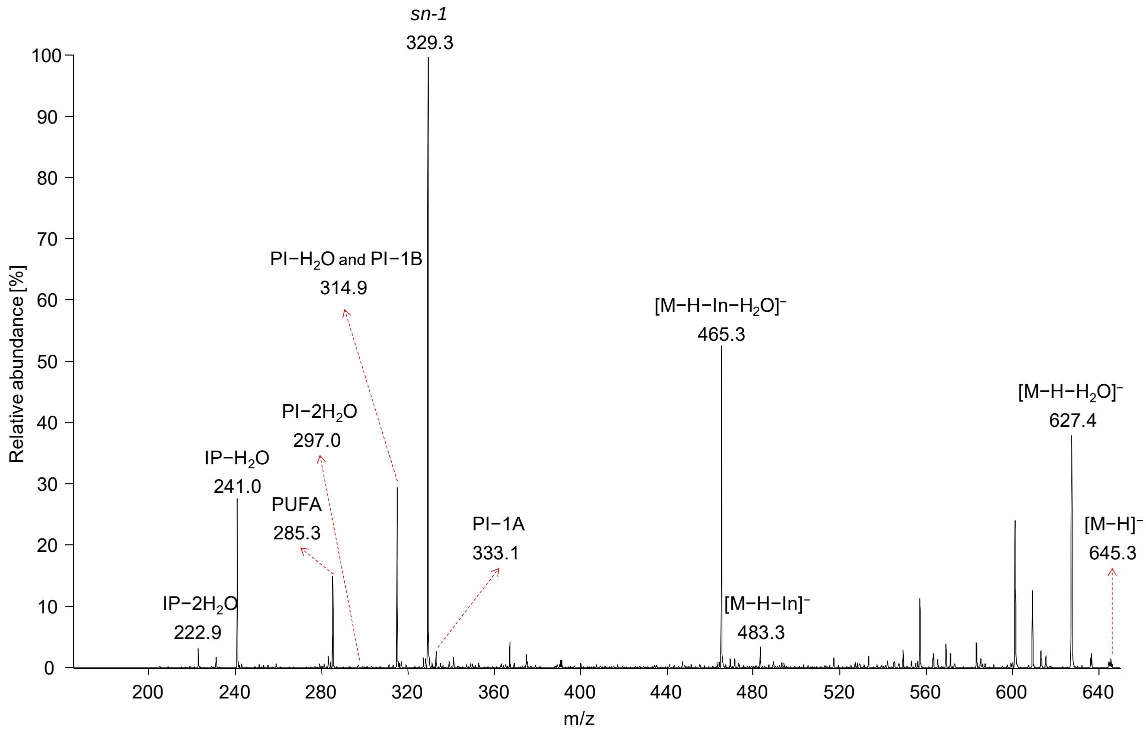

Figure S131. MS/MS spectrum of LPI 22:5 at *m/z* 645.3 with characteristic fragments.
